# Supplementary material for: Accessible light-controlled knockdown of cell-free protein synthesis using phosphorothioate-caged antisense oligonucleotides
Source: Commun Chem. 2023 Apr 1;6:59. doi: 10.1038/s42004-023-00860-2 (PMC10067960; doi:10.1038/s42004-023-00860-2)
Supplement: Supplementary file 2 — Supplementary Information [file 42004_2023_860_MOESM2_ESM.pdf]

Supplementary Information

Title: Accessible light-controlled knockdown of cell-free protein synthesis using phosphorothioate-caged antisense oligonucleotides

Authors: Denis Hartmann[1], Michael J. Booth[1,2]*

Affiliations:

[1]Department of Chemistry, University of Oxford, Mansfield Road, OX1 3TA, Oxford, UK.

[2]Department of Chemistry, University College London, 20 Gordon Street, WC1H 0AJ, London UK.

*Correspondence: m.j.booth@ucl.ac.uk

Contents

Supplementary Methods 5

Oligonucleotide sequences 5

General 5

Oligonucleotides used in PCR 5

Oligonucleotides for RNase H-active antisense screening against mVenus 5

PS-containing oligonucleotides 6

Chemical Synthesis 9

General Synthesis 9

S-(4,5-Dimethoxy-2-nitrobenzyl) *O,O*-diethyl phosphorothioate S1 10

Supplementary Data 11

Screening for antisense oligonucleotides against mVenus 11

Scrambled antisense oligonucleotide control 12

Buffer screening crude chromatograms 13

LCMS of 1PSNV oligonucleotide after photocleavage 14

Comparison of T_m values for 1PS, 1PSEt and 1PSNV oligonucleotides 15

Screening for an improved, truncated ASO against mV 16

Reaction progress of unlabelled and TxRd-labelled 6PS ONs with NV-Br 18

pH-Change over 3 days under reaction conditions 18

Reaction progress over 3 days of TxRd-tagged ONs. 19

Stability of PSNV-ONs under the reaction conditions 20

Polyacrylamide electrophoresis of crude TxRd-tagged NV-oligonucleotides 21

UV-Visible absorption spectra of TxRd-tagged oligonucleotides 21

RNase H assay with the TxRd-tagged 20nt-9PSNV oligonucleotide 22

RNase H assay with the TxRd-tagged 14-nt 6PSNV oligonucleotide 23

Cell-free protein synthesis with the TxRd-tagged 20nt-9PSNV oligonucleotide 24

Cell-free protein synthesis with the TxRd-tagged 14nt-6PSNV oligonucleotide 25

TxRd-Fluorescence measurement of CFE with 20nt-6PSNV ASO 26

TxRd-Fluorescence Measurement of CFE with 20nt-9PSNV ASO 27

TxRd-Fluorescence measurement of CFE with 14nt-6PSNV ASO 28

UV-Visible Spectrum of *S*-(4,5-dimethoxy-2-nitrobenzyl) *O,O*-diethyl phosphorothioate 29

pH-Stability of TxRd-20nt-6PSNV ON 29

Nuclease stability 30

Band Profile of TxRd-tagged ONs under nuclease conditions 31

Melting temperature data 32

1PS ASO 33
1PSNV ASO 33
6PS ASO 34
6PSNV ASO 34
9PS ASO 35
9PSNV ASO 35
1PSEt ASO 36
HPLC purification of oligonucleotides 37
1PSEt 37
1PSNV 38
6PSNV 39
9PSNV 40
TxRd-20nt-6PSNV 41
TxRd-20nt-9PSNV 42
TxRd-14nt-6PSNV 43
Reaction screening oligonucleotide mass spectrometry 44
Condition 1: $NaHCO_3$ buffer chromatogram 45
Condition 1: $NaHCO_3$ buffer peak 5.18 46
Condition 1: $NaHCO_3$ buffer peak 5.40 47
Condition 1: $NaHCO_3$ buffer peak 5.5-6.5 48
Condition 2: Tris buffer chromatogram 49
Condition 2: Peak at 5.20 minutes 50
Condition 2: Peak at 5.40 minutes 51
Condition 2: Peak at 5.56 minutes 52
Condition 3: HEPES buffer chromatogram 53
Condition 3: Peak at 5.40 54
PO-only control with HEPES buffer chromatogram 55
PO-only control with HEPES buffer peak at 5.18 56
Oligonucleotide mass spectra 57
Oligonucleotide mass spectrometry 57
20nt-1PSEt 58
20nt-6PSNV 60
20nt-9PSNV 62
TxRd-20nt-6PSNV 64
TxRd-20nt-9PSNV 66
TxRd-14nt-6PSNV 68

Blank 70
NMR Spectra 71
S-(4,5-Dimethoxy-2-nitrobenzyl) *O,O*-diethyl phosphorothioate 71
^{1}H-NMR (600 Hz, $CDCl_3$) 71
^{13}C{^{1}H}-NMR (151 Hz, $CDCl_3$) 72
^{31}P-NMR (243 MHz, $CDCl_3$) 73
References 74
Uncropped and Unedited Gels 75

Supplementary Methods

Oligonucleotide sequences

General

Phosphate-only (PO) oligonucleotides (ONs) were purchased from Merck in desalted and lyophilised form and resuspended in 10 mM Tris pH 8. Phosphorothioate (PS) oligonucleotides were purchased from Merck, HPLC-purified in lyophilised form and dissolved in 10 mM potassium phosphate buffer, pH 7.4. The sequences are referenced throughout the text by their number of PS linkages in the backbone. Positions of PS linkages are indicated by a * in the sequence.

Oligonucleotides used in PCR

Index	Primer Name	Sequence (5'-3')
**1**	T7 Forward Primer	GAAATTAATACGACTCACTATAGGGTCTAG
**2**	Reverse Primer	GATATAGTTCCTCCTTTCAG

Supplementary Table 1: Primers used for generation of linear template DNA by PCR

Oligonucleotides for RNase H-active antisense screening against *mVenus*

Index	Starting Nucleotide mV mRNA	Sequence (5'-3')
**1**	1	TCCTCGCCCTTGCTCACCAT
**2**	134	GGTGGTGCAGATGAGCTTCA
**3**	140	CTTGCCGGTGGTGCAGATGA
**4**	220	TTCATGTGGTCGGGGTAGCG
**5**	240	ACTTGAAGAAGTCGTGCTGC
**6**	307	GTCTTGTAGTTGCCGTCGTC
**7**	309	GGGTCTTGTAGTTGCCGTCG
**8**	311	GCGGGTCTTGTAGTTGCCGT
**9**	453	GCTTGTCGGCGGTGATATAG
**10**	499	TCGATGTTGTGGCGGATCTT
**11**	545	GGGGGTGTTCTGCTGGTAGT

Supplementary Table 2: Antisense Oligonucleotides Screened for RNase H Activity against *mVenus*

PS-containing oligonucleotides

Entry	Number of PS Linkages	Length /nt	Name	Sequence (5'-3')
**1**	1	20	1PS	TTCATGTGGT*CGGGGTAGCG
**2**	6	20	6PS	TTC*ATG*TGG*TCG*GGG*TAG*CG
**3**	9	20	9PS	TT*CA*TG*TG*GT*CG*GG*GT*AG*CG
**4**	6	20	20nt-6PS	[TxRd]TTC*ATG*TGG*TCG*GGG*TAG*CG
**5**	9	20	20nt-9PS	[TxRd]TT*CA*TG*TG*GT*CG*GG*GT*AG*CG
**6**	6	14	14nt-6PS	[TxRd]TT*CA*TG*TG*GT*CG*GG
**7**	0	20	Reverse Complement	CGCTACCCCGACCACATGAA

Supplementary Table 3: Phosphorothioate (PS)-containing oligonucleotides used in this study. [TxRd] indicates Texas Red dye.

Truncated antisense oligonucleotides

**Entry**	**mV Position**	**Truncation from original ASO**	**Length/nt**	**Sequence (5'-3')**
**1**	224	3' truncated	16	TTCATGTGGTCGGGGT
**2**	220	5' Truncated	16	TGTGGTCGGGGTAGCG
**3**	222	5'/3' truncated	16	CATGTGGTCGGGGTAG
**4**	67	N/A	14	TTGTGGCCGTTTAC
**5**	220	5' truncated	14	TGGTCGGGGTAGCG
**6**	223	5'/3' truncated	14	ATGTGGTCGGGGTA
**7**	226	3' truncated	14	TTCATGTGGTCGGG

Supplementary Table 4: Truncated mV antisense oligonucleotides screened for improvement of antisense activity in cell-free conditions.

Scrambled antisense sequences

**Entry**	**Name**	**Sequence (5'-3')**
**1**	SCR1	AGGTGTGTCGTGCATGGTGC
**2**	SCR2	GGAGCGGTCGGTATCGTTGT
**3**	SCR3	TGGTGGCAGCCGTGTTGAGT
**4**	SCR4	GGATGTGCGGGCTTTCGGAT

Supplementary Table 5: Scrambled sequences of mV antisense sequence 220 (**Supplementary Table 2**, Entry 4)

DNA sequences of genes used

Entry	Gene	Sequence 5’-3’
**1**	*mVenus*	GAAATTAATACGACTCACTATAGGGTCTAGAAATAATTTTGTTTAACTTTAAGAA<br>GGAGGTATACAT**ATG**GTGAGCAAGGGCGAGGAGCTGTTCACCGGGGTGGTGC<br>CCATCCTGGTCGAGCTGGACGGCGACGTAAACGGCCACAAGTTCAGCGTGTCC<br>GGCGAGGGCGAGGGCGATGCCACCTACGGCAAGCTGACCCTGAAGCTCATCTG<br>CACCACCGGCAAGCTGCCCGTGCCCTGGCCCACCCTCGTGACCACCCTCGGCTA<br>CGGCCTGCAGTGCTTCGCCCGCTACCCCGACCACATGAAGCAGCACGACTTCTT<br>CAAGTCCGCCATGCCCGAAGGCTACGTCCAGGAGCGCACCATCTTCTTCAAGGA<br>CGACGGCAACTACAAGACCCGCGCCGAGGTGAAGTTCGAGGGCGACACCCTG<br>GTGAACCGCATCGAGCTGAAGGGCATCGACTTCAAGGAGGACGGCAACATCCT<br>GGGGCACAAGCTGGAGTACAACTACAACAGCCACAACGTCTATATCACCGCCG<br>ACAAGCAGAAGAACGGCATCAAGGCCAACTTCAAGATCCGCCACAACATCGAG<br>GACGGCGGCGTGCAGCTCGCCGACCACTACCAGCAGAACACCCCCATCGGCGA<br>CGGCCCCGTGCTGCTGCCCGACAACCACTACCTGAGCTACCAGTCCAAGCTGAG<br>CAAAGACCCCAACGAGAAGCGCGATCACATGGTCCTGCTGGAGTTCGTGACCG<br>CCGCCGGGATCACTCTCGGCATGGACGAGCTGTACAAG**TAA**TGAGGATCCCGG<br>GAATTCTCGAGTAAGGTTAACCTGCAGGAGGCCTTTAATTAAGGTGGTGCGGC<br>CGCGCTAGCGGTCCCGGGGGATCGATCCGGCTGCTAACAAAGCCCGAAAGGAA<br>GCTGAGTTGGCTGCTGCCACCGCTGAGCAATAACTAGCATAACCCCTTGGGGCC<br>TCTAAACGGGTCTTGAGGGGTTTTTTGCTGAAAGGAGGAACTATATC

Supplementary Table 6: Sequences of linear DNA templates used for cell-free expression, produced by PCR. T7-Promoter and -Terminator Regions are underlined, protein start- and stop-codons are in bold. mRNA position counting starts at the bolded start codon ATG.

Chemical Synthesis

General Synthesis

Reagents were purchased from commercial sources (Merck) and used without further purification. Solvents were used as supplied (analytical/HPLC-grade from Fisher or Merck) or if dry solvents were required, taken from a solvent drying system (MBraun MB-SPS-5-Bench Top) under nitrogen atmosphere (H_2O content < 20 ppm as determined by Karl Fischer titration). Petroleum ether (PE) over a boiling point range of 40–60 °C was used. Eluent mixtures are reported in volume:volume or %vol. Column chromatography was carried out using Merck Geduran Silica Gel 60 or VWR Silica Gel 40-63 µm under N_2 pressure. TLC was carried out on Merck silica gel 60 F254 Al plates. NMR spectroscopy measurements were recorded using a Bruker AVIII600 instrument and peaks were referenced to the residual solvent peak. Mass Spectrometry (MS) measurements were carried out on Waters Bioaccord MS system**.** Infrared Spectra (IR) were recorded on a Bruker Tensor 27 FT-IR and classified as strong (s), medium (m), weak (w) and broad (b). UV/Visible light absorption measurements were performed on a Carys UVBio 50 Spectrometer in 1.4 mL quartz cuvettes (ThorLabs).

***S*-(4,5-Dimethoxy-2-nitrobenzyl) *O,O*-diethyl phosphorothioate S1**

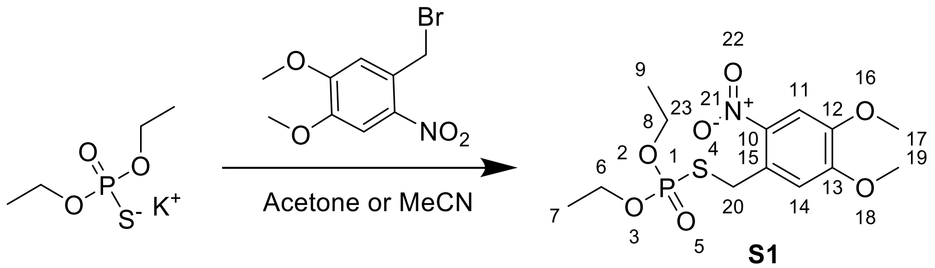

To a scintillation vial equipped with a stirrer bar, *O,O*-diethyl thiophosphate potassium salt (100 mg, 0.480 mmol) and 2-nitroveratryl bromide (133 mg, 0.480 mmol) were added. To this, either dry acetone or acetonitrile (2 mL) were added and the reaction stirred for 2 hours at room temperature. The resulting suspension was diluted with 10 mL of H_2O and the aqueous layer extracted with EtOAc (2x 20 mL), the organic layer dried ($MgSO_4$), filtered and reduced *in vacuo*. The resulting crude oil was then purified by flash column chromatography (1:1 to 2:1 PE:EtOAc) to yield a viscous, yellow oil as the desired compound (using acetonitrile as solvent: 161.3 mg, 0.441 mmol, 92% yield. Using acetone as solvent: 168.6 mol, 0.461 mmol, 96% yield). **R_f** (1:1 PE:EtOAc) 0.17. **^{1}H-NMR** (600 MHz, $CDCl_3$) δ/ppm 7.69 (s, 1H, 11), 7.13 (s, 1H, 14), 4.39 (d, *J* = 15.9 Hz, 2H, 20), 4.14 – 3.95 (m, 4H, 6, 8), 3.99 (s, 3H, 19), 3.95 (s, 3H, 17), 1.27 (td, *J* = 7.1, 0.8 Hz, 6H, 7, 9). **^{13}C-NMR** (151 MHz, $CDCl_3$) δ/ppm 153.4 (13), 148.5 (12), 140.1 (10), 129.0 (d, *J* = 2.9 Hz, 15), 114.2 (14), 108.5 (11), 63.9 (d, *J* = 6.1 Hz, 6, 8), 56.8 (19), 56.6 (17), 33.3 (d, *J* = 3.6 Hz, 20), 16.1 (d, *J* = 7.1 Hz, 7, 9). **^{31}P-NMR** (243 MHz, $CDCl_3$) δ/ppm 27.21 (tp, *J* = 17.1, 8.8 Hz, 1). **HRMS** (ESI$^+$) found 388.0605, $[C_{13}H_{20}NO_7PSNa]^+$ requires 388.0590 (Error 3.76 ppm). **IR** (neat, cm^{-1})**:** 1523 (s, NO_2), 1393 (s, NO_2), 1275 (s, P=O), 1232 (m), 1063 (s), 1015 (s), 972 (m), 797 (m, Aromatic C-H). **UV/Vis** (25% DMSO/H_2O) λ_{max}/nm (ε/mM^{-1} cm^{-1}): 352 (5.3), 313 (4.4).

Supplementary Data

Screening for antisense oligonucleotides against *mVenus*

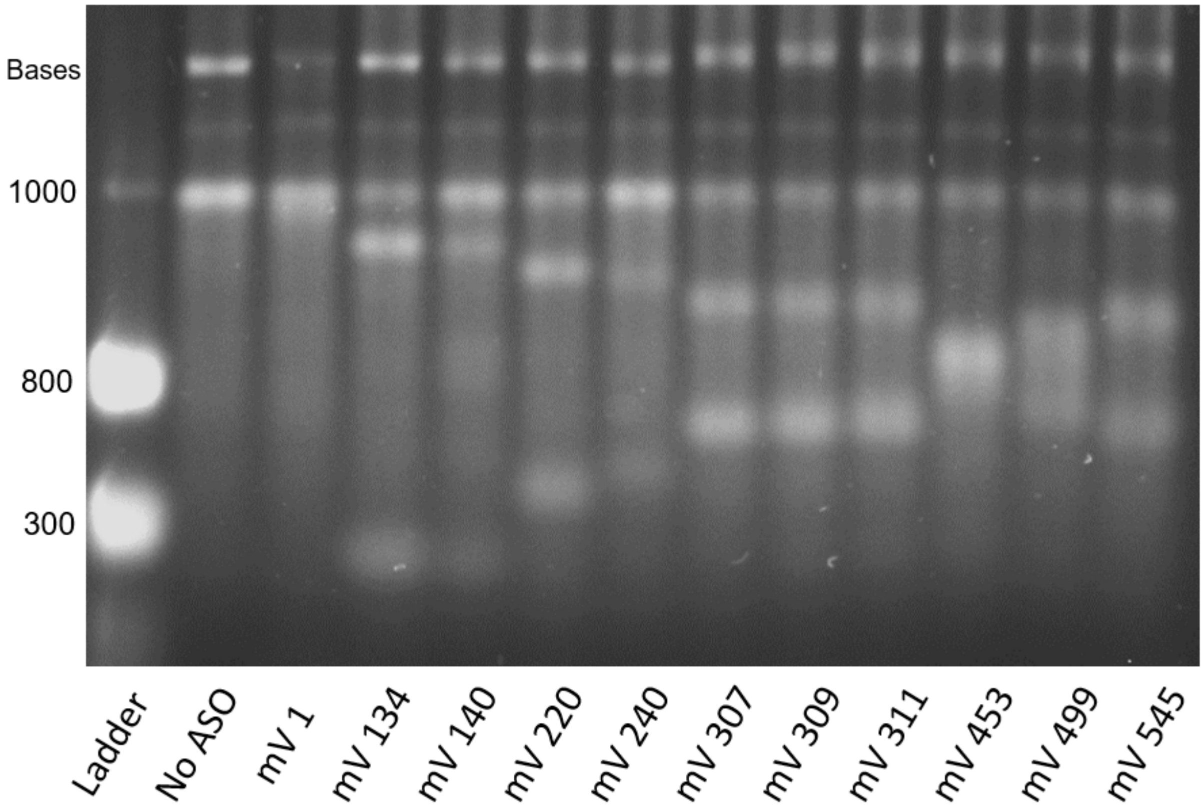

Supplementary Figure 1: Agarose gel of ASO screen. ASOs (**Supplementary Table 2**, number indicates stating nucleotide in the *mVenus* sequence) were incubated with target *mVenus* mRNA (~1000 bases in length) in the presence of RNase H for 1h prior to analysis by agarose gel. Uncropped and unedited gel image found as **Supplementary Figure 32**.

Scrambled antisense oligonucleotide control

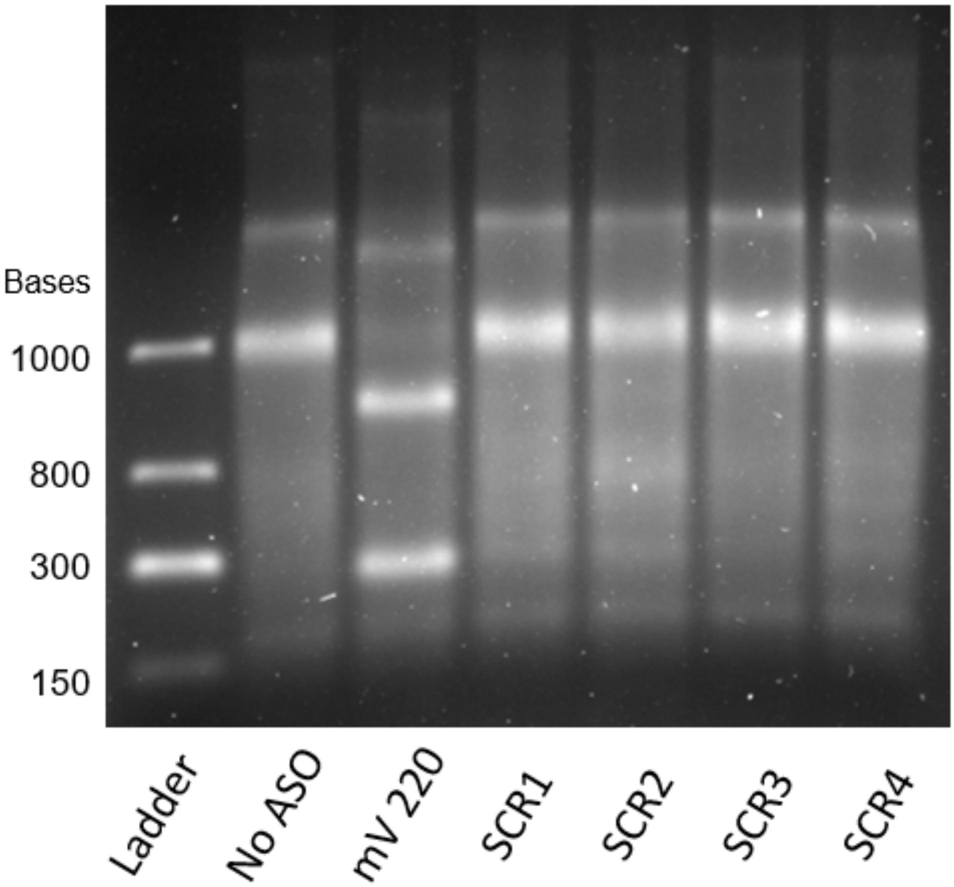

Supplementary Figure 2: Scrambled oligonucleotide sequences (**Supplementary Table 5**) tested against *mVenus* mRNA to check for specificity of the sequence for its target. Uncropped and unedited gel image found as **Supplementary Figure 33**.

Buffer screening crude chromatograms

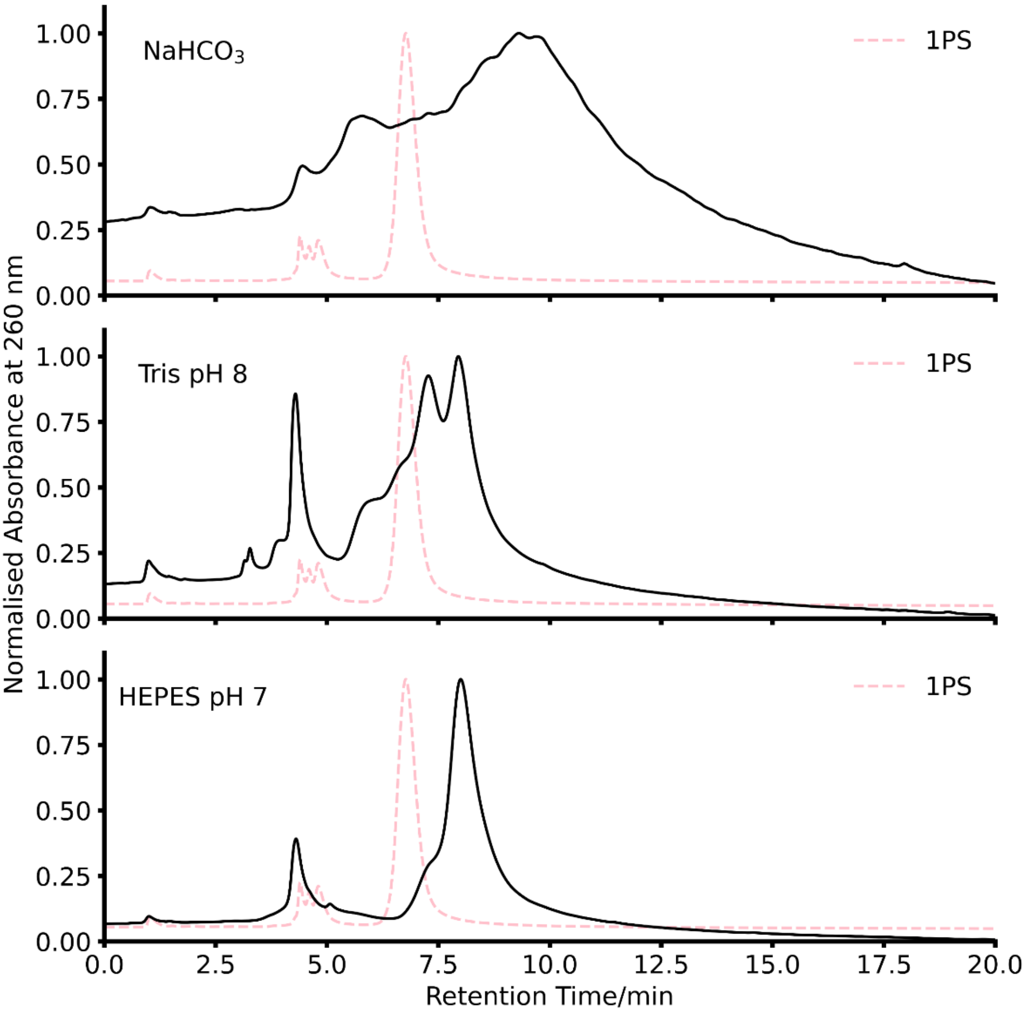

Supplementary Figure 3: Buffer screening for nitroveratryl-modification of the 1PS-containing oligonucleotide (**Supplementary Table 3, Entry 1**). Crude reaction mixtures were analysed by HPLC after ethanol precipitation. HEPES pH 7 was the only buffer to give appreciable and clean conversion to the desired oligonucleotide.

LCMS of 1PSNV oligonucleotide after photocleavage

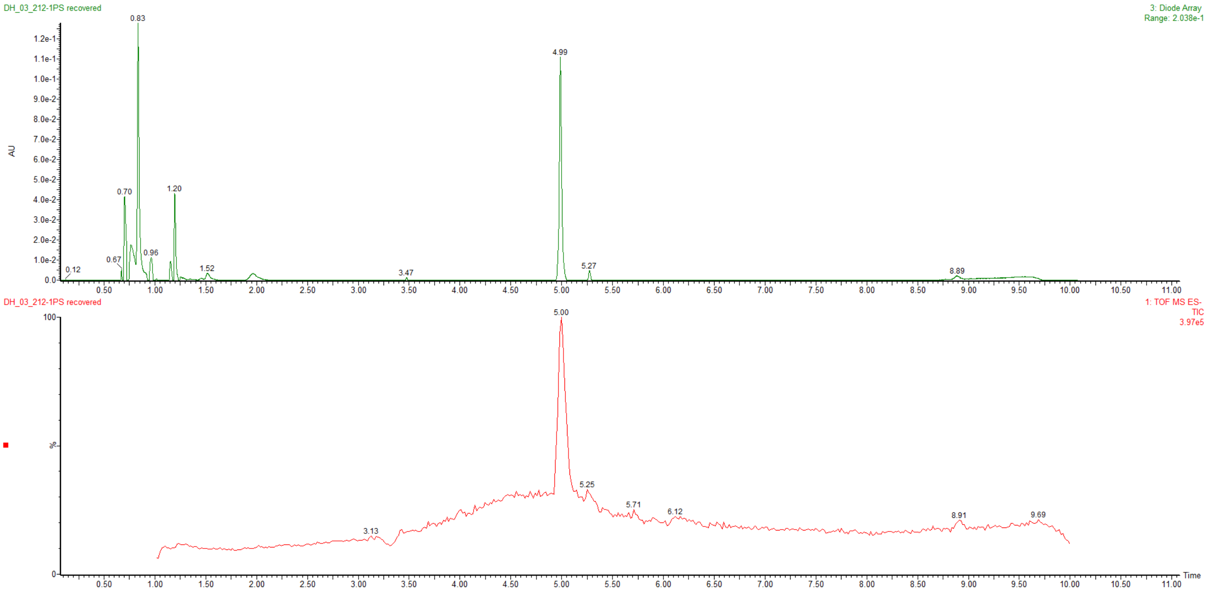

Supplementary Figure 4: LCMS chromatogram of photocleavage product from **Figure 2c.**

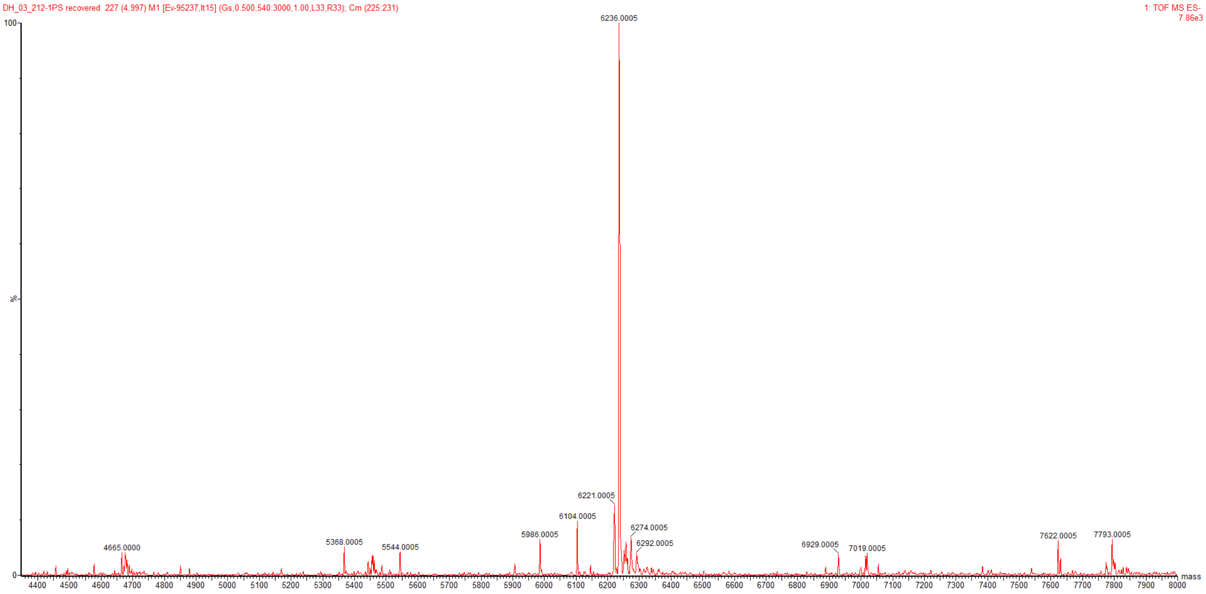

Supplementary Figure 5: Mass of photocleavage product from **Figure 2c**. The observed mass of 6236 corresponds to the original PS-containing oligonucleotide (Expected Mass 6236).

Comparison of T_m values for 1PS, 1PSEt and 1PSNV oligonucleotides

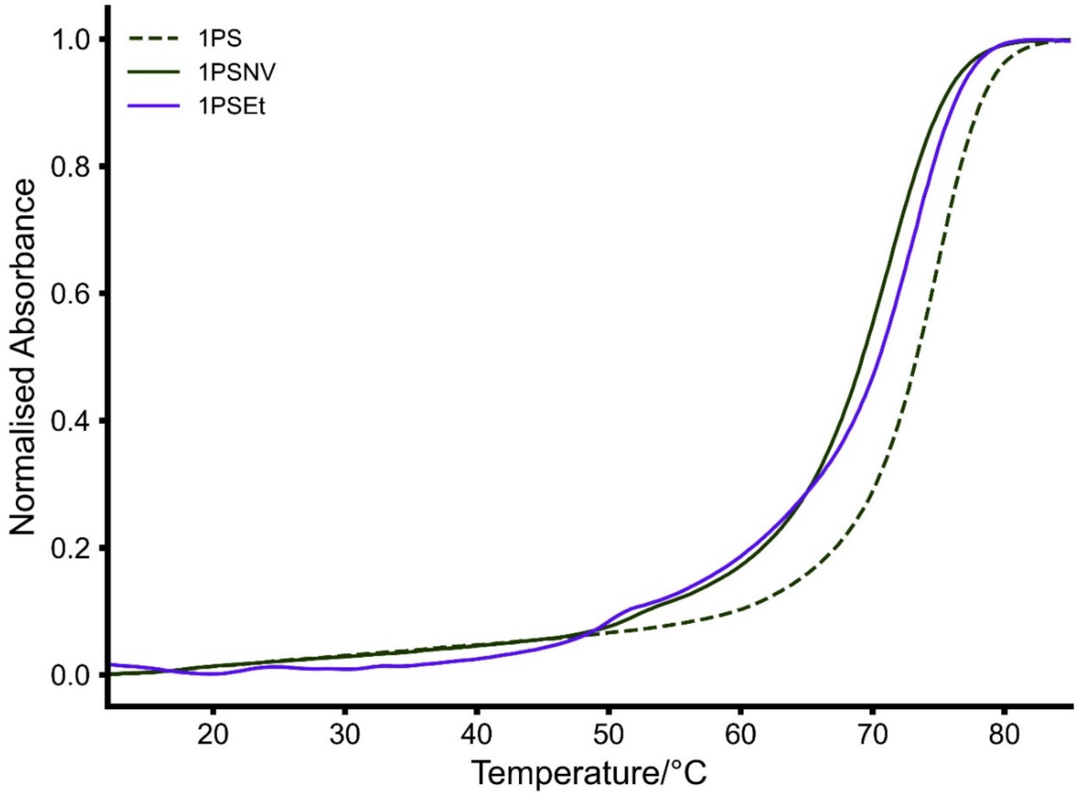

Supplementary Figure 6: T_m measurements of 1PS, 1PSNV and 1PSEt oligonucleotides. The ethyl-modification shows a slightly lower reduction in T_m than the NV modification.

Screening for an improved, truncated ASO against mV

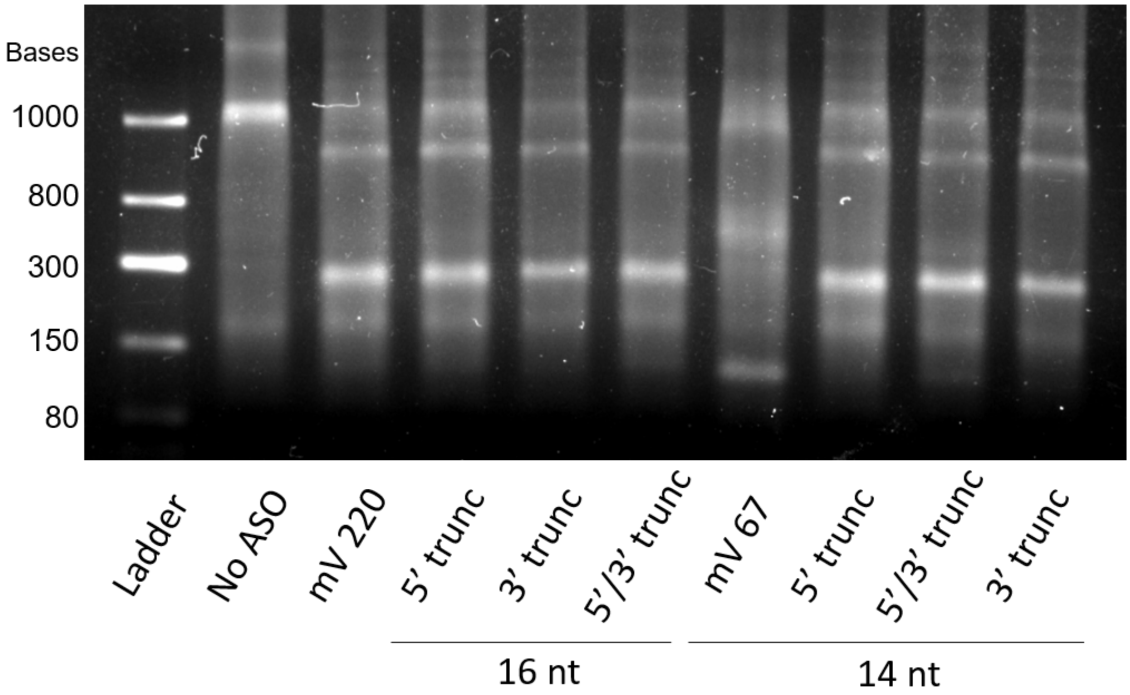

Supplementary Figure 7: Agarose gel of RNase H-degradation of *mVenus* mRNA with truncated ASOs based on mV 220 (**Supplementary Table 4**), ran against a ssRNA ladder. Note: 2 ng of ASOs were used per condition, which for the 16 nt-long ASOs equates to a 1.2-fold molar excess, and for the 14 nt-long sequences a 1.4-fold molar excess compared to the 20nt-sequence. Uncropped and unedited gel image found as **Supplementary Figure 34**.

**Entry**	**Lane**	**Ratio**
**1**	L1	N/A (Ladder)
**2**	L2	0 (Reference)
**3**	L3	0.192
**4**	L4	0.477
**5**	L5	0.187
**6**	L6	0.279
**7**	L7	Not Determinable
**8**	L8	0.316
**9**	L9	0.236
**10**	L10	0.164

Supplementary Table 7: Ratios of *mVenus* mRNA degradation bands to full length product from **Supplementary Figure 7**. Degradation was calculated using the ratio of full-length band to shortest band from intensity data using ImageLab.

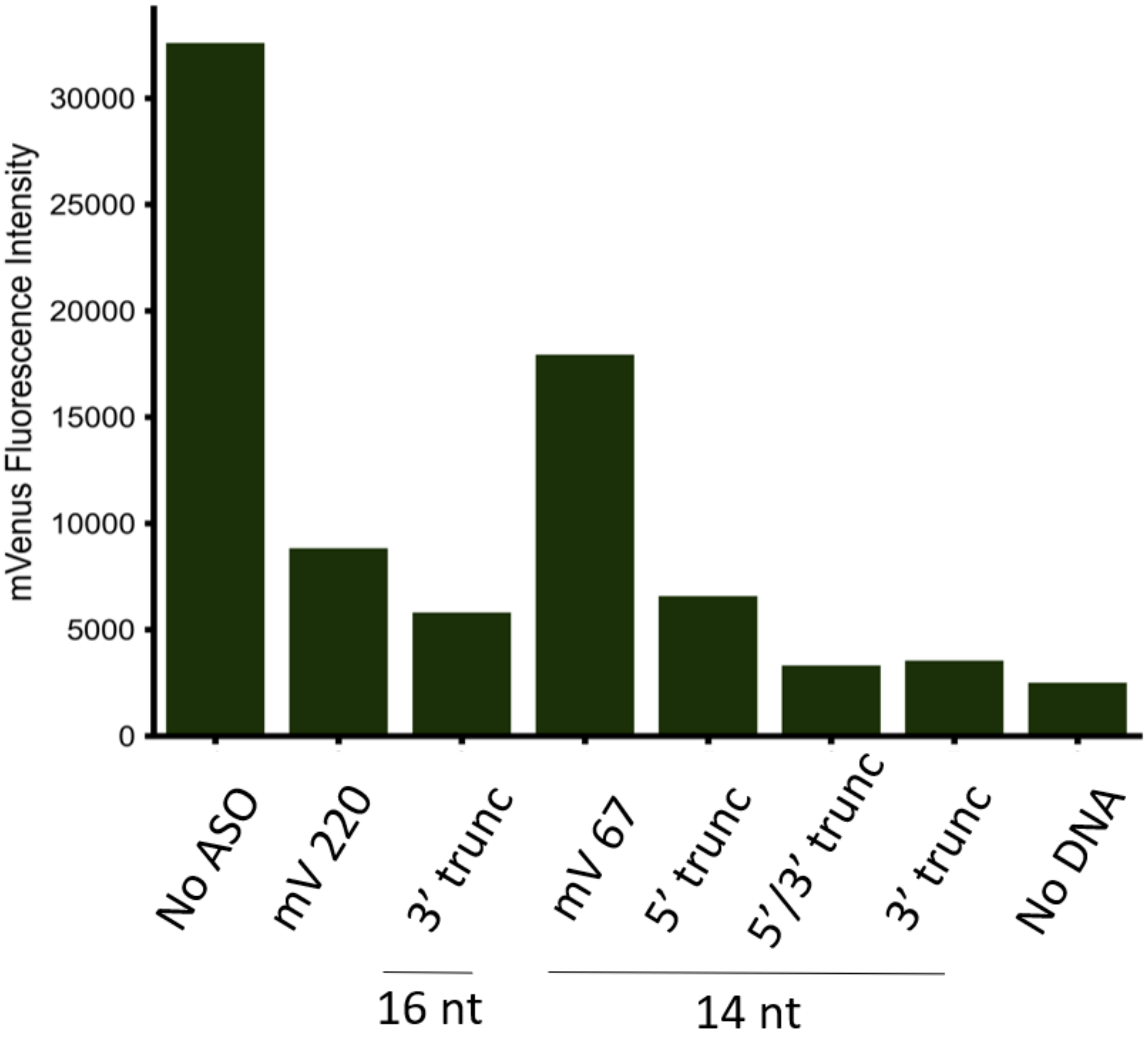

Supplementary Figure 8: Gene knockdown of cell-free protein synthesis of mVenus using the truncated ASO sequences and RNase H. 500 pg of each ASO was used, which for the 16 nt-long ASOs equates to a ~1.2-fold molar excess and for the 14 nt-long sequences a ~1.4-fold molar excess, vs. the 20nt-long ASO. n=1

Reaction progress of unlabelled and TxRd-labelled 6PS ONs with NV-Br

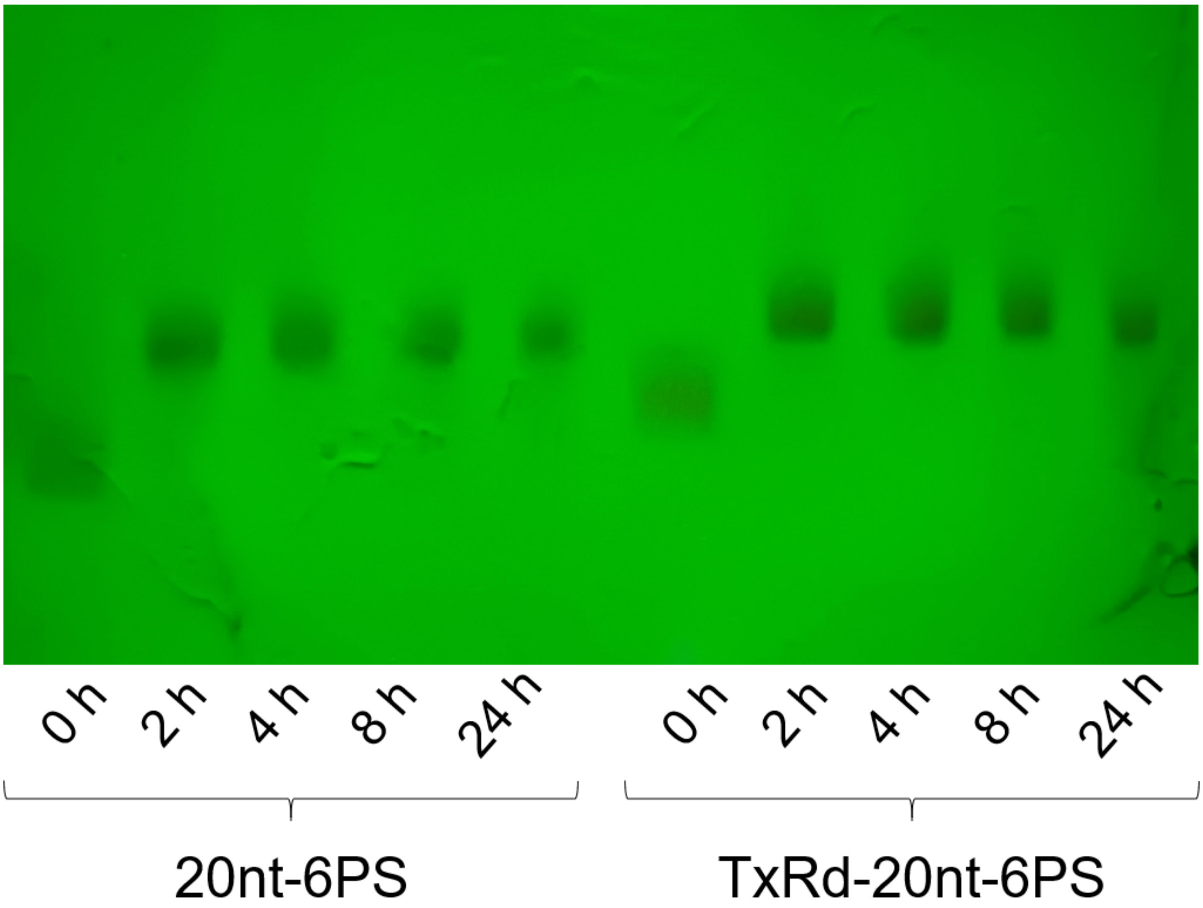

Supplementary Figure 9: Alkylation reaction progress at different timepoints as analysed by AGE (HAE Buffer) and visualised by illumination over a TLC plate. The reaction was complete after 2 hours. Further incubation up to 24 hours seemed to have no detrimental effect. Uncropped and unedited gel image found as **Supplementary Figure 35**.

pH-Change over 3 days under reaction conditions

Entry	HEPES Buffer Concentration/mM	pH at Day 0	pH at Day 3
**1**	100	6.86	5.65
**2**	500	6.96	6.84

Supplementary Table 8: pH changes over 3 days under the reaction conditions used for the synthesis of PSNV ONs as well as at higher buffer concentration. The starting HEPES buffer at 1 M had a pH of 6.89.

Reaction progress over 3 days of TxRd-tagged ONs.

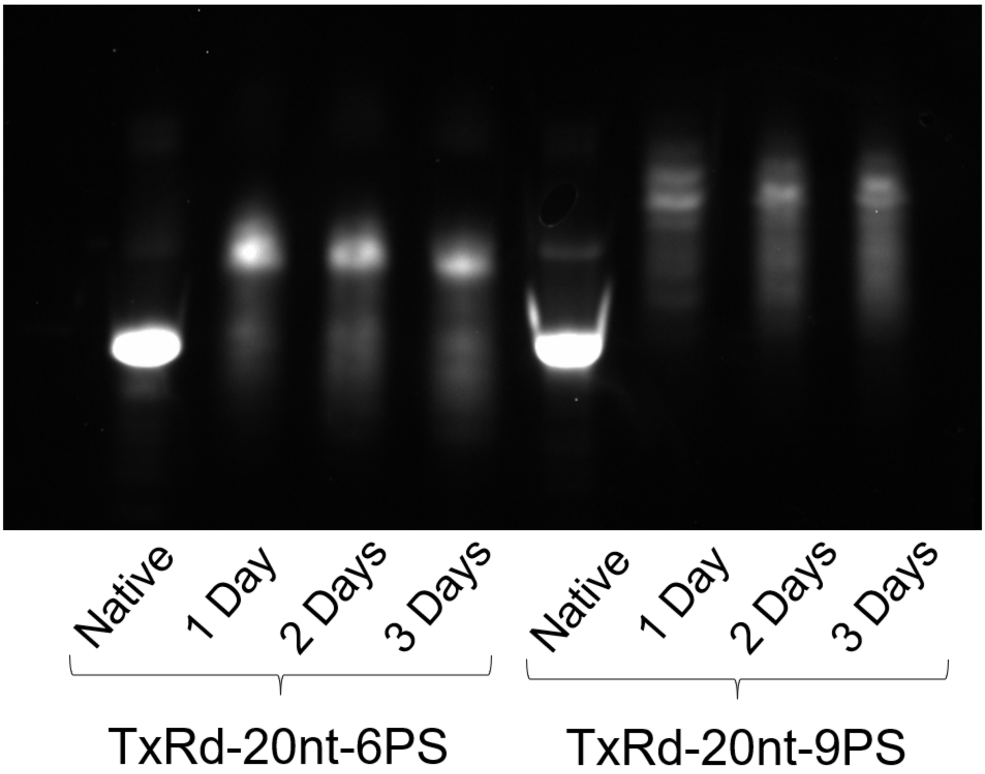

Supplementary Figure 10: TxRd-tagged 20nt-ONs under prolonged reaction conditions and analysed by PAGE using HAE buffer. Despite acidification during the reaction, no appreciable amounts of degradation were observed over 3 days. Uncropped and unedited gel image found as **Supplementary Figure 36**.

Stability of PSNV-ONs under the reaction conditions

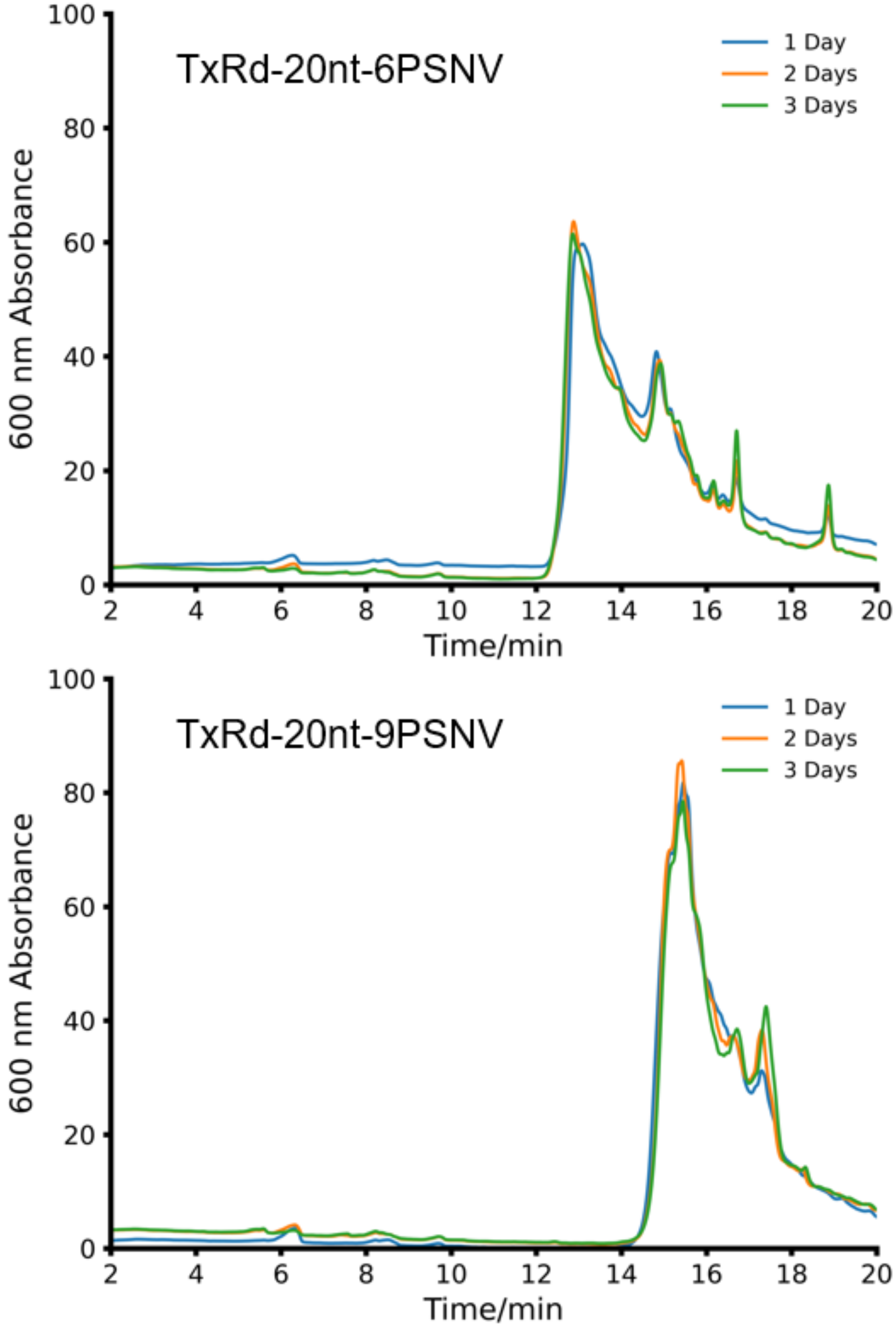

Supplementary Figure 11: HPLC traces of crude reaction mixture monitored at 600 nm over 3 days. 50 µL of the crude reaction mixture was analysed after 24, 28 and 72 hours. The yield of the desired ON did not decrease over this time period, showing stability of the ON under extended reaction conditions.

Polyacrylamide electrophoresis of crude TxRd-tagged NV-oligonucleotides

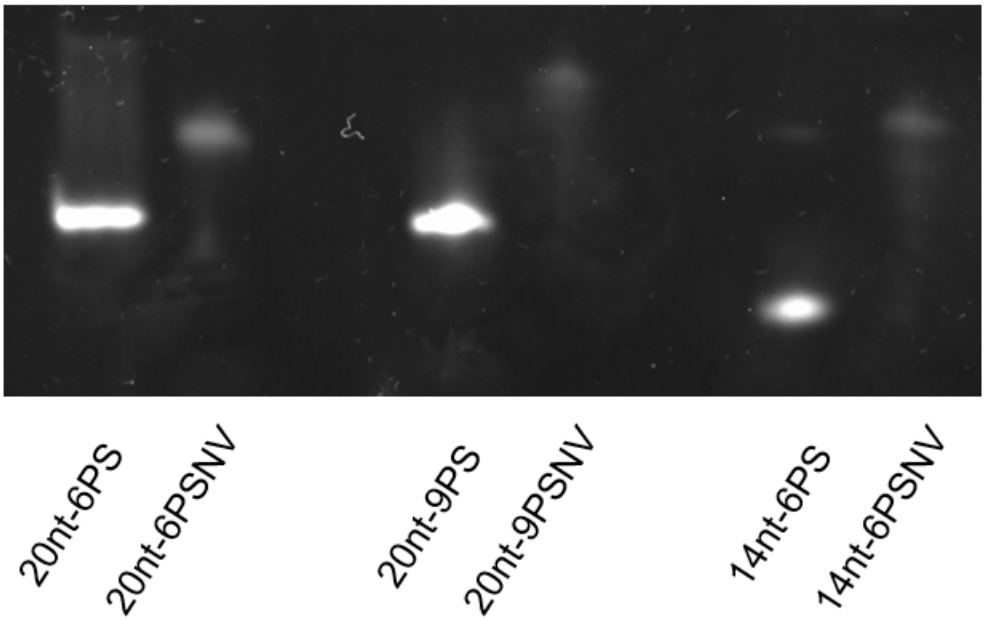

Supplementary Figure 12: Polyacrylamide Gel Electrophoresis of crude reaction products of the TexasRed-tagged ONs post-modification with nitroveratryl groups compared against the unmodified ONs. Modification with NV-groups causes a large gel retention due to increased size and reduced charge of the resulting ON. Uncropped and unedited gel image found as **Supplementary Figure 37**.

UV-Visible absorption spectra of TxRd-tagged oligonucleotides

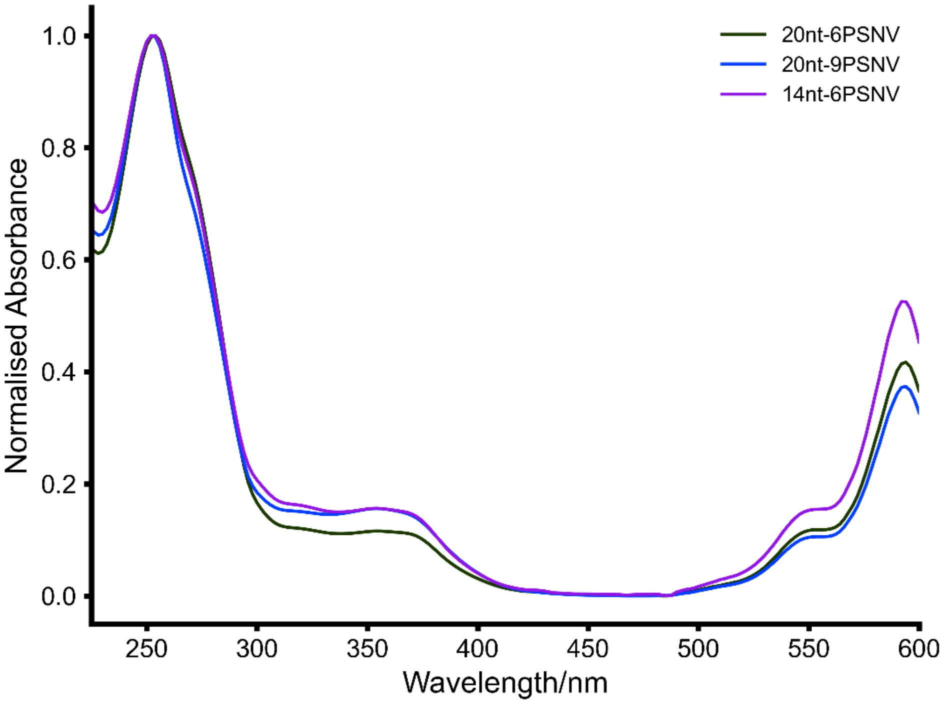

Supplementary Figure 13: Absorbance spectra of Texas-Red tagged, nitroveratryl-modified oligonucleotides recorded by HPLC from 230 to 600 nm, normalised to the peak at 260 nm.

RNase H assay with the TxRd-tagged 20nt-9PSNV oligonucleotide

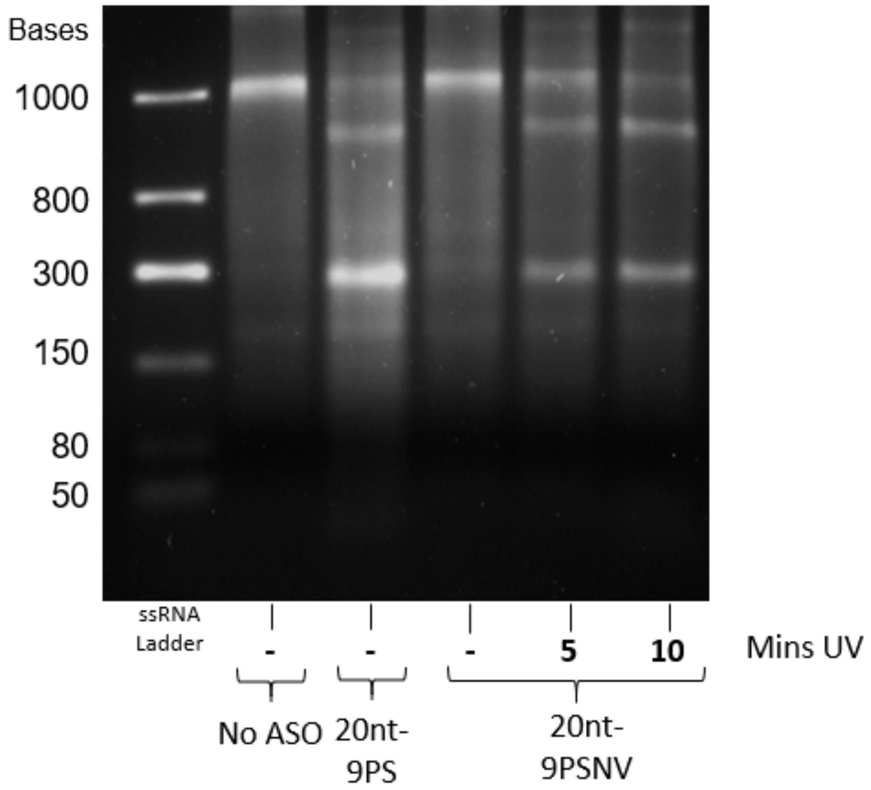

Supplementary Figure 14: RNase H activity of 20nt-9PSNV ON with and without light. In the absence of irradiation, the modified oligonucleotide shows no RNase H activity against its target mRNA, but upon illumination with UV light, activity could be restored. Higher activity was observed with more UV irradiation. Uncropped and unedited gel image found as **Supplementary Figure 39**.

RNase H assay with the TxRd-tagged 14-nt 6PSNV oligonucleotide

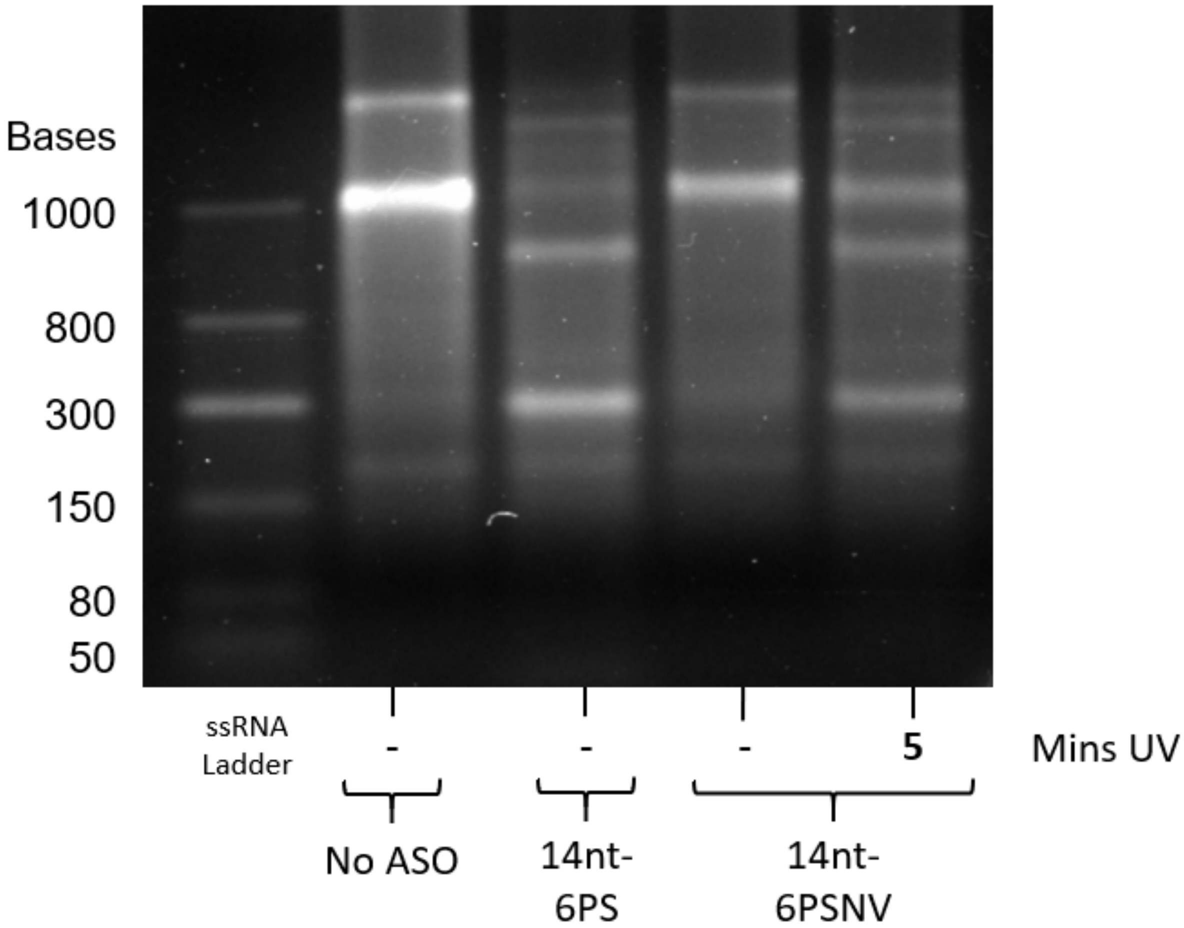

Supplementary Figure 15: RNase H activity of 14-nt-6PSNV ON with and without light. In the absence of irradiation, the modified oligonucleotide shows no RNase H activity against its target mRNA, but upon illumination with UV light, activity could be restored. Uncropped and unedited gel image found as **Supplementary Figure 40**.

Cell-free protein synthesis with the TxRd-tagged 20nt-9PSNV oligonucleotide

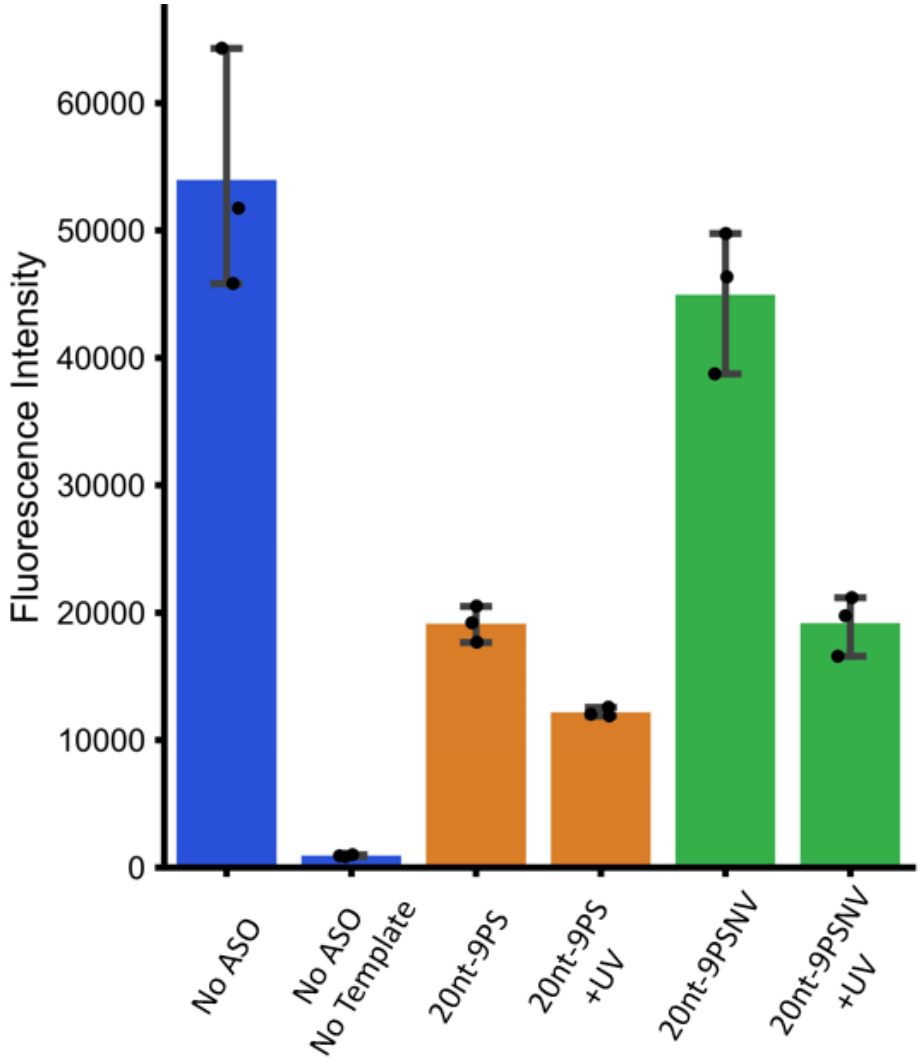

Supplementary Figure 16: Cell-free protein synthesis with added RNase H of mVenus in presence of 20nt-9PS and -9PSNV ON with and without light. Illumination of the unmodified ASO for 10 minutes shows UV damage to the system. In the absence of irradiation, the 9PSNV ON shows small amounts of mVenus knockdown of 17%, but upon illumination with UV light, activity could be restored to levels seen with the unmodified ON prior to illumination, corresponding to 83% of the activity of the illuminated, unmodified ASO. Error bars show 95% confidence interval (n=3).

Cell-free protein synthesis with the TxRd-tagged 14nt-6PSNV oligonucleotide

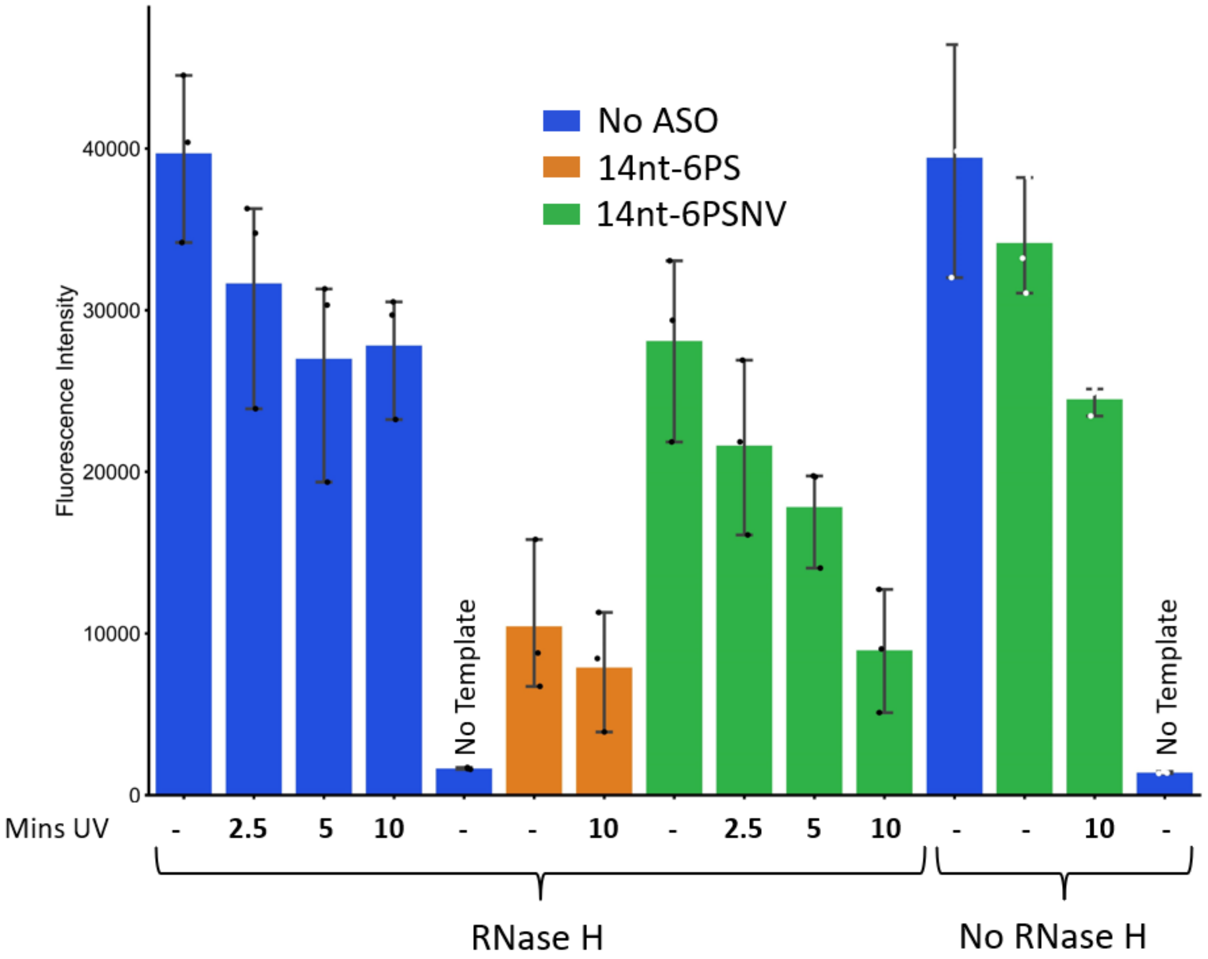

Supplementary Figure 17: Cell-free protein synthesis with added RNase H of mVenus in presence of 14nt-6PS and -6PSNV ON with and without light. Illumination of the system shows UV damage, seen with and without ASO. In the absence of irradiation, the 14nt-6PSNV ON shows some mVenus knockdown of 30% and upon illumination with UV light, activity could be gradually restored to levels seen with the unmodified ON by application of up to 10 minutes of UV-light, leading to 97% of the activity of the illuminated, unmodified ASO. Error bars show 95% confidence interval (n=3).

TxRd-Fluorescence measurement of CFE with 20nt-6PSNV ASO

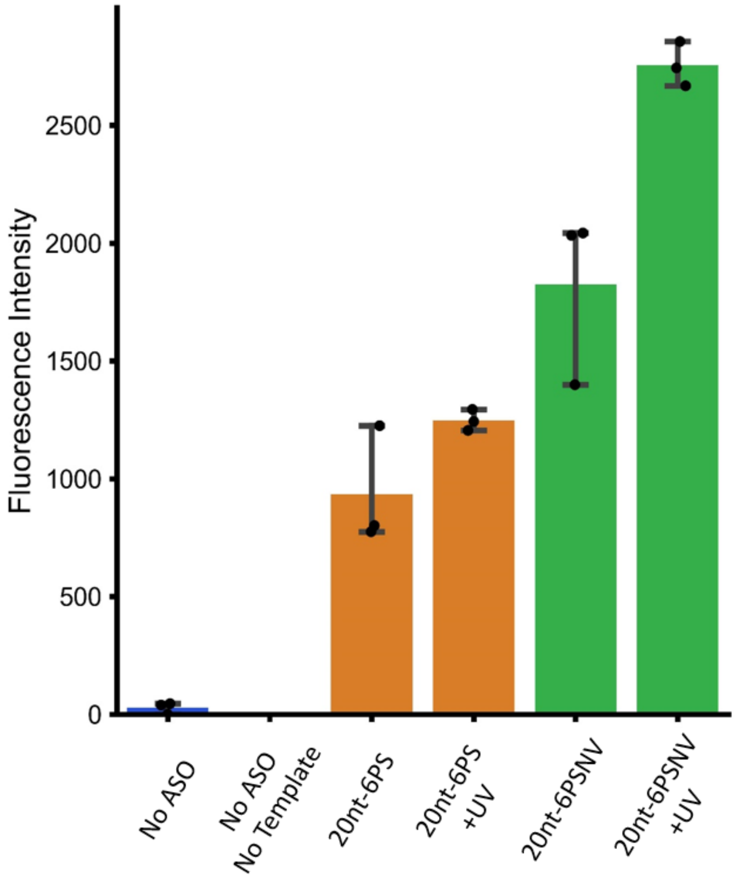

Supplementary Figure 18: Texas-Red fluorescence within cell-free protein synthesis reactions using the 20nt-6PSNV ON. The modified ON shows increased fluorescence after photocleavage. $\lambda_{Ex/Em}$: 596/615 nm, Gain: 200. Error bars show 95% confidence interval (n=3).

TxRd-Fluorescence Measurement of CFE with 20nt-9PSNV ASO

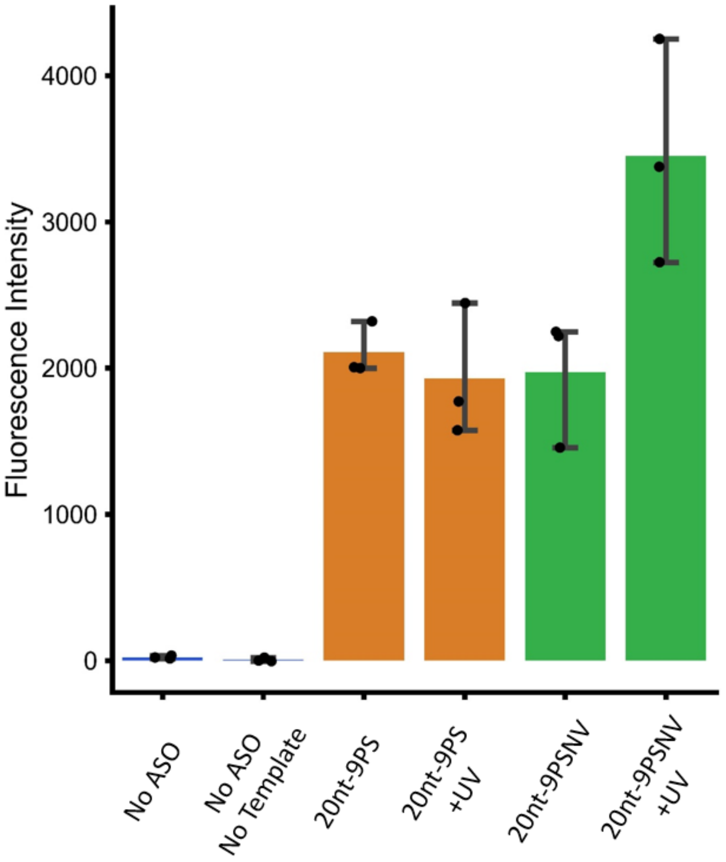

Supplementary Figure 19: Texas-Red fluorescence within cell-free protein synthesis reactions using the 20nt-9PSNV ON. The modified ON shows increased fluorescence after photocleavage. $\lambda_{Ex/Em}$: 596/615 nm, Gain: 200. Error bars show 95% confidence interval (n=3).

TxRd-Fluorescence measurement of CFE with 14nt-6PSNV ASO

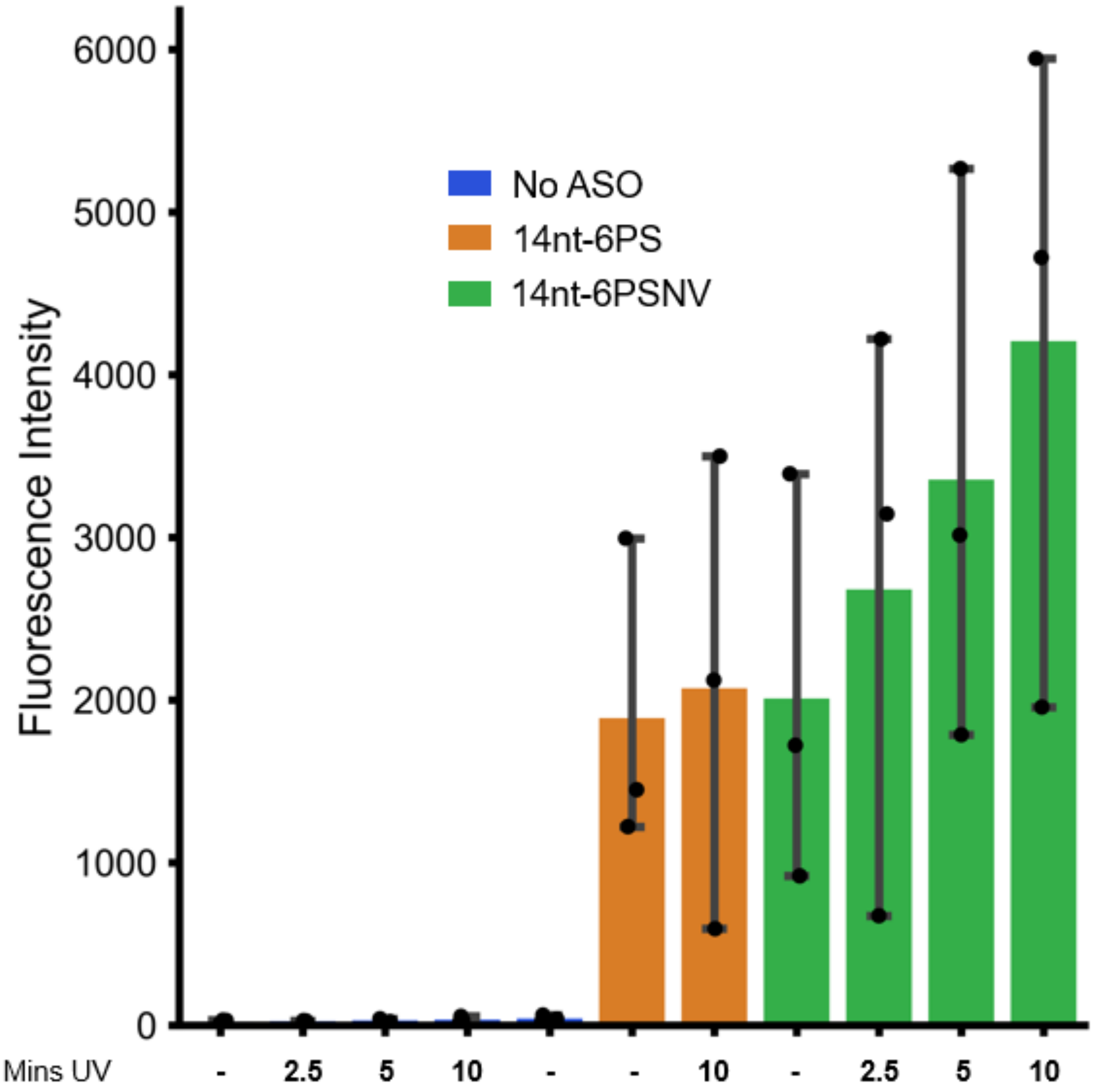

Supplementary Figure 20: Texas-Red fluorescence within cell-free protein synthesis reactions using the 14nt-6PSNV ON in presence of RNase H. The modified ON shows increased fluorescence with progressive photocleavage. $\lambda_{Ex/Em}$: 596/615 nm, Gain: 200. Error bars show 95% confidence interval (n=3).

UV-Visible Spectrum of *S*-(4,5-dimethoxy-2-nitrobenzyl) *O,O*-diethyl phosphorothioate

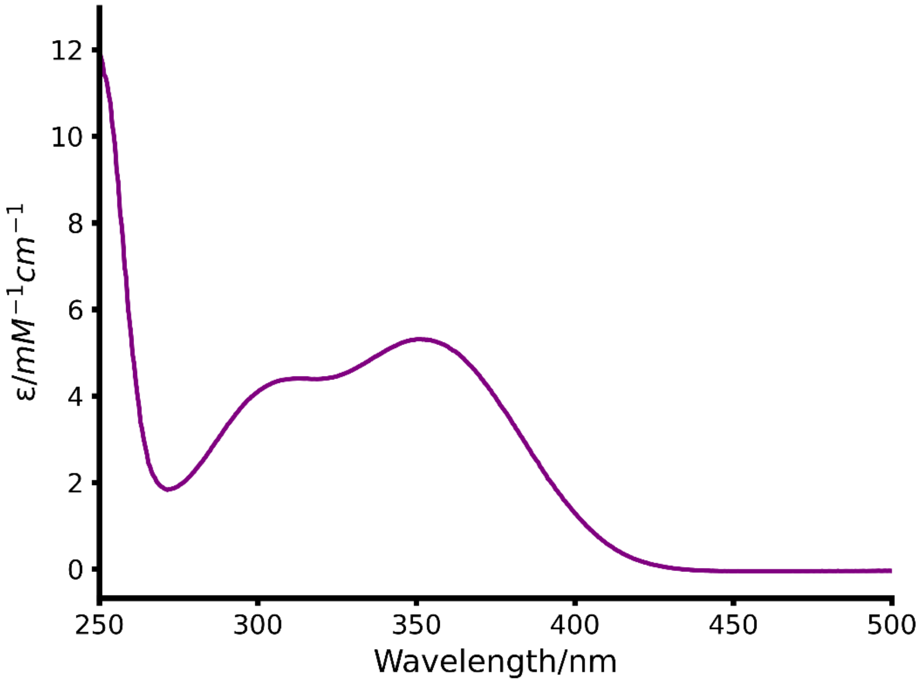

Supplementary Figure 21: UV-visible absorbance spectrum of **S1** in 25% DMSO in H_2O.

pH-Stability of TxRd-20nt-6PSNV ON

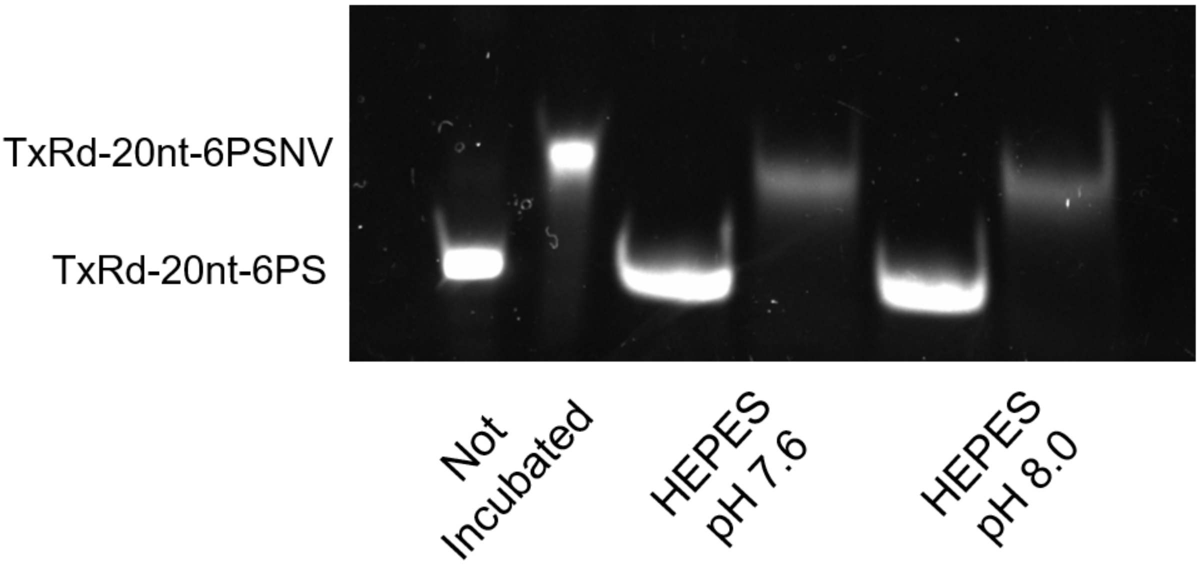

Supplementary Figure 22: pH-stability of purified TxRd-20nt-6PSNV and the native -6PS ONs. Both ONs were incubated in 50 mM HEPES buffer at either pH 7.6 or pH 8.0 for 4 hours at 37 °C before analysis by polyacrylamide gel using HAE buffer. No visible degradation was observed for either ON under both buffer conditions. Uncropped and unedited gel image found as **Supplementary Figure 41**.

Nuclease stability

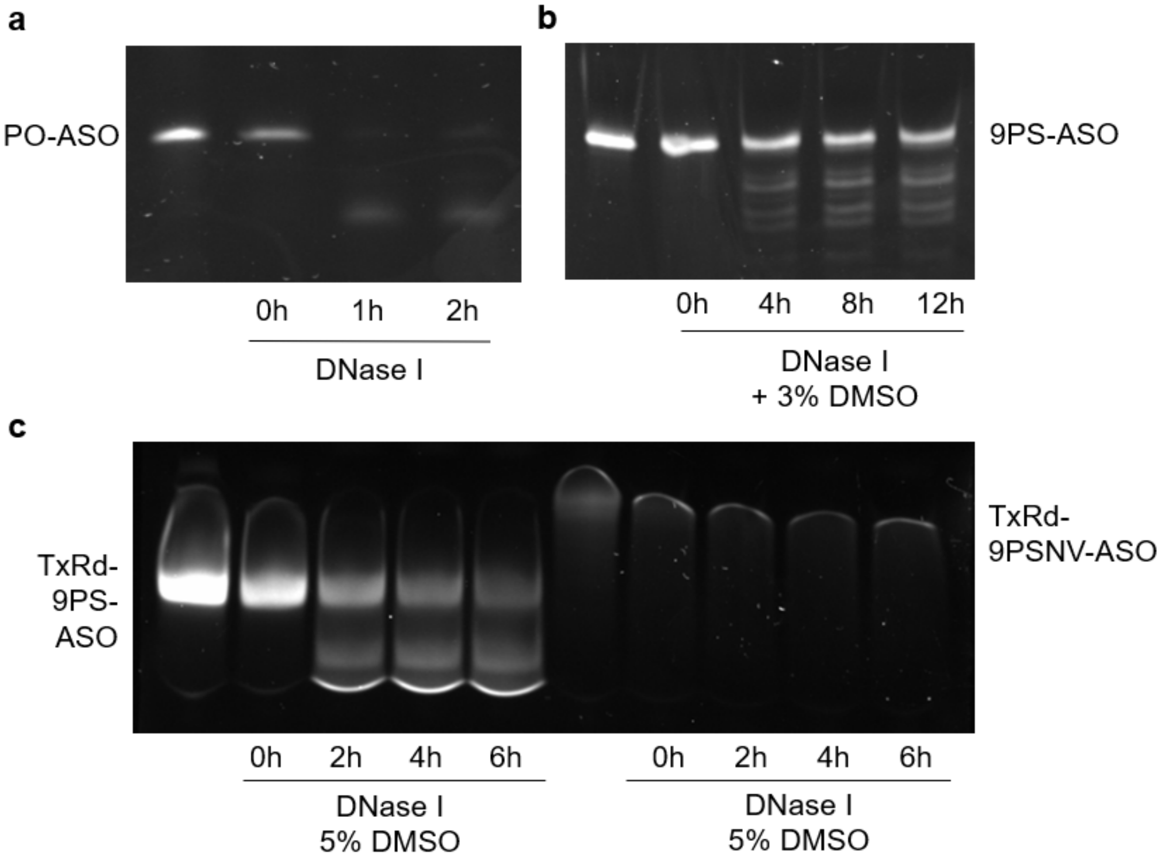

Supplementary Figure 23: Stability of 20nt-ASOs against DNase I. **a**) Incubation of phosphate-only ON with DNAse I shows rapid degradation of the ON. **b**) Incubation of the 9PS ON shows drastically improved stability compared to the PO only ON. **c**) Comparison of TxRd-9PS ON pre- and post-modification with 2-nitroveratryl bromide. The nitroveratryl groups slow degradation of the ON by DNase I. Uncropped and unedited gel images found as **Supplementary Figures 42-44**.

Band Profile of TxRd-tagged ONs under nuclease conditions

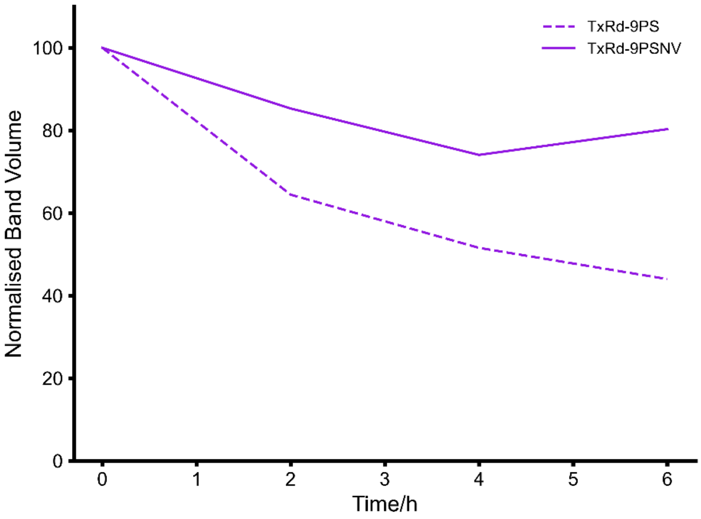

Supplementary Figure 24: Normalised DNA band volumes of TxRd-tagged 9PS and 9PSNV conditions with DNase I treatment (**Supplementary Figure 23**, c). Band volumes were normalised to the T=0h condition with DNase I. Faster reduction in band volume is observed for the unmodified ON compared to the NV-modified ON, showing reduced degradation upon modification with 2-nitroveratryl bromide.

Melting temperature data

Melting Temperatures were recorded on a Jasco V770 Spectrophotometer equipped with a Peltier Station. Green lines indicate heating cycles, whereas blue lines indicate cooling cycles. Data was smoothed using a Savitzky-Golay filter. Values were calculated as an average of 3 heating/cooling cycles.

**Entry**	**Oligo**	**T_m /°C**	**ΔT_m /°C**
**1**	1PS	72.15	
**2**	1PSNV	68.44	-3.71
**3**	6PS	71.79	
**4**	6PSNV	48.76	-23.03
**5**	9PS	66.91	
**6**	9PSNV	33.78	-33.13
**7**	1PSEt	70.03	-2.12

Supplementary Table 9: Melting Temperatures (T_m) and ΔT_m of the modified oligonucleotides.

1PS ASO

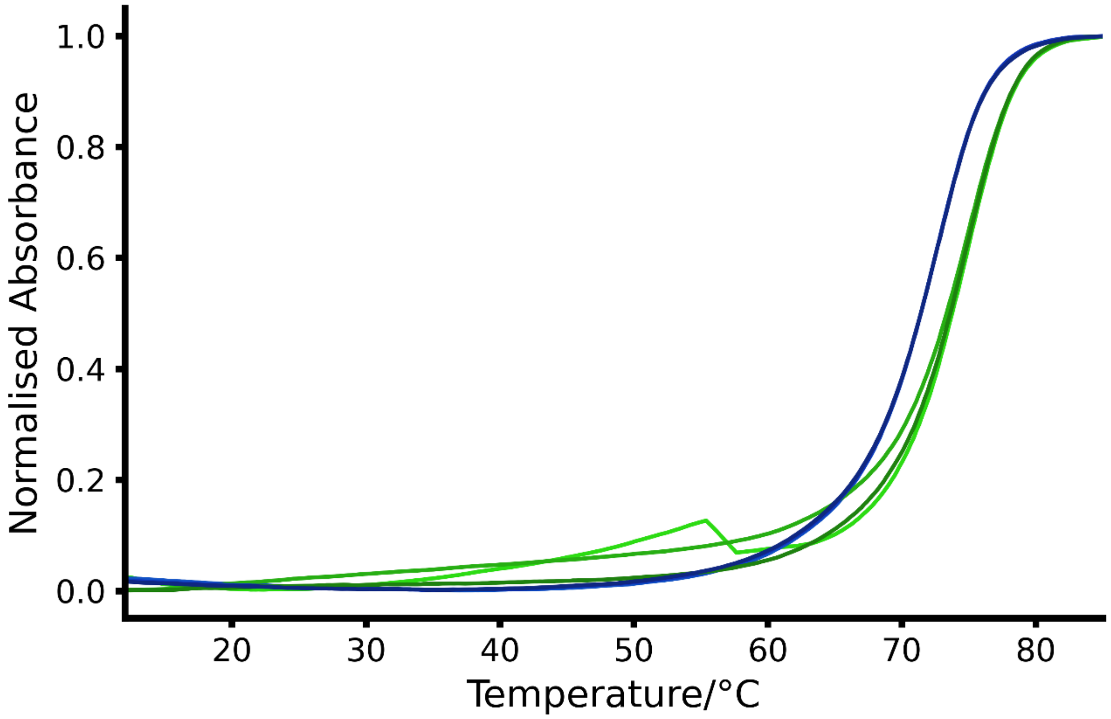

Supplementary Figure 25: Melting Temperature Curves of 1PS ASO

1PSNV ASO

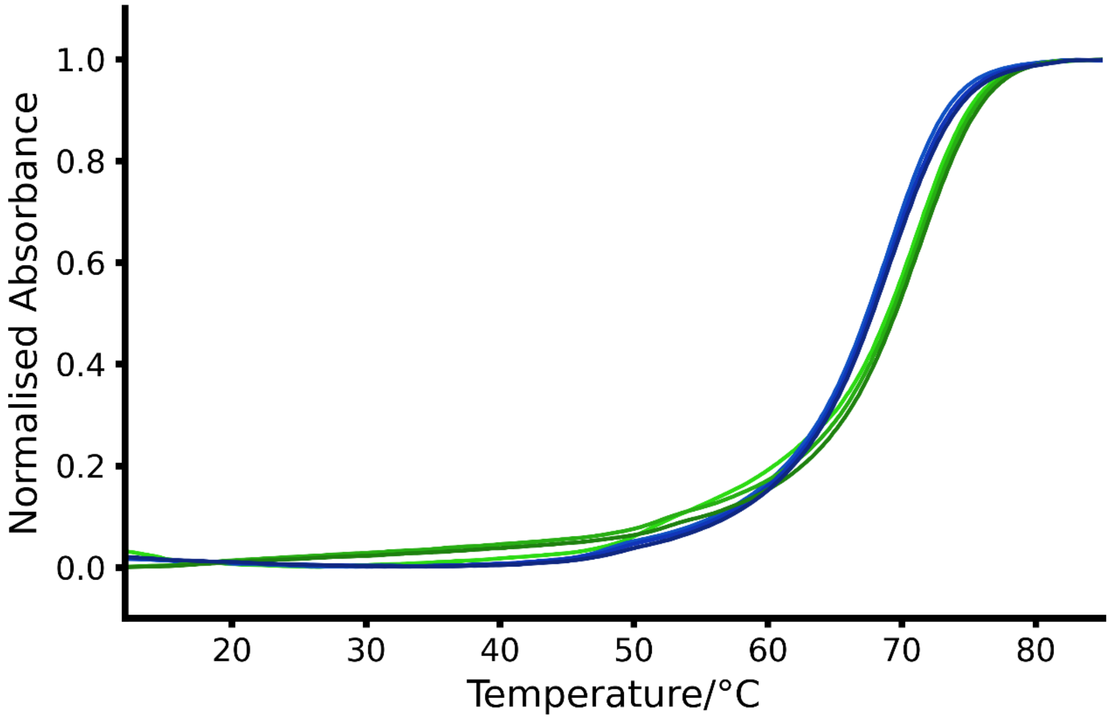

Supplementary Figure 26: Melting Temperature Curves of 1PSNV ASO

6PS ASO

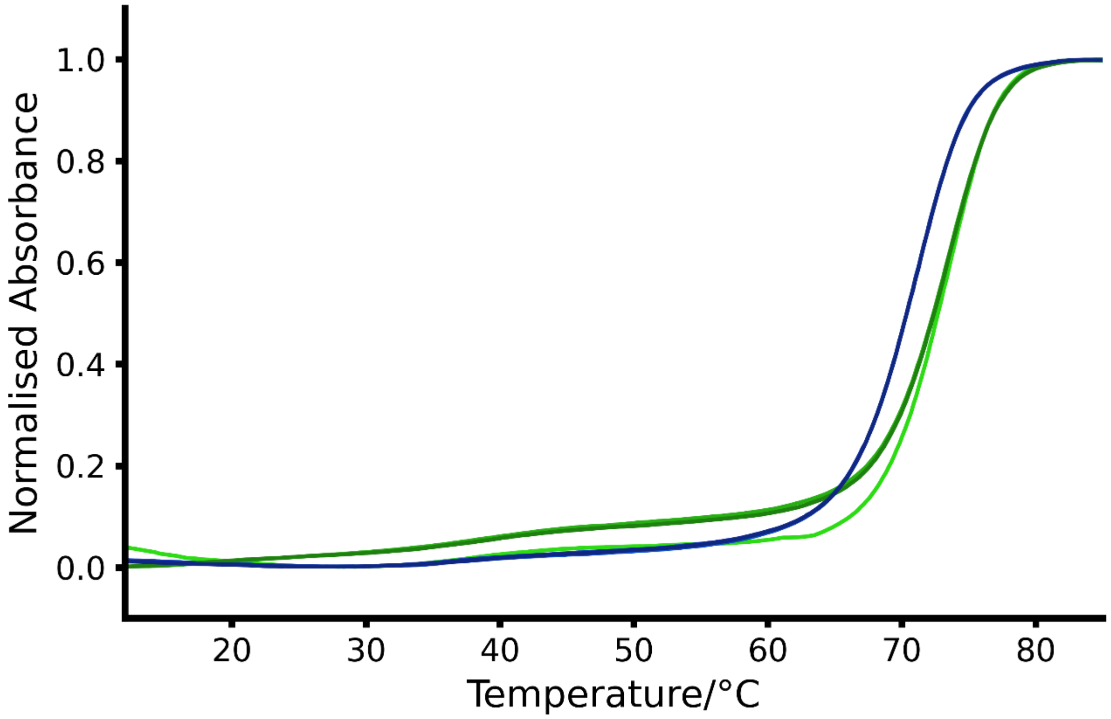

Supplementary Figure 27: Melting Temperature Curves of 6PS ASO

6PSNV ASO

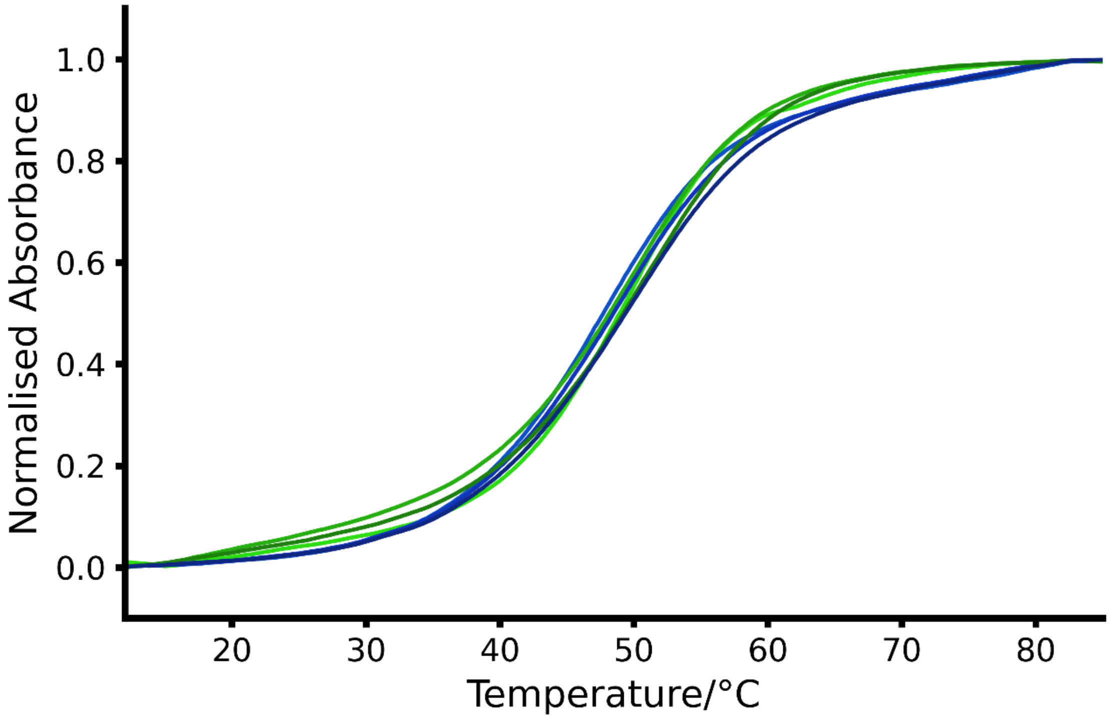

Supplementary Figure 28: Melting Temperature Curves of 6PSNV ASO

9PS ASO

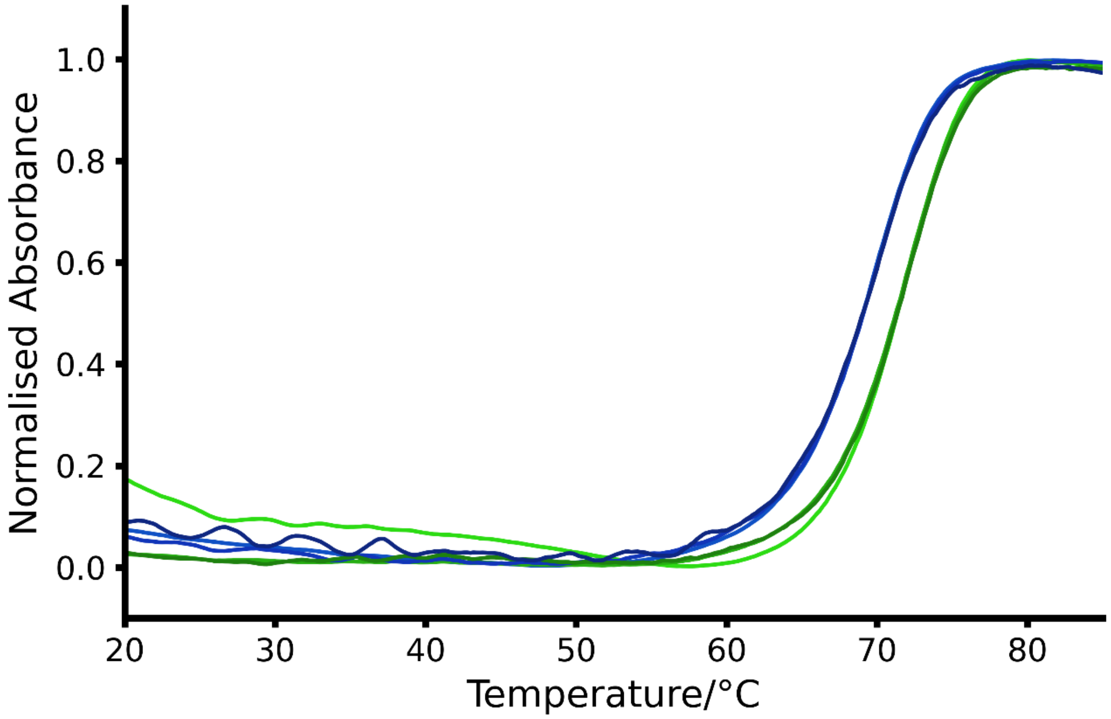

Supplementary Figure 29: Melting Temperature Curves of 9PS ASO

9PSNV ASO

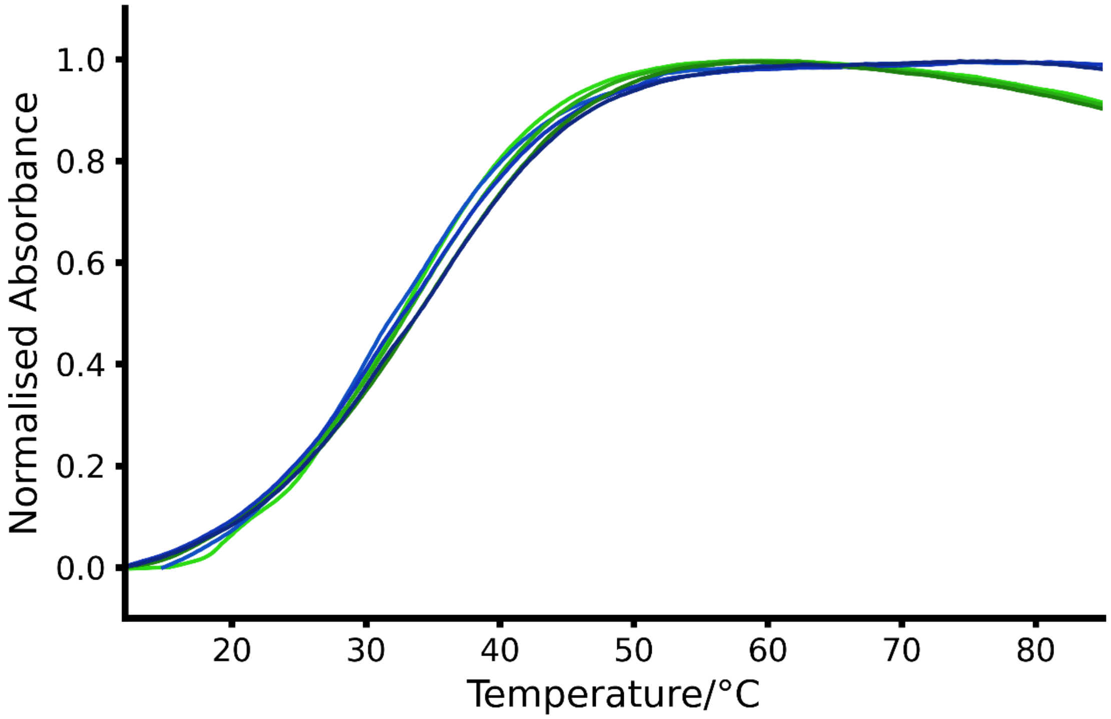

Supplementary Figure 30: Melting Temperature Curves of 9PSNV ASO

1PSEt ASO

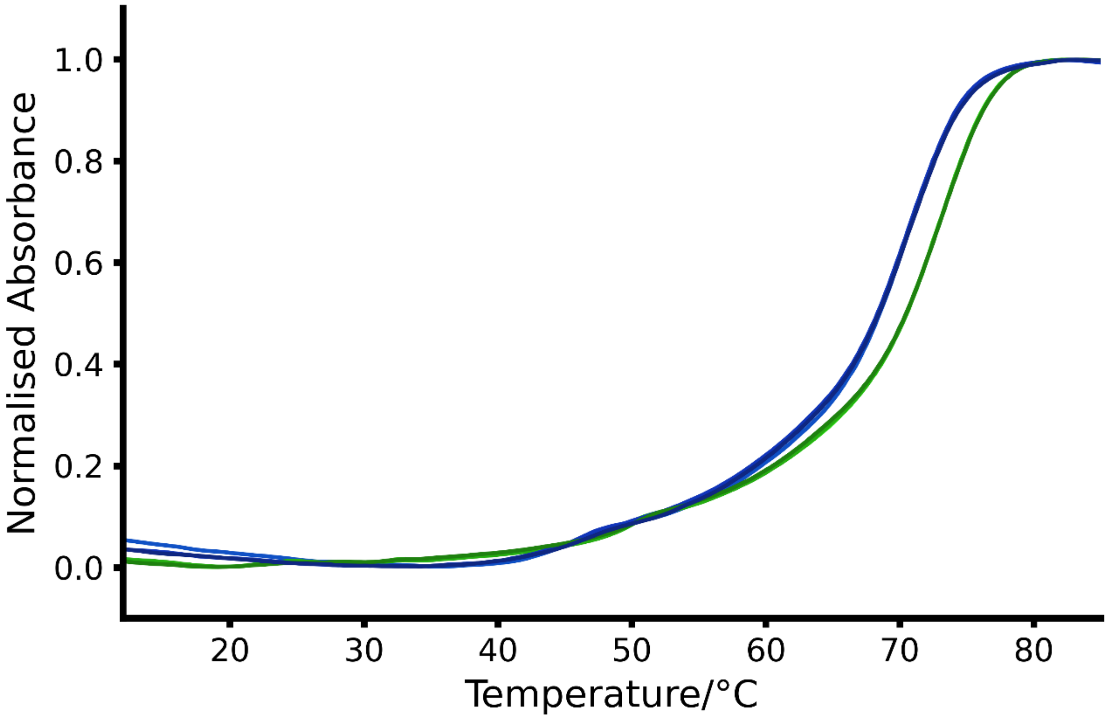

Supplementary Figure 31: Melting Temperature Curves of 1PSEt ASO

HPLC purification of oligonucleotides

Oligonucleotides were purified on an Agilent 1260 Infinity High Performance Liquid Chromatography instrument equipped with an Agilent Polaris C18 column (4.6 x 150 mm) heated to 50 °C using a gradient of 5 to 37% MeCN over 20 minutes with 10 mM NH_4OAc pH 7.5 throughout, with the exception of the 1PSEt oligonucleotide, which was purified using a gradient of 2% MeCN for 1 minute, then 2-10% MeCN.over 19 minutes.

1PSEt

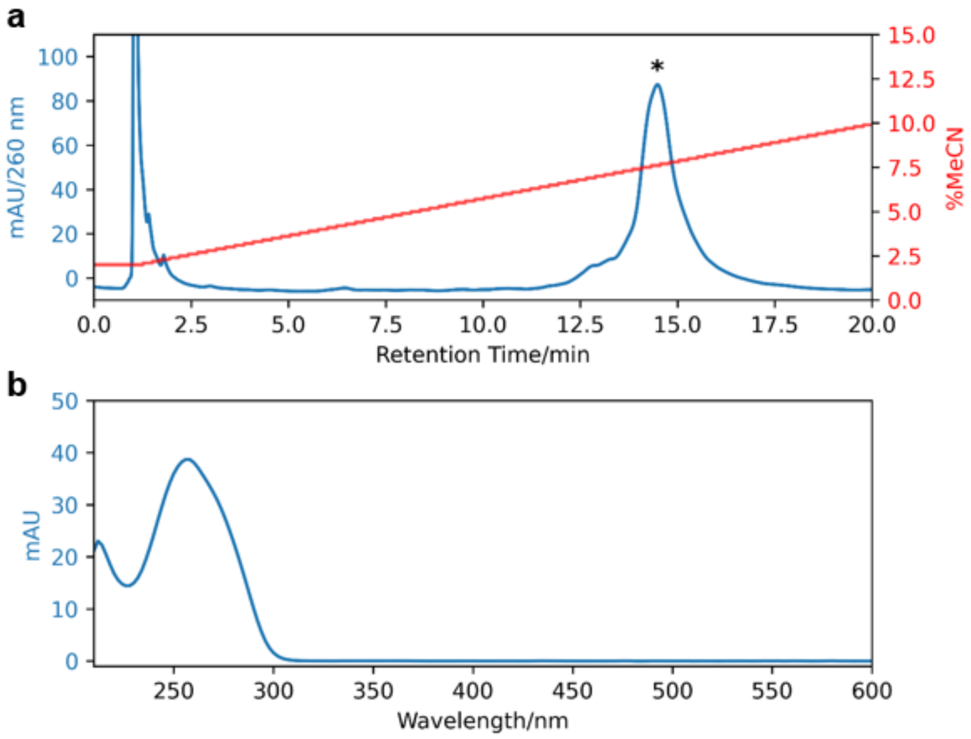

HPLC Purification of ethyl-modified 1PS-oligonucleotide (**Supplementary Table 3**, **Entry 1**). Crude reaction mixture and the absorbance spectrum of the *-labelled peak.

1PSNV

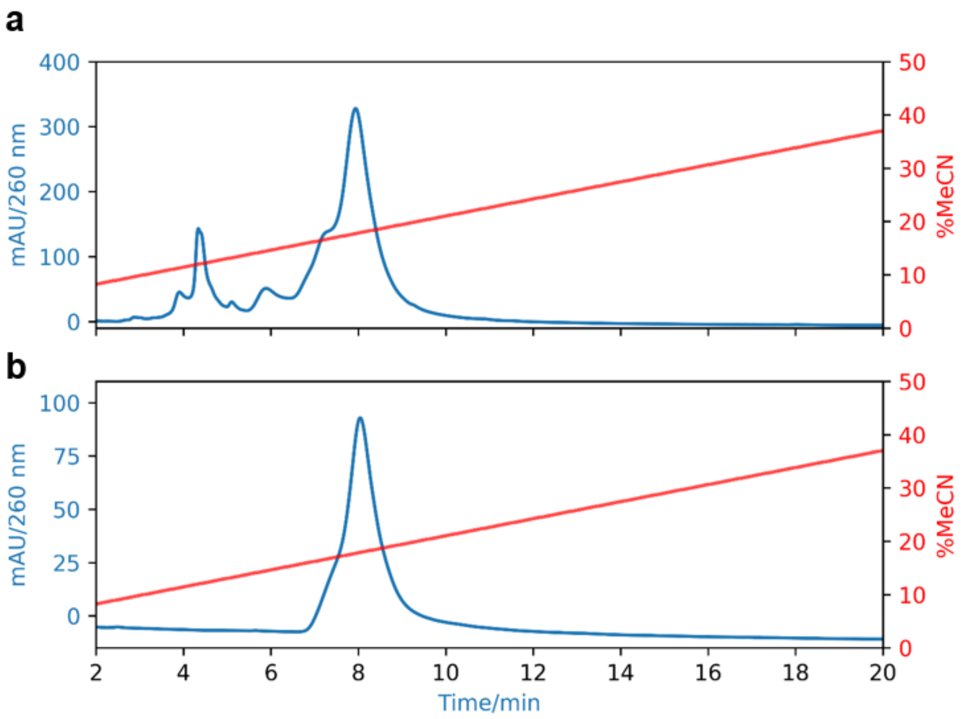

HPLC Purification of nitroveratryl-modified 1PS-oligonucleotide **(Supplementary Table 3, Entry 1**). **a)** Crude reaction mixture. **b)** Purified oligonucleotide.

6PSNV

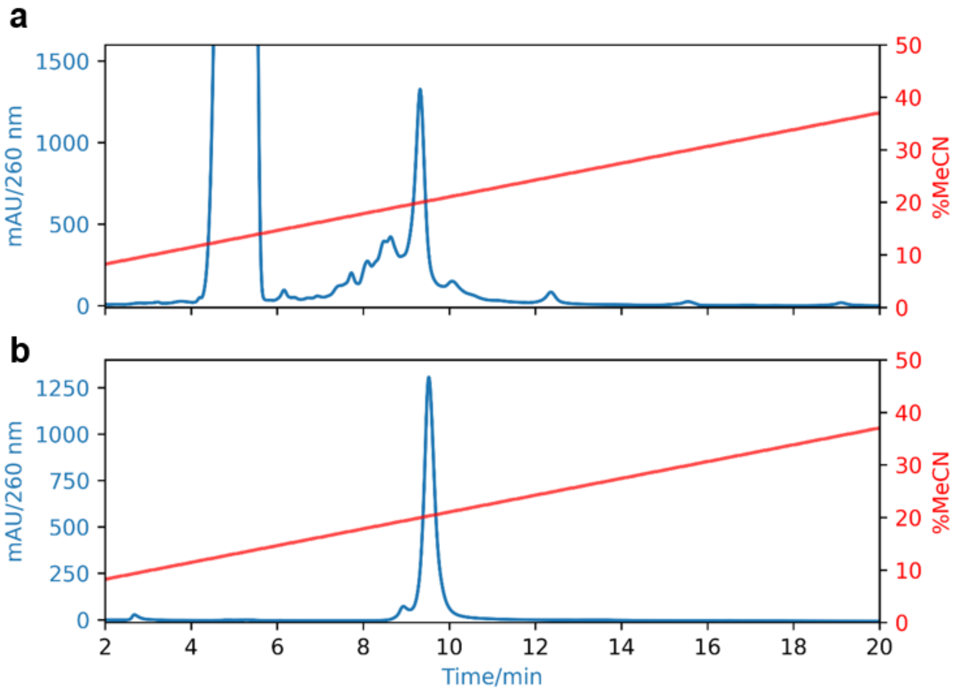

HPLC Purification of nitroveratryl-modified 6PS-oligonucleotide (**Supplementary Table 3, Entry 2**). **a)** Crude reaction mixture. **b)** Purified oligonucleotide.

9PSNV

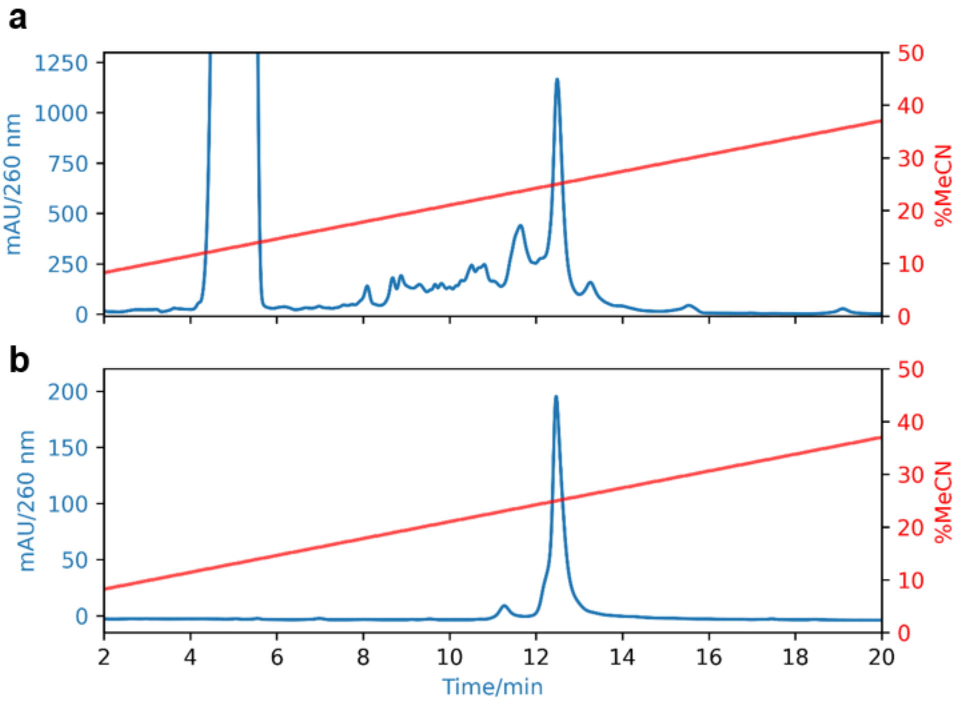

HPLC Purification of nitroveratryl-modified 9PS-oligonucleotide (**Supplementary Table 3, Entry 3**). **a)** Crude reaction mixture. **b)** Purified oligonucleotide.

TxRd-20nt-6PSNV

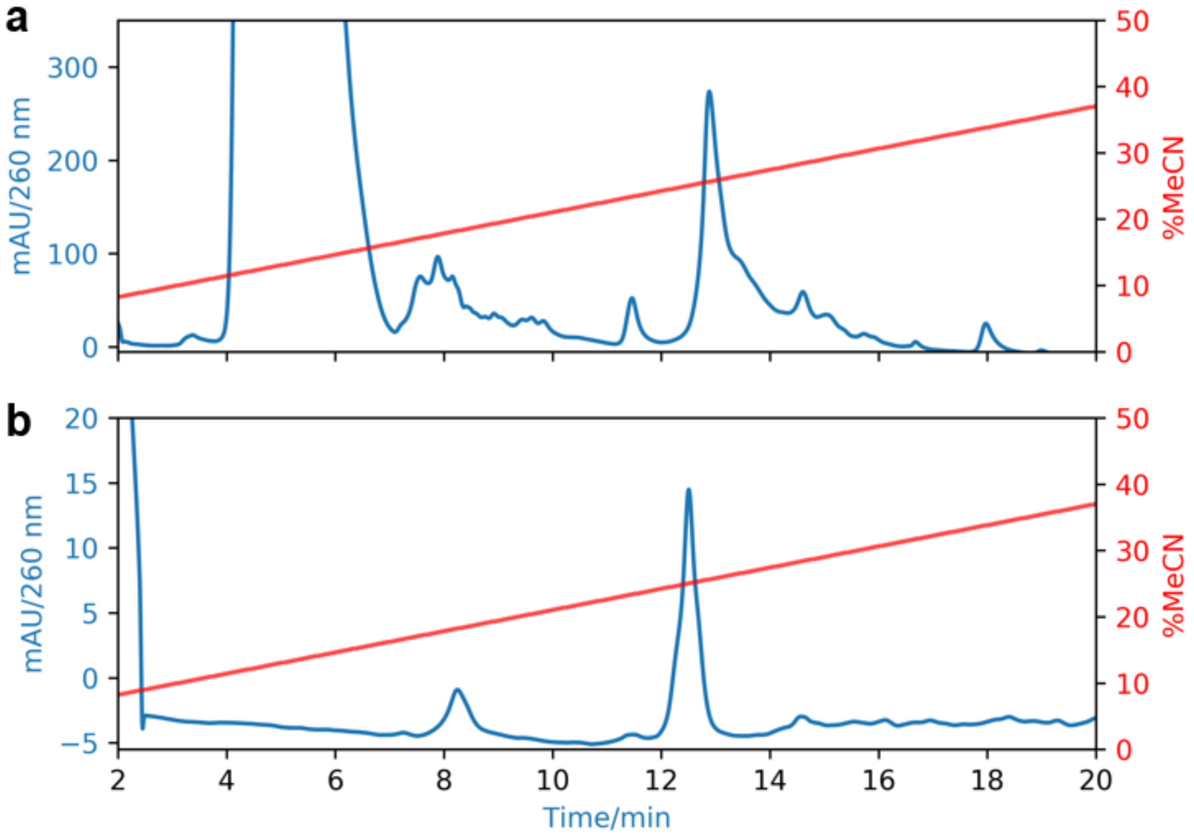

HPLC Purification of TxRd-tagged nitroveratryl-modified 6PS-oligonucleotide (**Supplementary Table 3, Entry 4**). **a**) Crude reaction mixture. **b**) Purified oligonucleotide.

TxRd-20nt-9PSNV

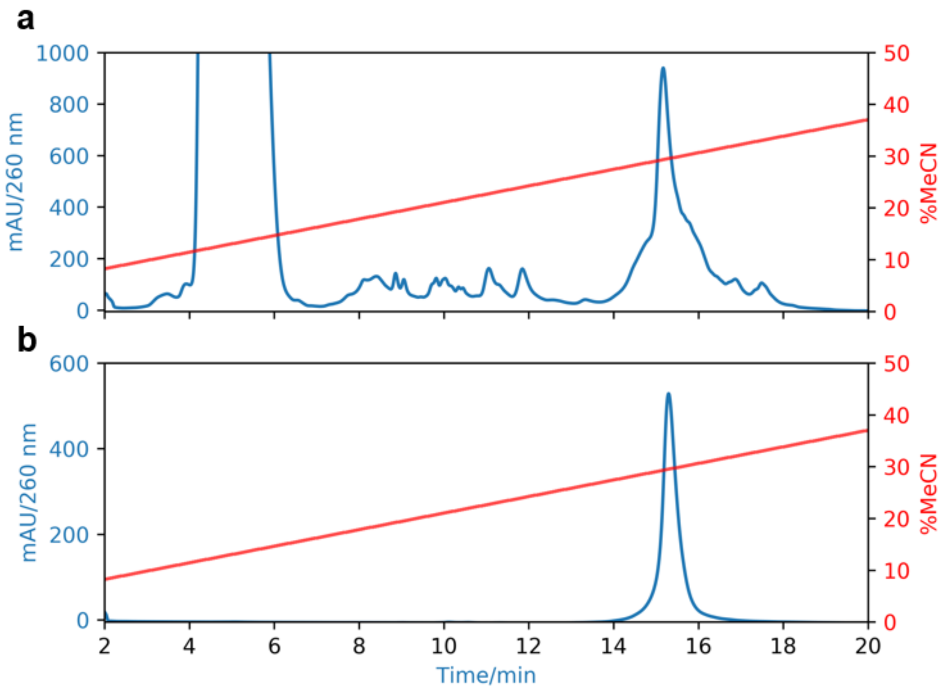

HPLC Purification of TxRd-tagged nitroveratryl-modified 9PS-oligonucleotide (**Supplementary Table 3, Entry 5**). **a**) Crude reaction mixture. **b**) Purified oligonucleotide.

TxRd-14nt-6PSNV

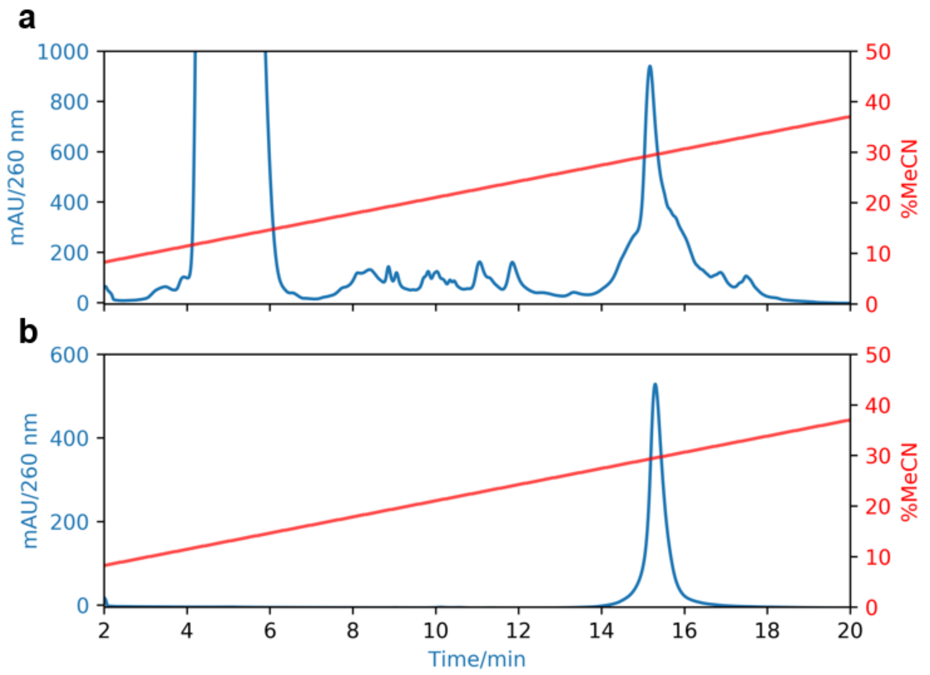

HPLC Purification of TxRd-tagged nitroveratryl-modified 14nt-ON (**Supplementary Table 3, Entry 6**). **a**) Crude reaction mixture. **b**) Purified oligonucleotide.

Reaction screening oligonucleotide mass spectrometry

Oligonucleotide Mass Spectra were recorded on a Waters Xevo G2 QTOF ESI- UPLC-MS system. A gradient of MeOH in Et_3N and hexafluoroisopropanol (HFIP) was used (buffer A, 8.6 mM Et_3N, 200 mM HFIP in 5% MeOH/H_2O (v/v); buffer B, 20% buffer A in MeOH).[3] Samples were analysed using a gradient of 0 to 70% buffer B over 8 minutes and data was then deconvoluted using MassLynx v4.1.

Entry	Oligonucleotide	Buffer	Retention Time/minutes	Masses after Deconvolution (Expected Mass) – Modification
**1**	1PS	$NaHCO_3$	5.18	6220 (6220) – desulfurisation
**2**	1PS	$NaHCO_3$	5.40	6431 (6431) – 1x 2-NV
**3**	1PS	$NaHCO_3$	5.5-6.5	6416 (6415) – Desulfurisation and 1x 2-NV<br>6611 (6610) – Desulfurisation and 2x 2-NV<br>6625 (6626) – 2x 2-NV<br>6806 (6806) - Desulfurisation and 3x 2-NV<br>6823 (6821) – 3x 2-NV<br>6999 (7001) - Desulfurisation and 4x 2-NV<br>7016 (7016) – 4x 2-NV<br>7210 (7211) – 5x 2-NV<br>7408 (7406) – 6x 2-NV
**4**	1PS	Tris pH 8	5.20	6219 (6220) – desulfurisation
**5**	1PS	Tris pH 8	5.40	6431 (6431) – 1x 2-NV
**6**	1PS	Tris pH 8	5.56	6626 (6626) – 2x 2-NV
**7**	1PS	HEPES pH 7	5.40	6431 (6431) – 1x 2-NV
**8**	PO-only	HEPES pH 7	5.18	6219 (6220) – native oligonucleotide

Supplementary Table 10: Oligonucleotide masses under different reaction conditions, as measured by LC-MS.

Condition 1: $NaHCO_3$ buffer chromatogram

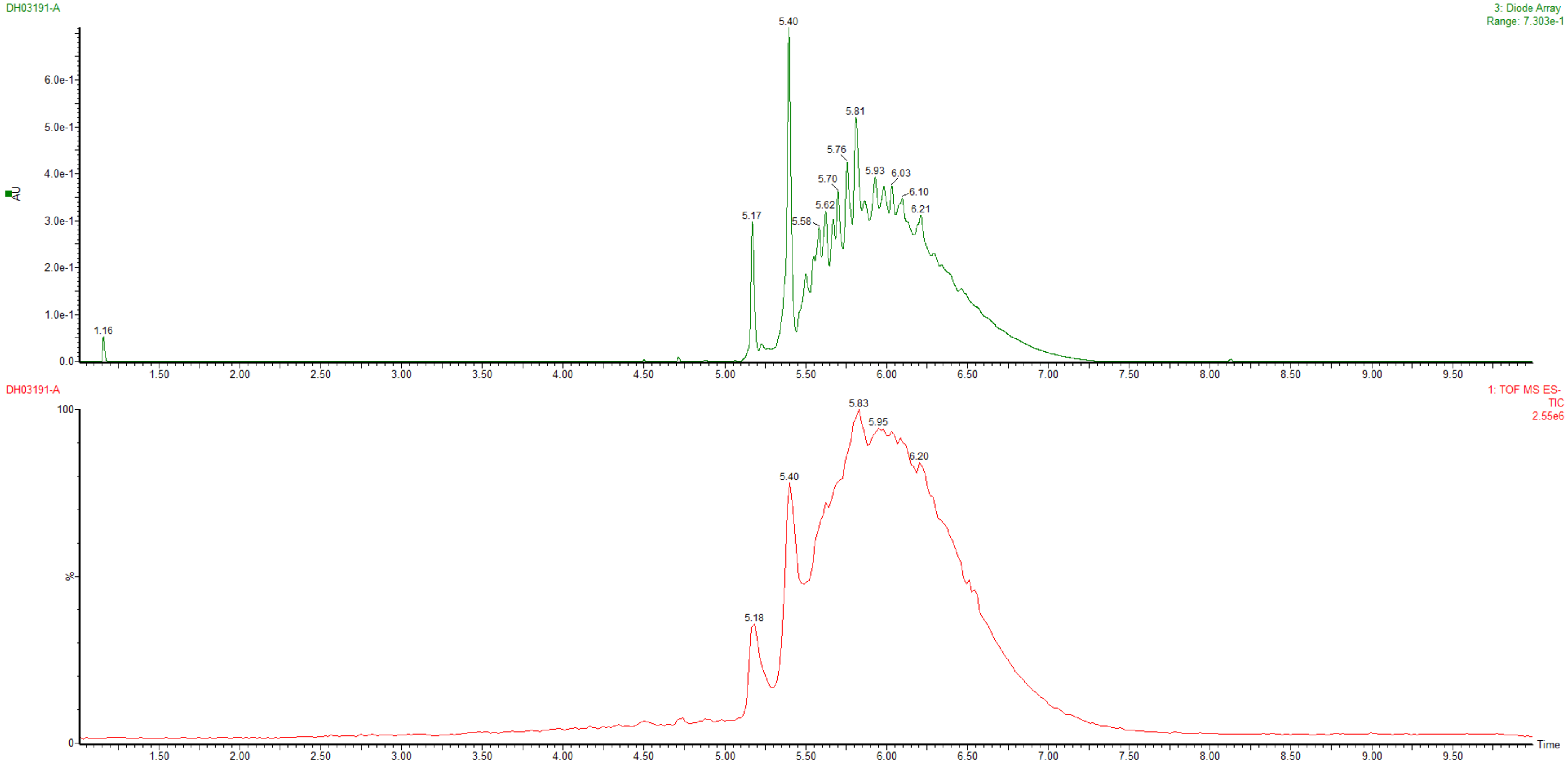

Condition 1: $NaHCO_3$ buffer peak 5.18

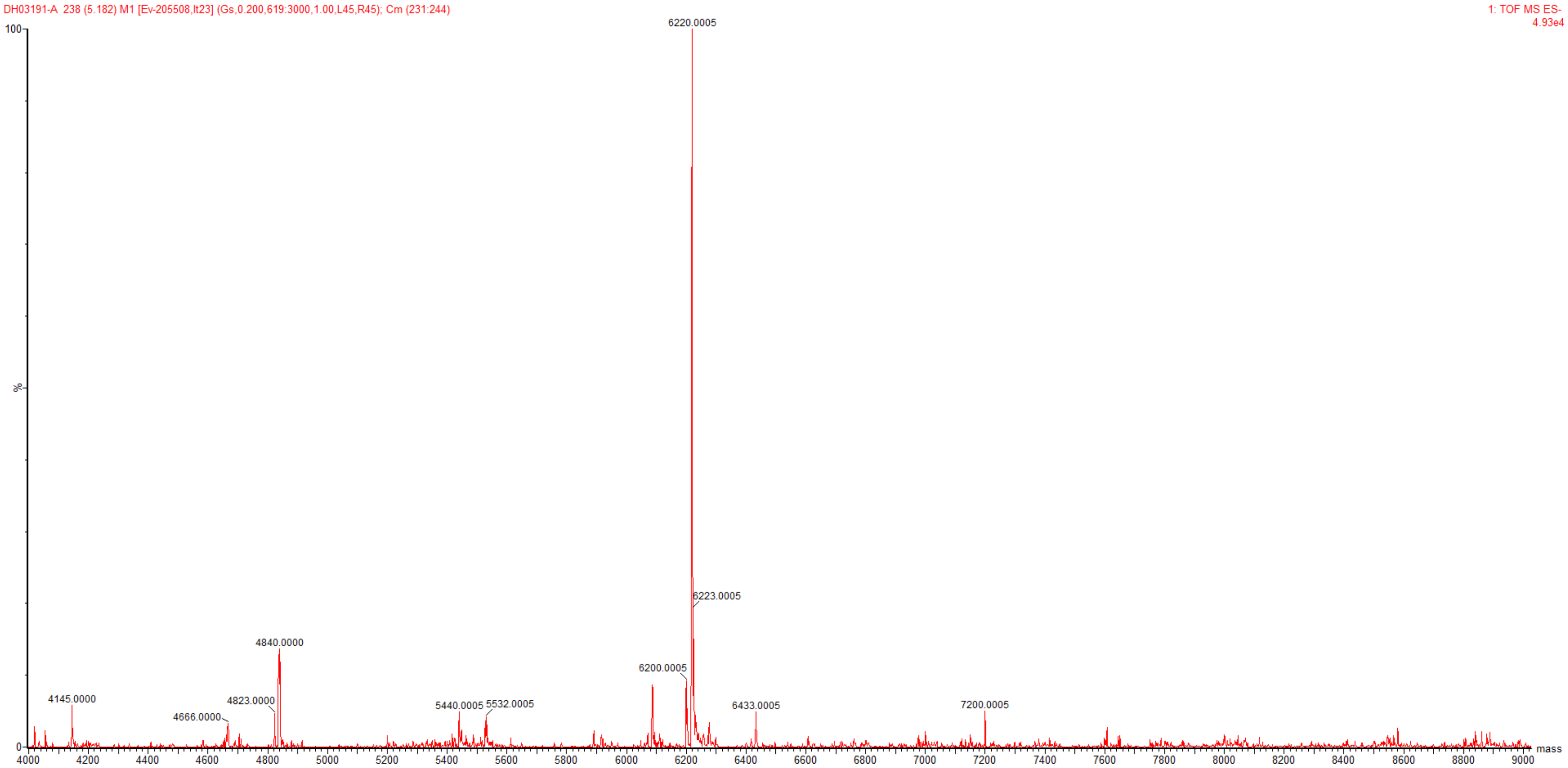

Condition 1: $NaHCO_3$ buffer peak 5.40

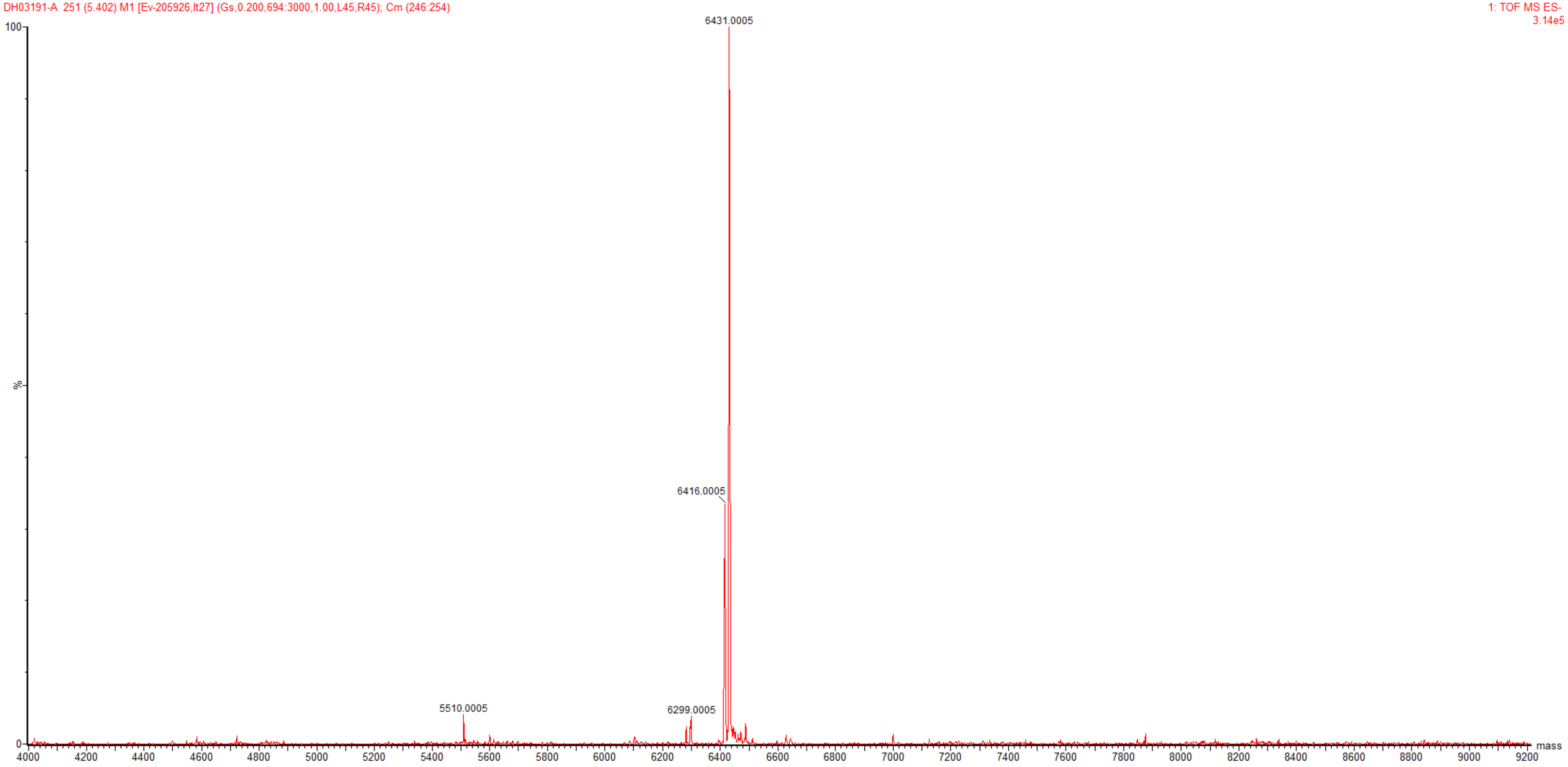

Condition 1: $NaHCO_3$ buffer peak 5.5-6.5

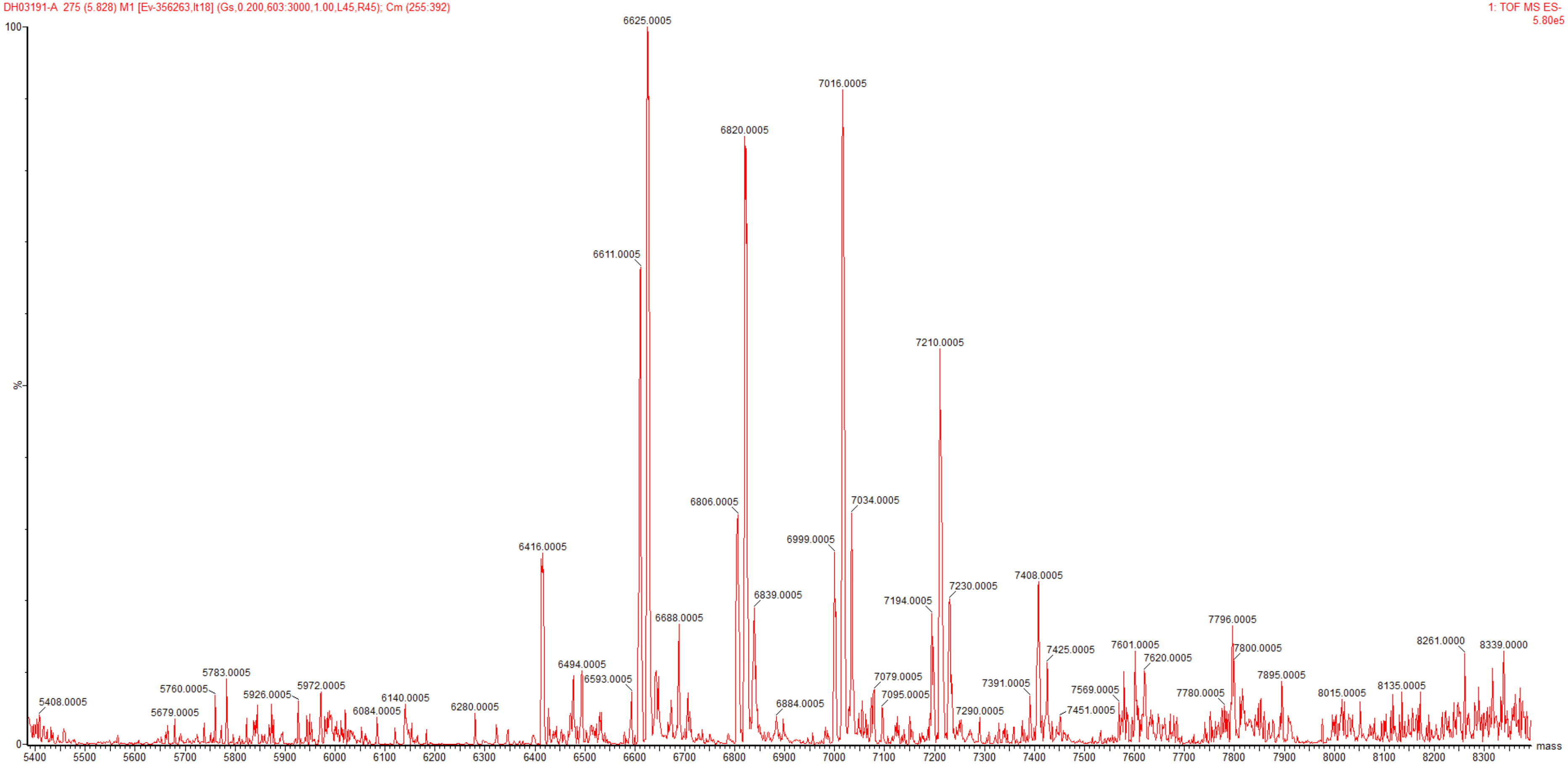

Condition 2: Tris buffer chromatogram

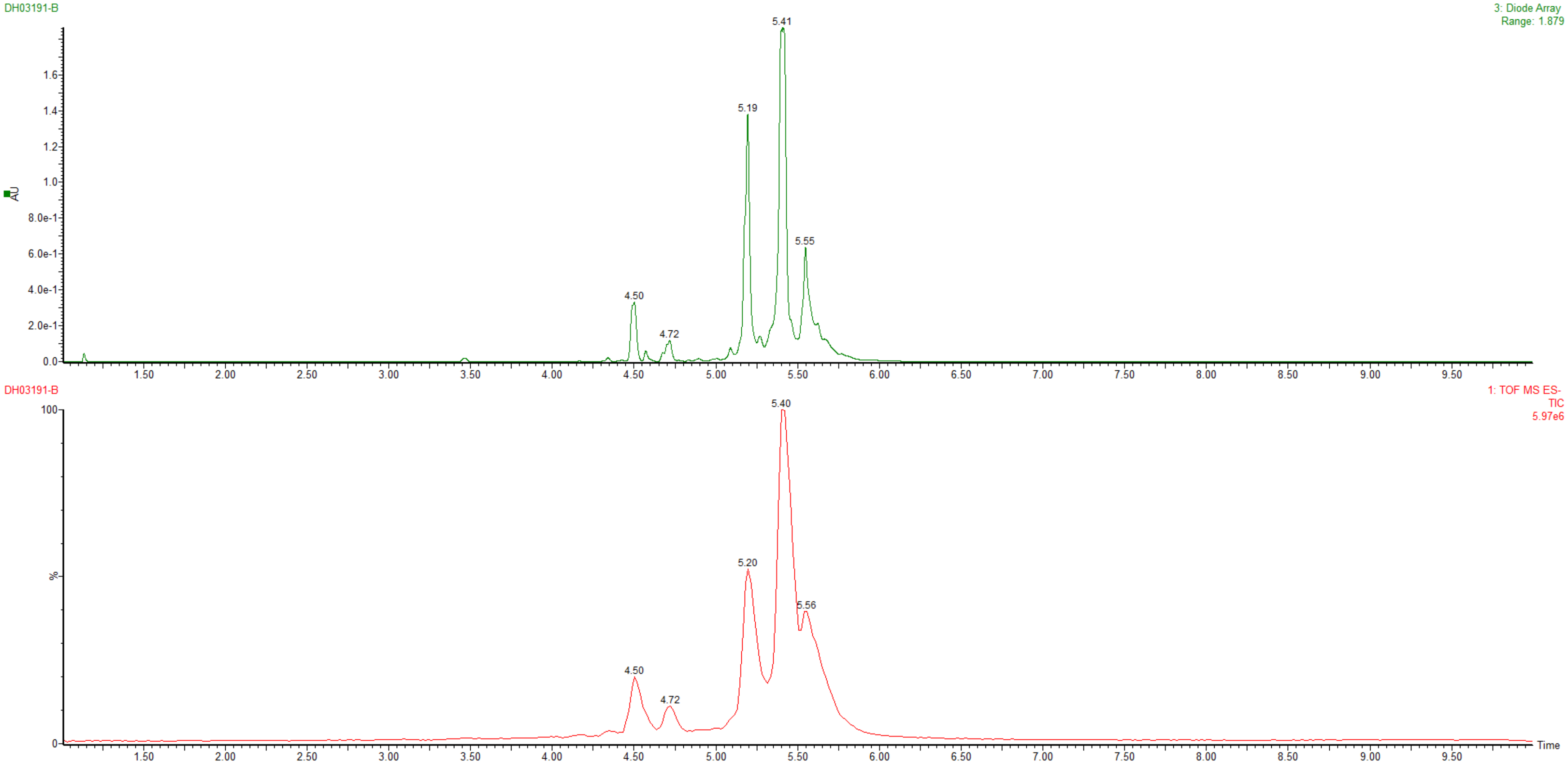

Condition 2: Peak at 5.20 minutes

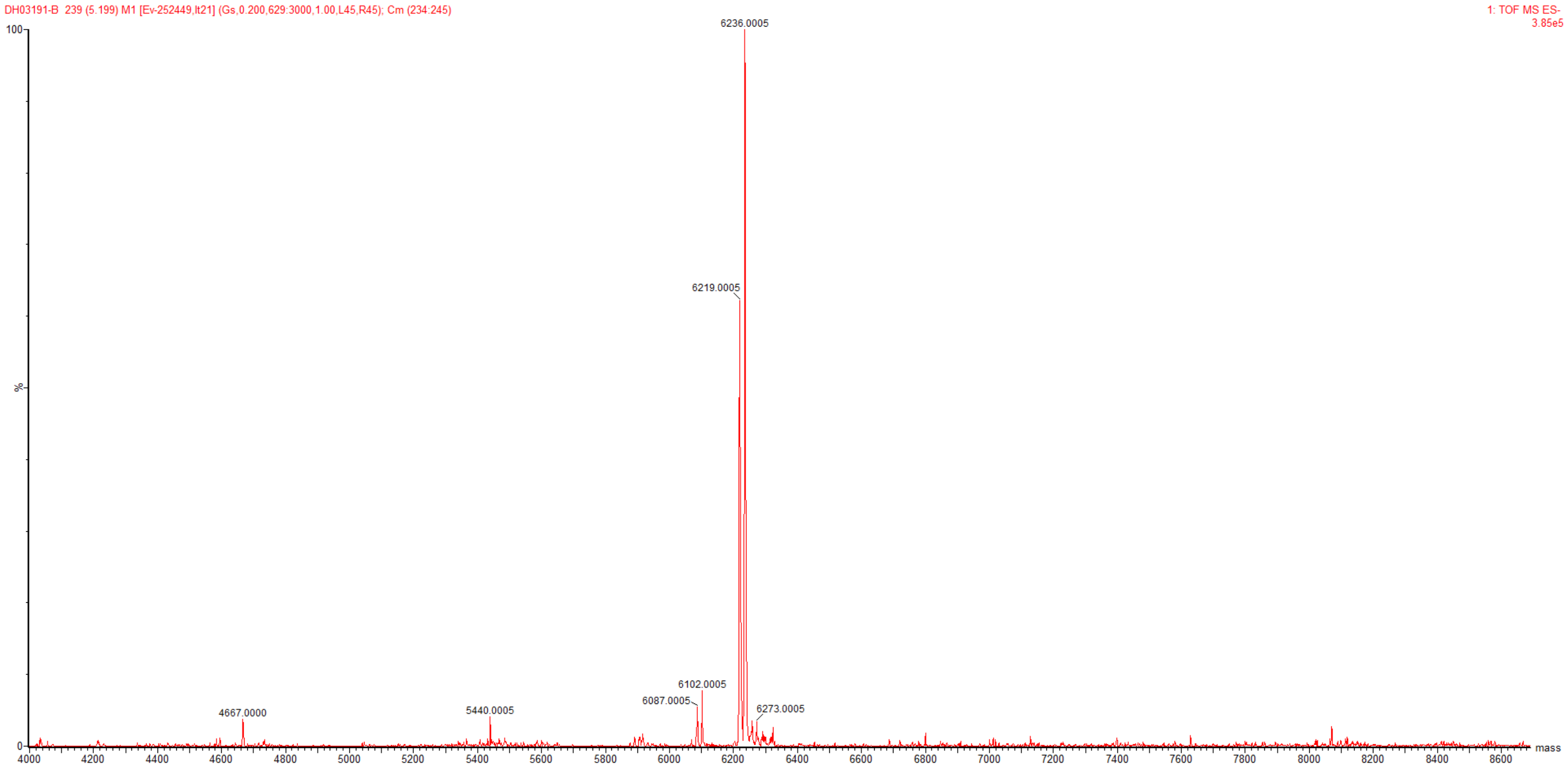

:

Condition 2: Peak at 5.40 minutes

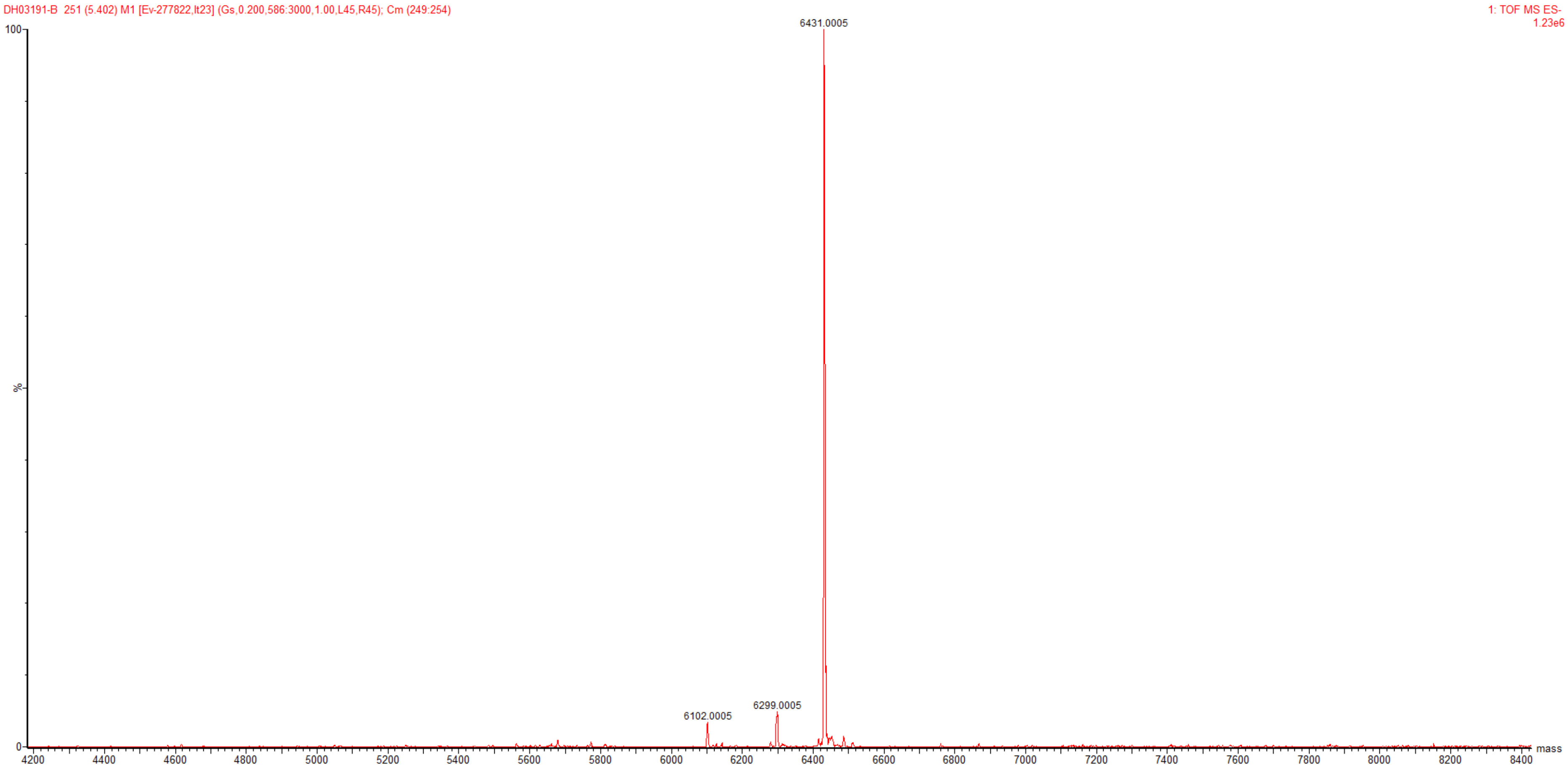

Condition 2: Peak at 5.56 minutes

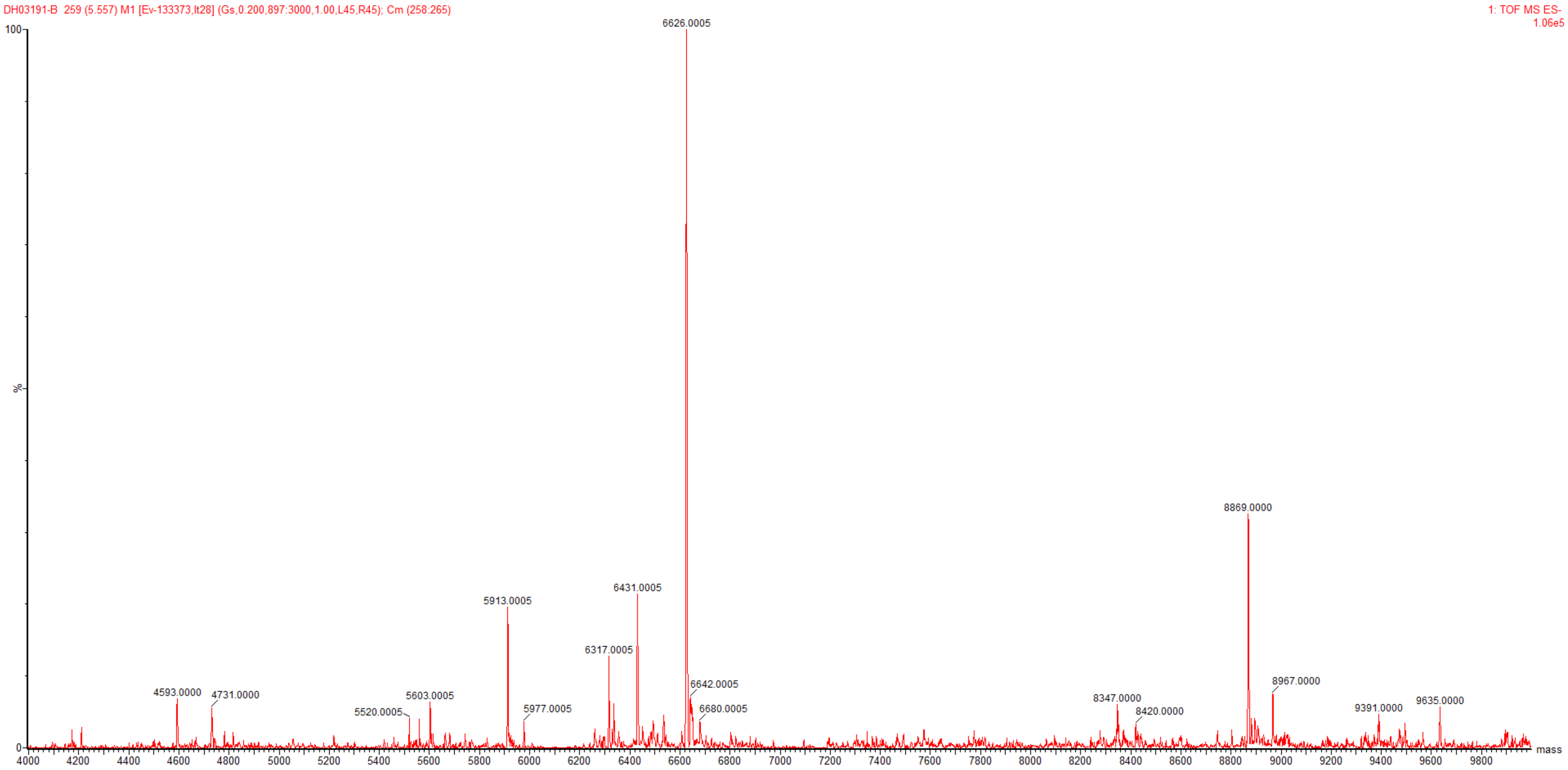

Condition 3: HEPES buffer chromatogram

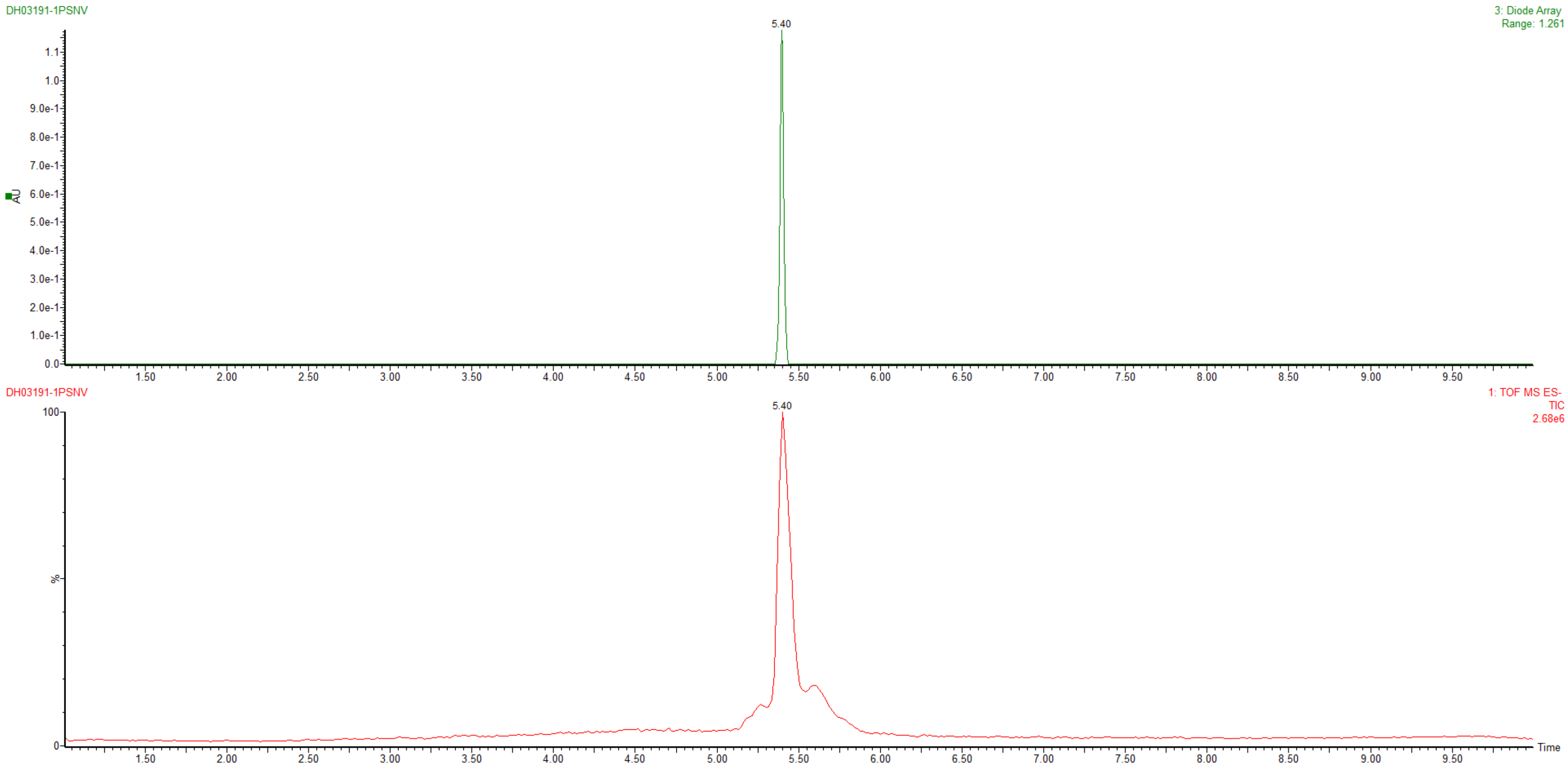

Condition 3: Peak at 5.40

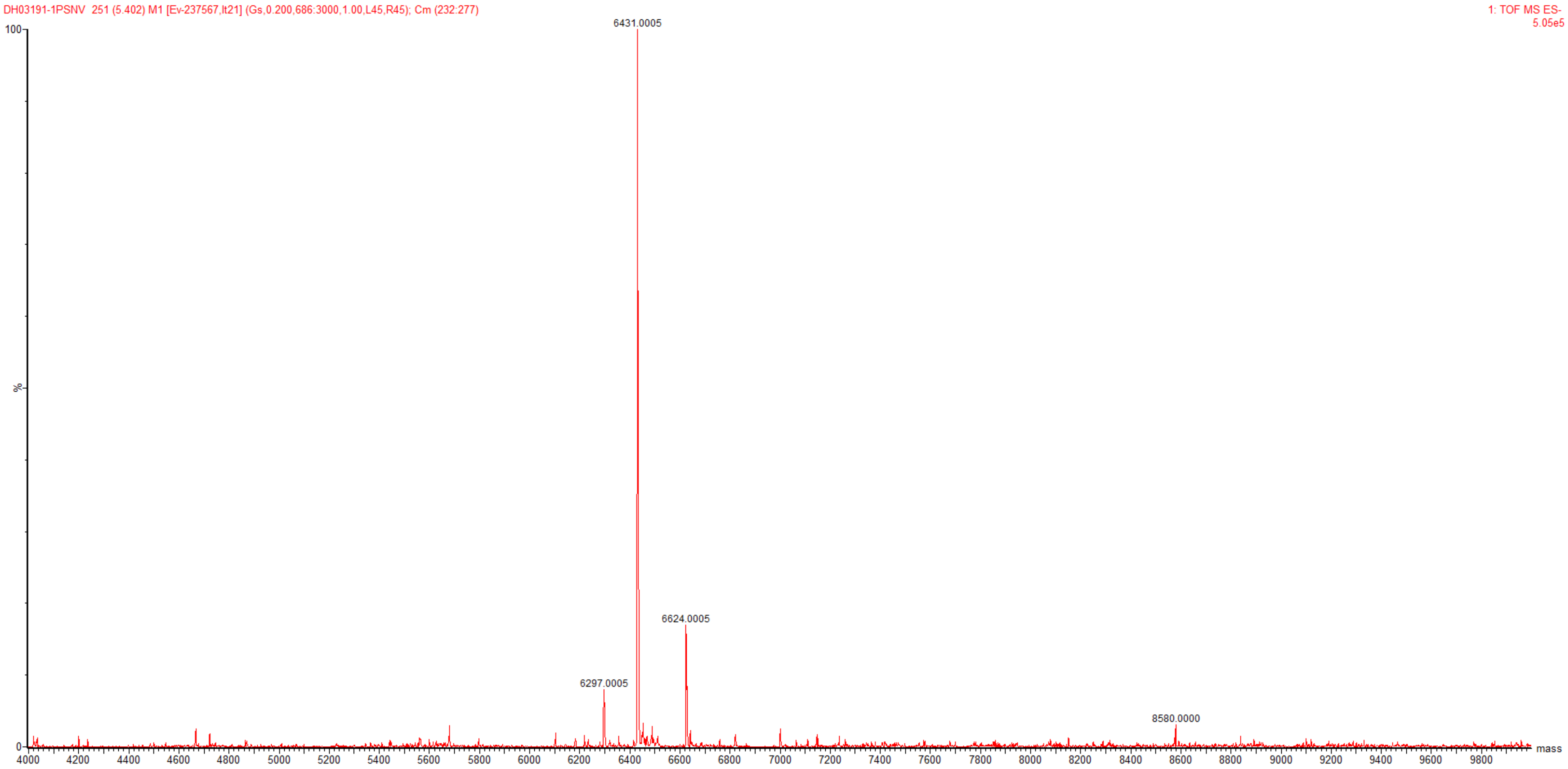

PO-only control with HEPES buffer chromatogram

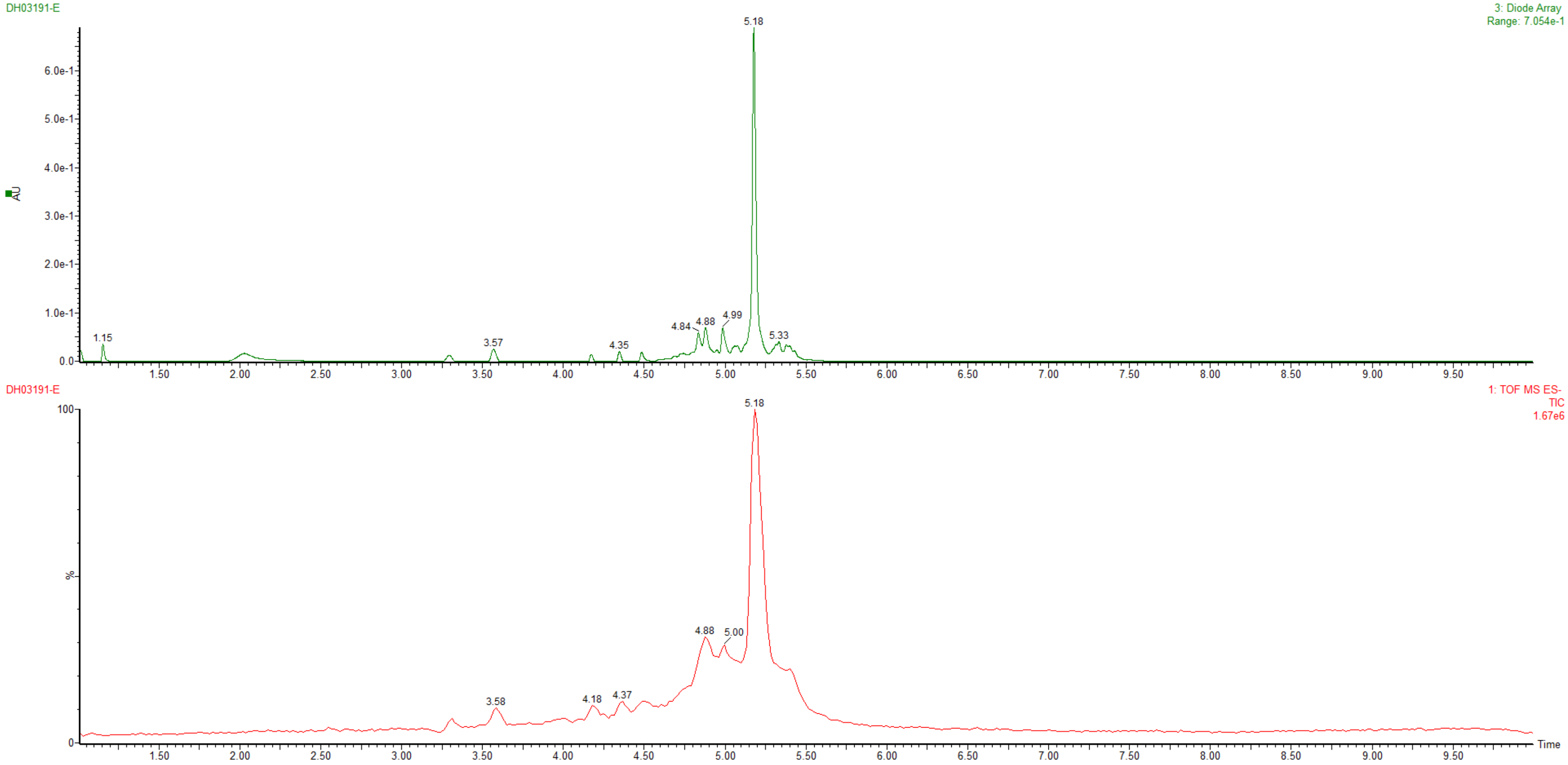

PO-only control with HEPES buffer peak at 5.18

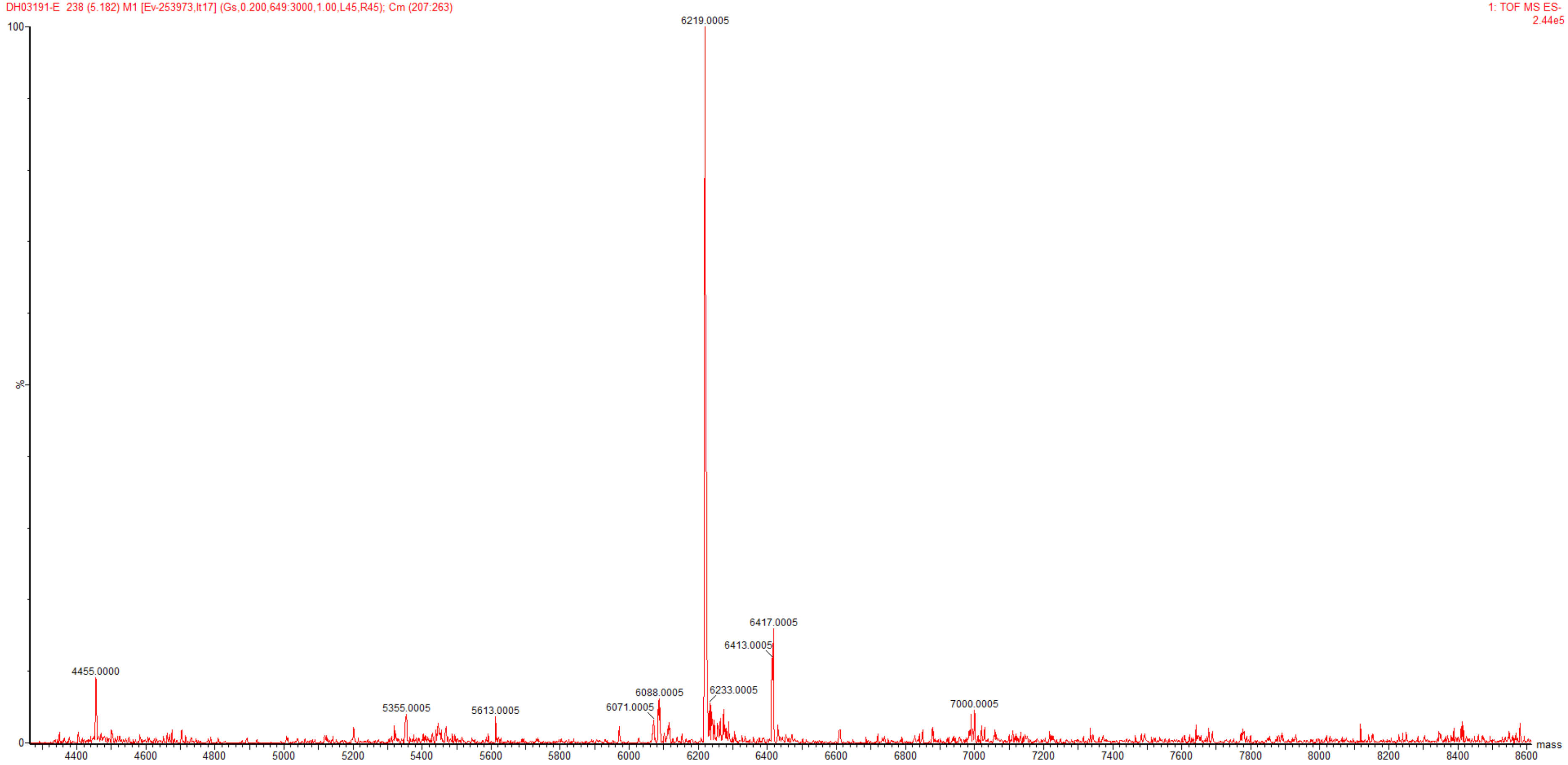

Oligonucleotide mass spectra

Oligonucleotide mass spectrometry

Oligonucleotide Mass Spectra were recorded on a Waters Xevo G2 QTOF ESI- UPLC-MS system. A gradient of MeOH in Et_3N and hexafluoroisopropanol (HFIP) was used (buffer A, 8.6 mM Et_3N, 200 mM HFIP in 5% MeOH/H_2O (v/v); buffer B, 20% buffer A in MeOH).[3] DNA samples with 1 modification were analysed using a gradient of 0 to 70% B over 8.5 minutes. DNA samples with more than one modification were analysed using a stepwise gradient from 0% (min 0) to 20% (1 minute) to 80% (minute 8.5) to 100% (minute 8.6) of Buffer B. Data was then deconvoluted using MassLynx v4.1.

Entry	Oligonucleotide	Mass after Deconvolution	Expected Mass
**1**	1PSEt	6265	6265
**2**	1PSNV (See above)	6431	6431
**3**	6PSNV	7487	7486
**4**	9PSNV	8121	8120
**5**	20nt-TxRd-6PSNV	8368	8368
**6**	20nt-TxRd-9PSNV	9002	9001
**7**	14nt-TxRd-6PSNV	6473	6473

Supplementary Table 11: Modified oligonucleotides and their masses after deconvolution as measured by LC-MS.

20nt-1PSEt

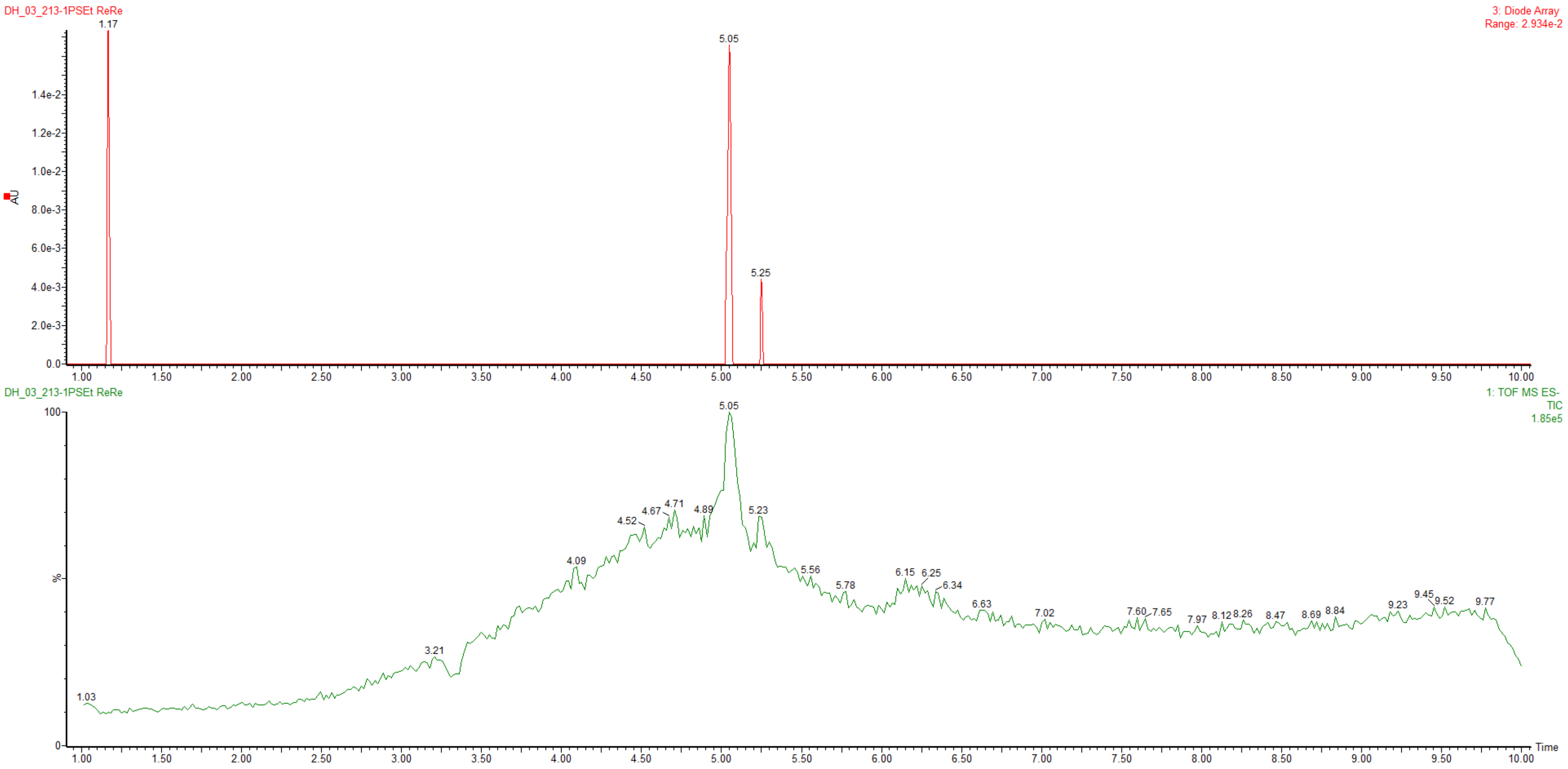
DH_03_213-1PSEt ReRe
3: Diode Array
Range: 2.934e-2
AU
1.17
5.05
5.25
1.4e-2
1.2e-2
1.0e-2
8.0e-3
6.0e-3
4.0e-3
2.0e-3
0.0
1.00 1.50 2.00 2.50 3.00 3.50 4.00 4.50 5.00 5.50 6.00 6.50 7.00 7.50 8.00 8.50 9.00 9.50 10.00
DH_03_213-1PSEt ReRe
1: TOF MS ES-
TIC
1.85e5
100
%
0
1.03
3.21
4.09
4.52
4.67
4.71
4.89
5.05
5.23
5.56
5.78
6.15
6.25
6.34
6.63
7.02
7.60
7.65
7.97
8.12
8.26
8.47
8.69
8.84
9.23
9.45
9.52
9.77
Time
1.00 1.50 2.00 2.50 3.00 3.50 4.00 4.50 5.00 5.50 6.00 6.50 7.00 7.50 8.00 8.50 9.00 9.50 10.00

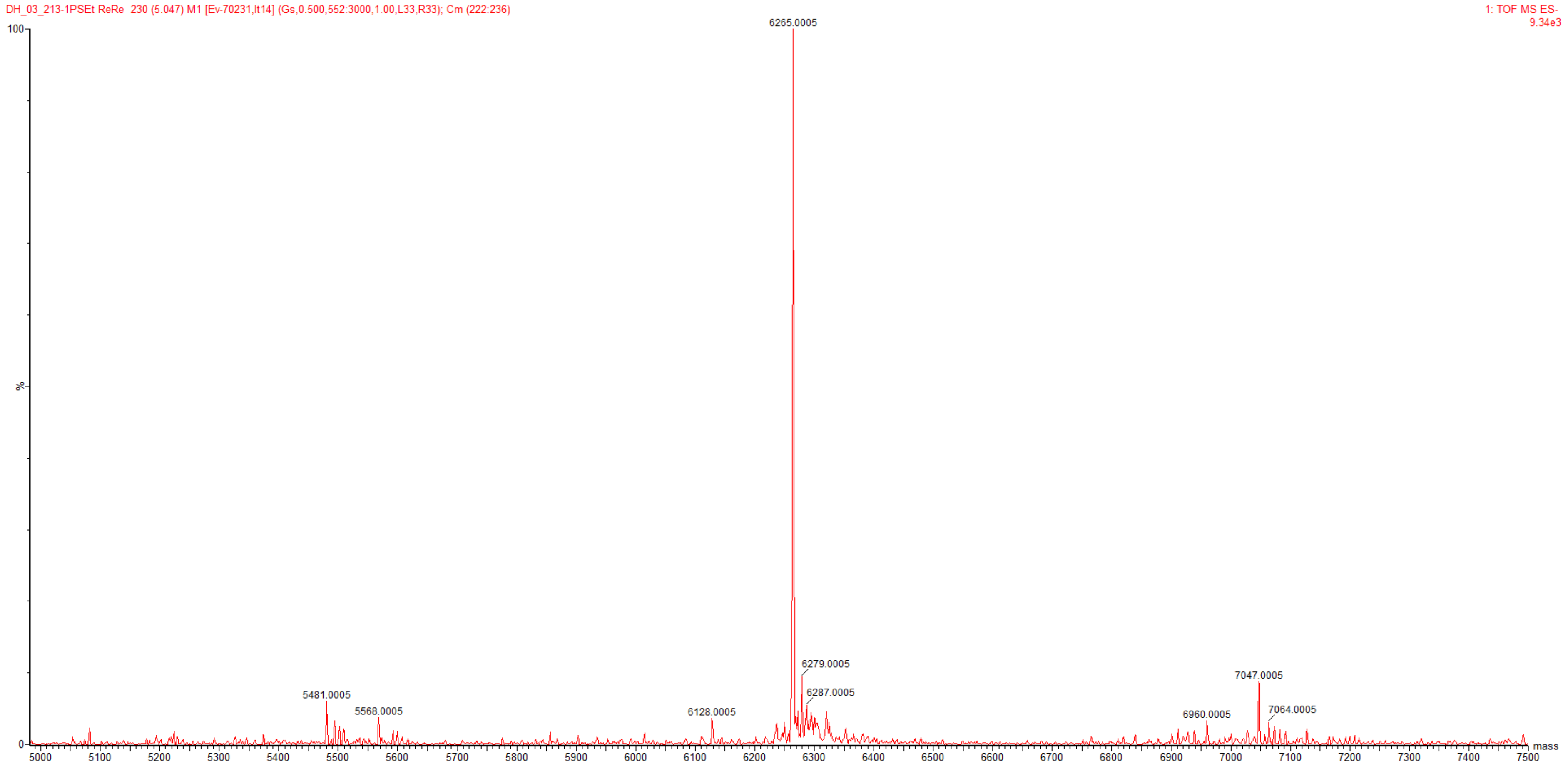
DH_03_213-1PSEt ReRe 230 (5.047) M1 [Ev-70231,It14] (Gs,0.500,552:3000,1.00,L33,R33); Cm (222:236)
1: TOF MS ES-
9.34e3
6265.0005
6279.0005
6287.0005
5481.0005
5568.0005
6128.0005
7047.0005
7064.0005
6960.0005
100
%
0
mass
5000
5100
5200
5300
5400
5500
5600
5700
5800
5900
6000
6100
6200
6300
6400
6500
6600
6700
6800
6900
7000
7100
7200
7300
7400
7500

20nt-6PSNV

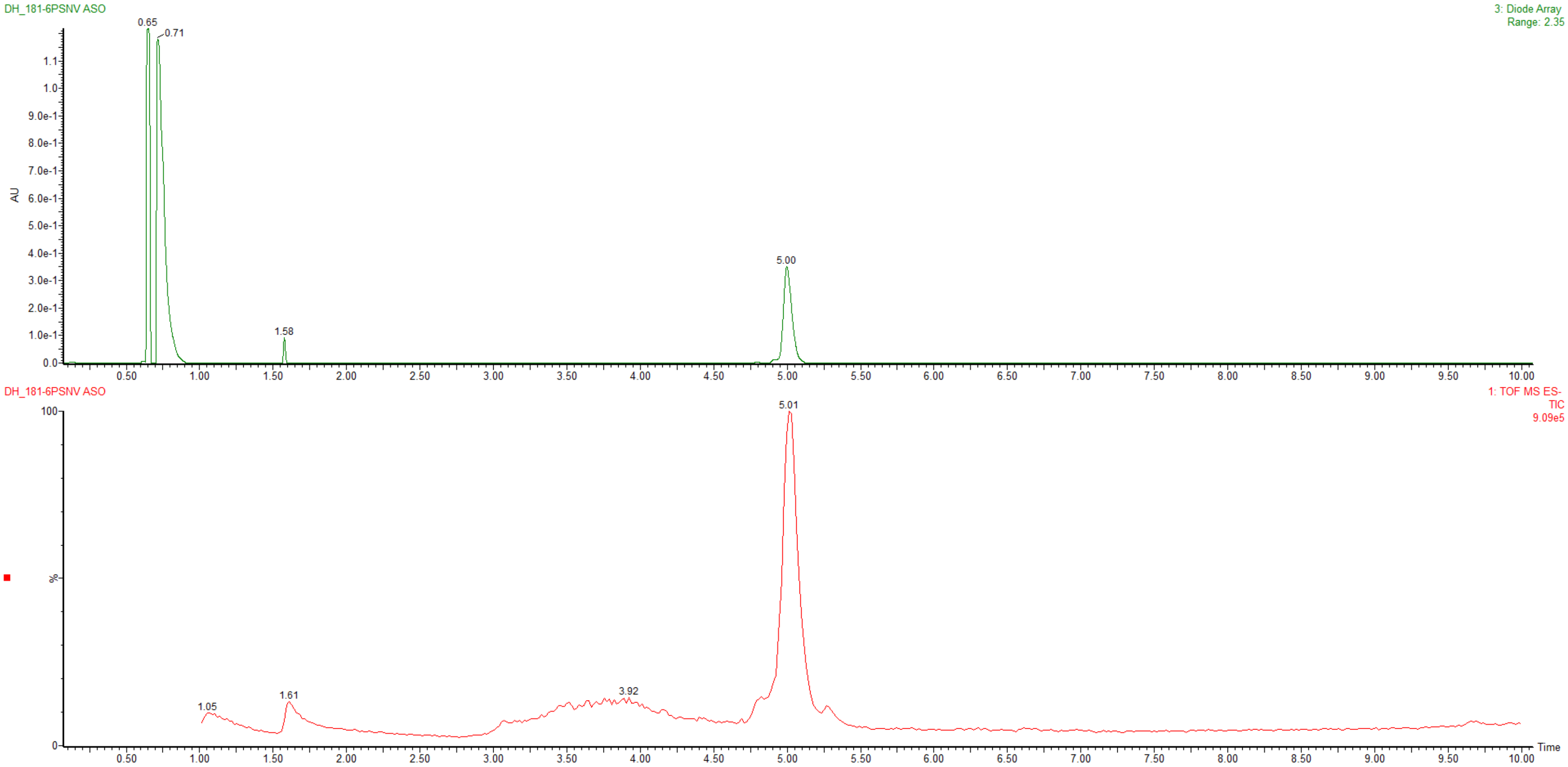

DH_181-6PSNV ASO
3: Diode Array
Range: 2.35
AU
0.65
0.71
1.58
5.00
DH_181-6PSNV ASO
1: TOF MS ES-
TIC
9.09e5
%
1.05
1.61
3.92
5.01
Time

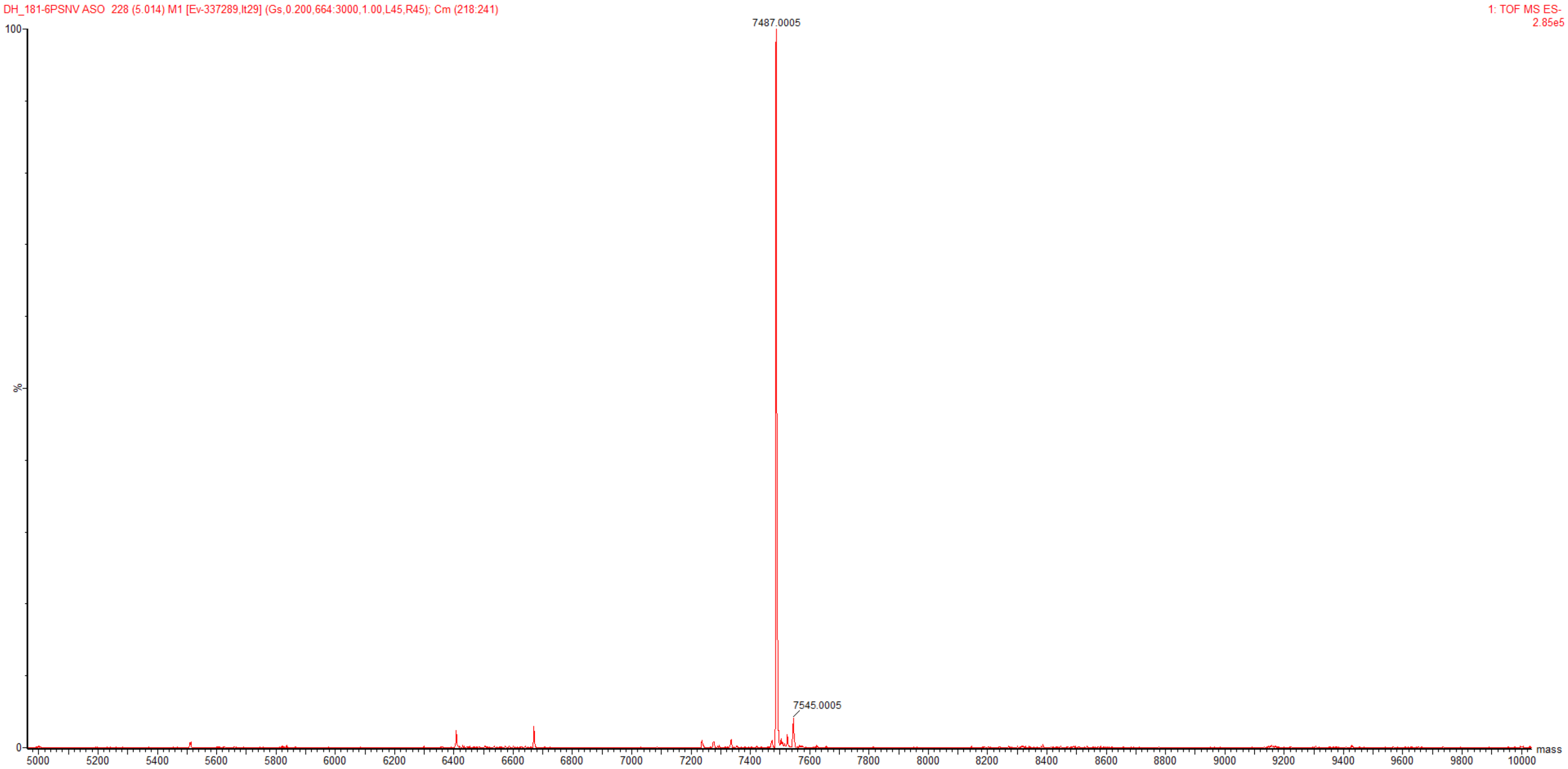
DH_181-6PSNV ASO 228 (5.014) M1 [Ev-337289,It29] (Gs,0.200,664:3000,1.00,L45,R45); Cm (218:241)
1: TOF MS ES-
2.85e5
7487.0005
7545.0005
100
%
0
mass
5000
5200
5400
5600
5800
6000
6200
6400
6600
6800
7000
7200
7400
7600
7800
8000
8200
8400
8600
8800
9000
9200
9400
9600
9800
10000

20nt-9PSNV

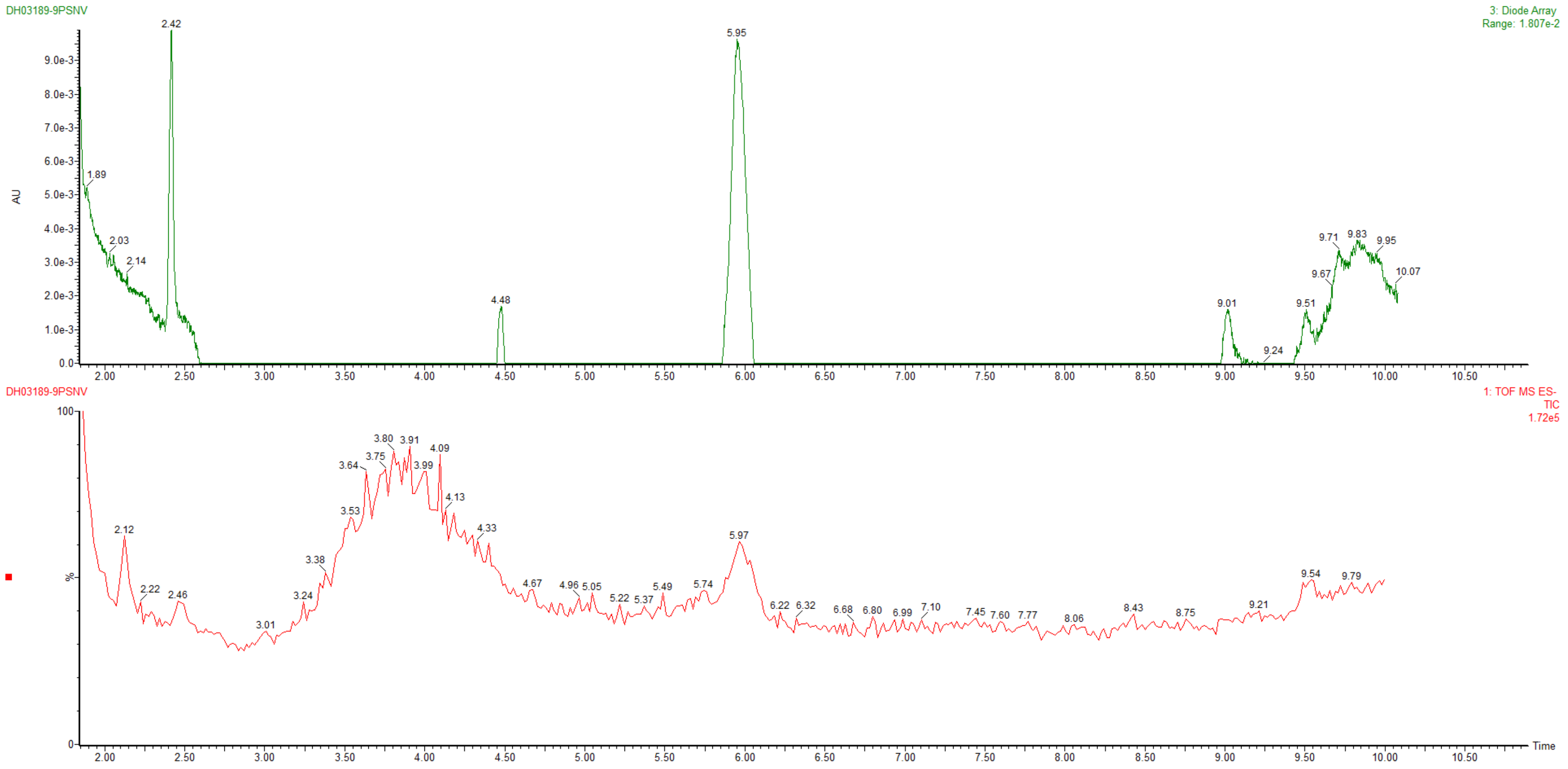
DH03189-9PSNV
3: Diode Array
Range: 1.807e-2
AU
2.42
5.95
1.89
2.03
2.14
4.48
9.01
9.24
9.51
9.67
9.71
9.83
9.95
10.07
DH03189-9PSNV
1: TOF MS ES-
TIC
1.72e5
%
2.12
2.22
2.46
3.01
3.24
3.38
3.53
3.64
3.75
3.80
3.91
3.99
4.09
4.13
4.33
4.67
4.96
5.05
5.22
5.37
5.49
5.74
5.97
6.22
6.32
6.68
6.80
6.99
7.10
7.45
7.60
7.77
8.06
8.43
8.75
9.21
9.54
9.79
Time

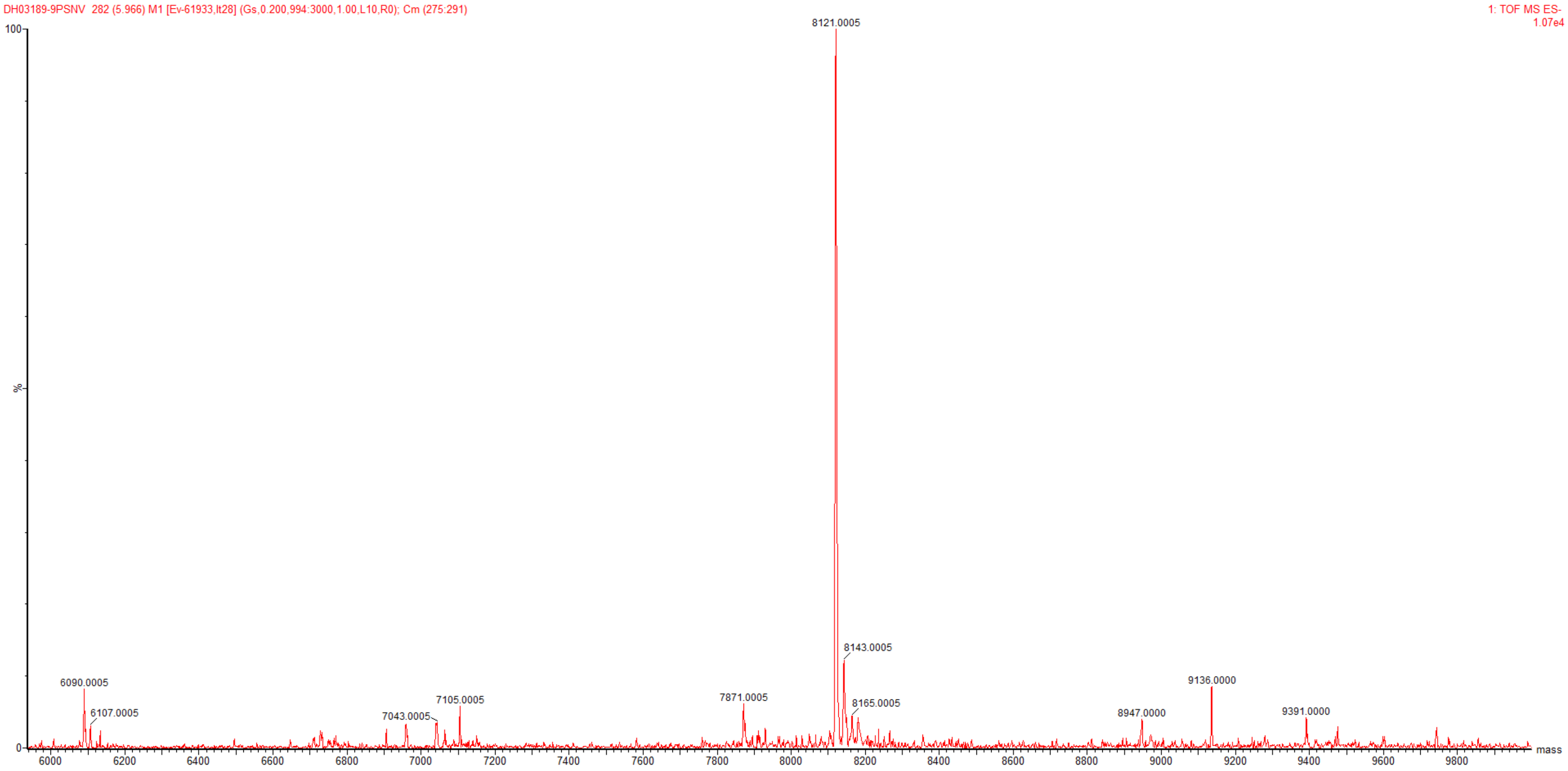
DH03189-9PSNV 282 (5.966) M1 [Ev-61933,It28] (Gs,0.200,994:3000,1.00,L10,R0); Cm (275:291)
1: TOF MS ES-
1.07e4
8121.0005
8143.0005
8165.0005
7871.0005
9136.0000
9391.0000
8947.0000
7105.0005
7043.0005
6090.0005
6107.0005
100
%
0
6000
6200
6400
6600
6800
7000
7200
7400
7600
7800
8000
8200
8400
8600
8800
9000
9200
9400
9600
9800
mass

TxRd-20nt-6PSNV

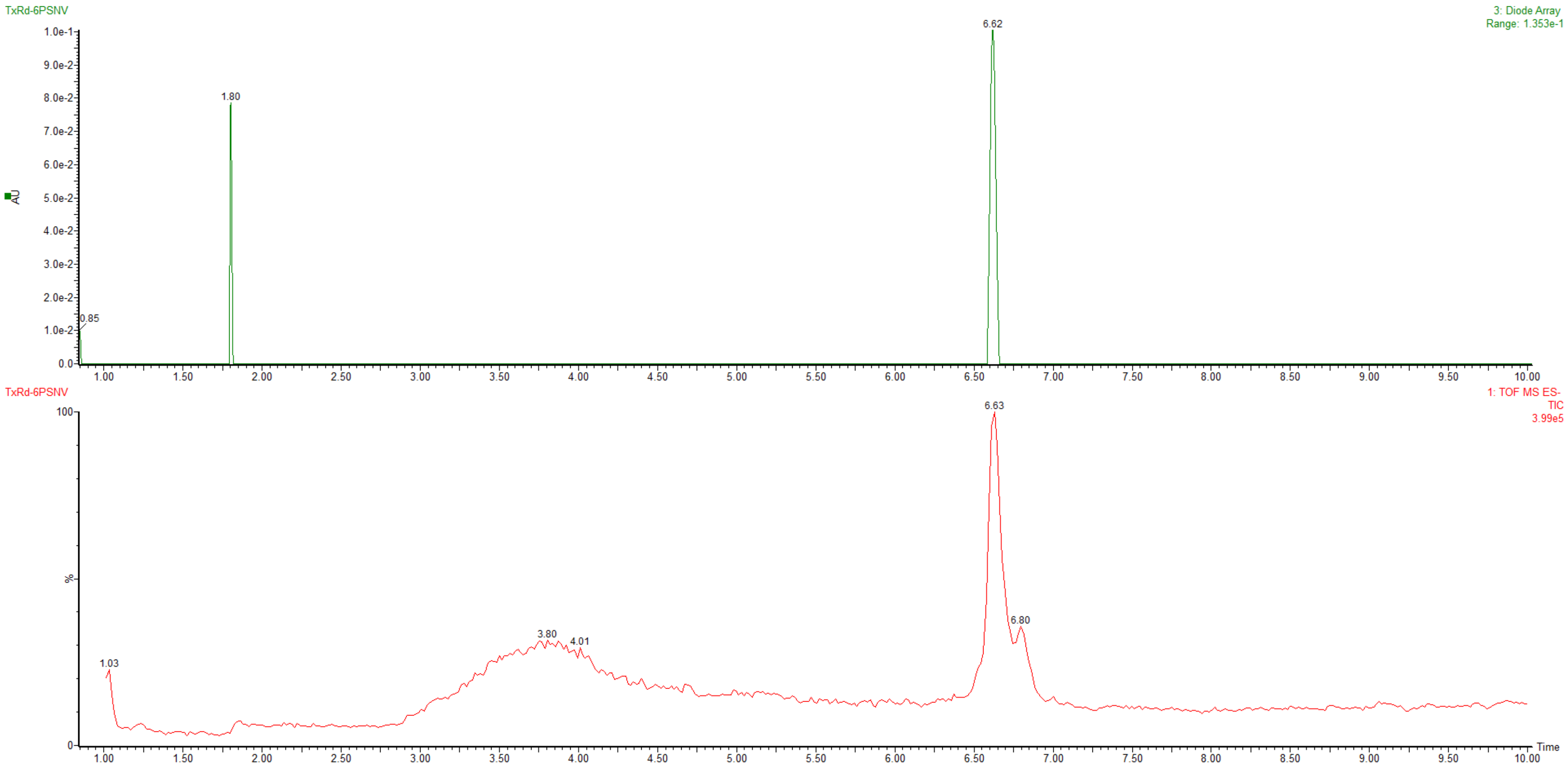

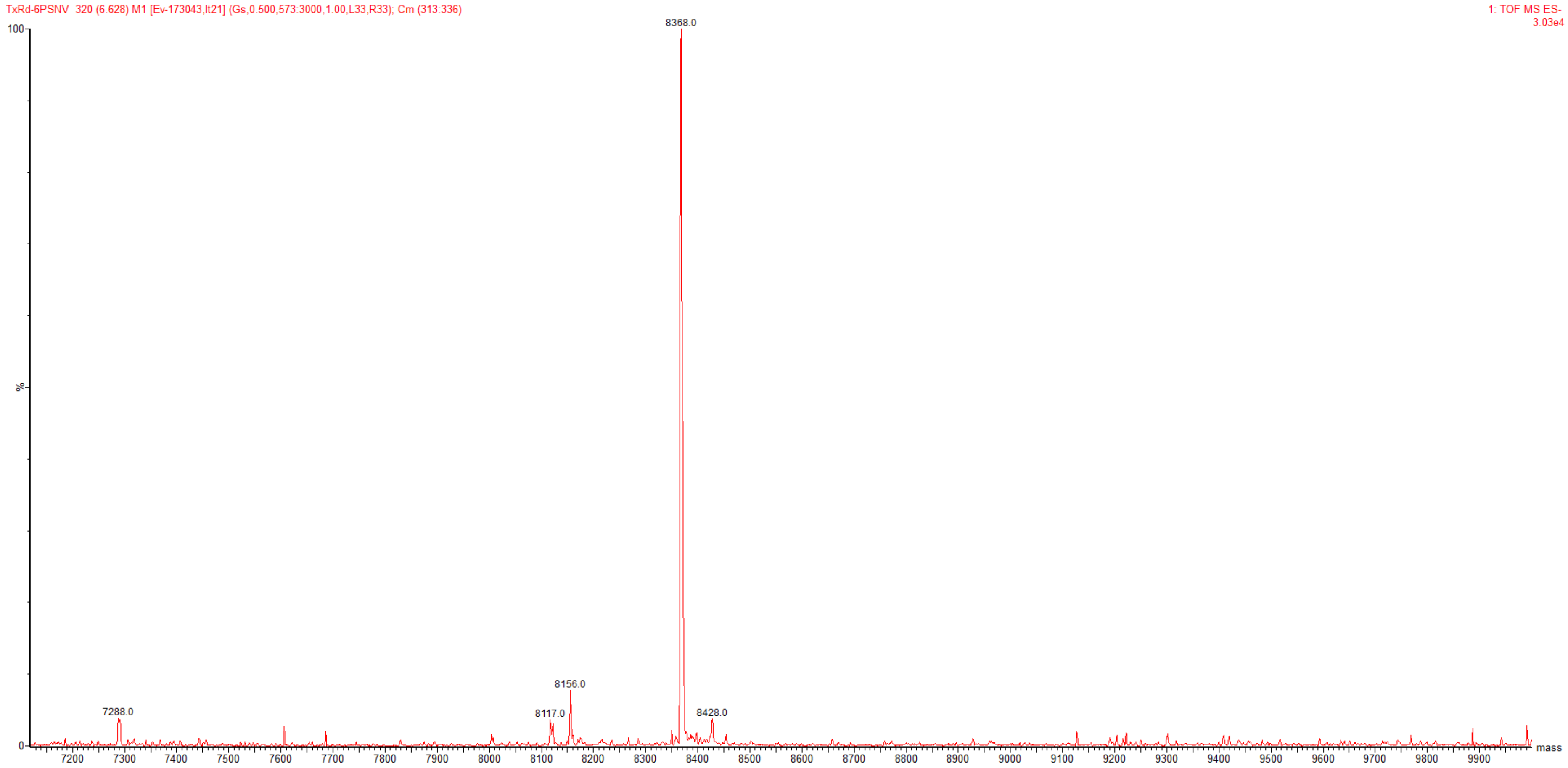
TxRd-6PSNV 320 (6.628) M1 [Ev-173043,It21] (Gs,0.500,573:3000,1.00,L33,R33); Cm (313:336)
1: TOF MS ES-
3.03e4
8368.0
8156.0
8117.0
7288.0
8428.0
100
%
0
mass
7200
7300
7400
7500
7600
7700
7800
7900
8000
8100
8200
8300
8400
8500
8600
8700
8800
8900
9000
9100
9200
9300
9400
9500
9600
9700
9800
9900

TxRd-20nt-9PSNV

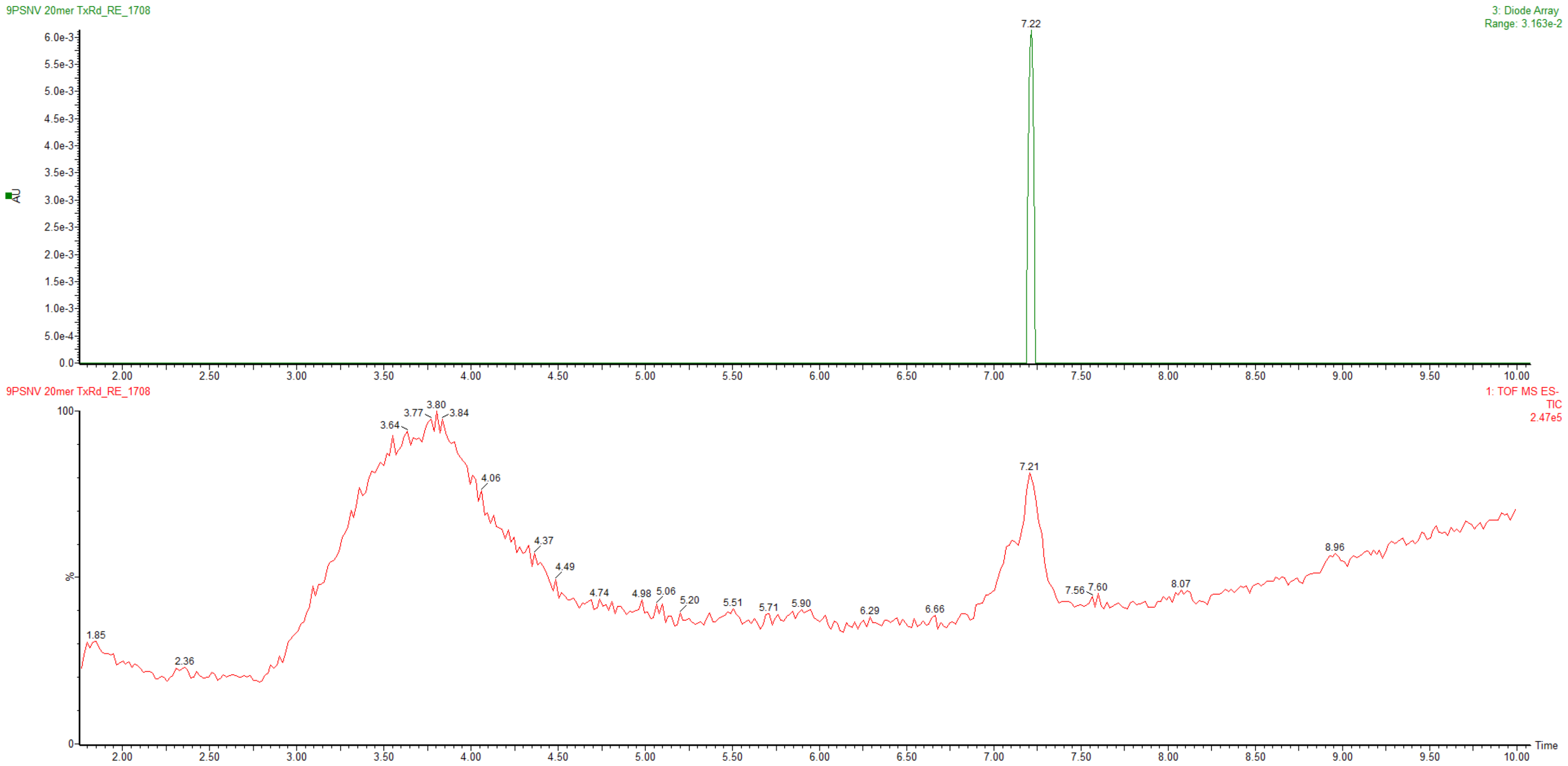

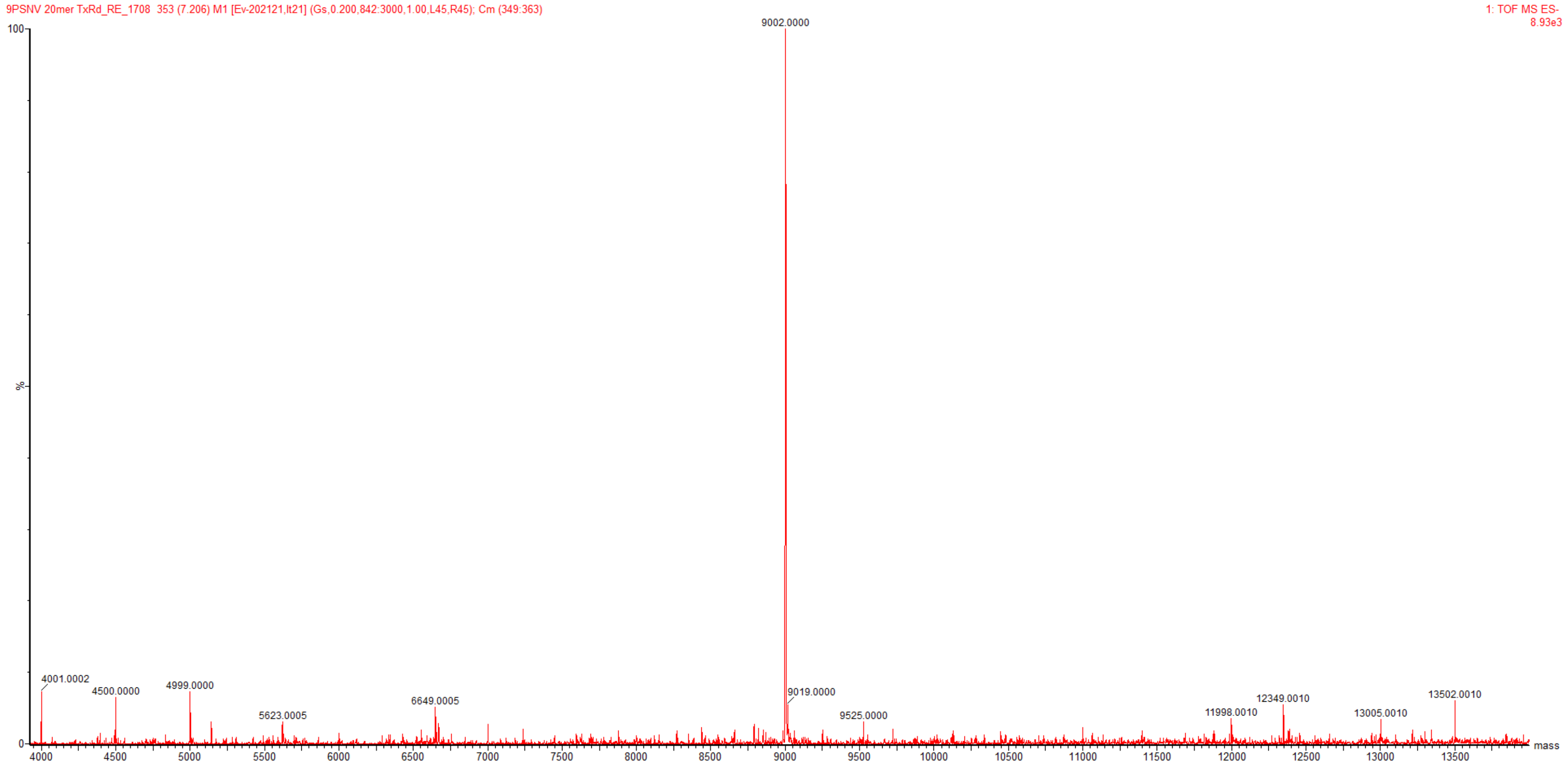
9PSNV 20mer TxRd_RE_1708 353 (7.206) M1 [Ev-202121,It21] (Gs,0.200,842:3000,1.00,L45,R45); Cm (349:363)
1: TOF MS ES-
8.93e3
100
%
0
9002.0000
4001.0002
4500.0000
4999.0000
5623.0005
6649.0005
9019.0000
9525.0000
11998.0010
12349.0010
13005.0010
13502.0010
4000
4500
5000
5500
6000
6500
7000
7500
8000
8500
9000
9500
10000
10500
11000
11500
12000
12500
13000
13500
mass

TxRd-14nt-6PSNV

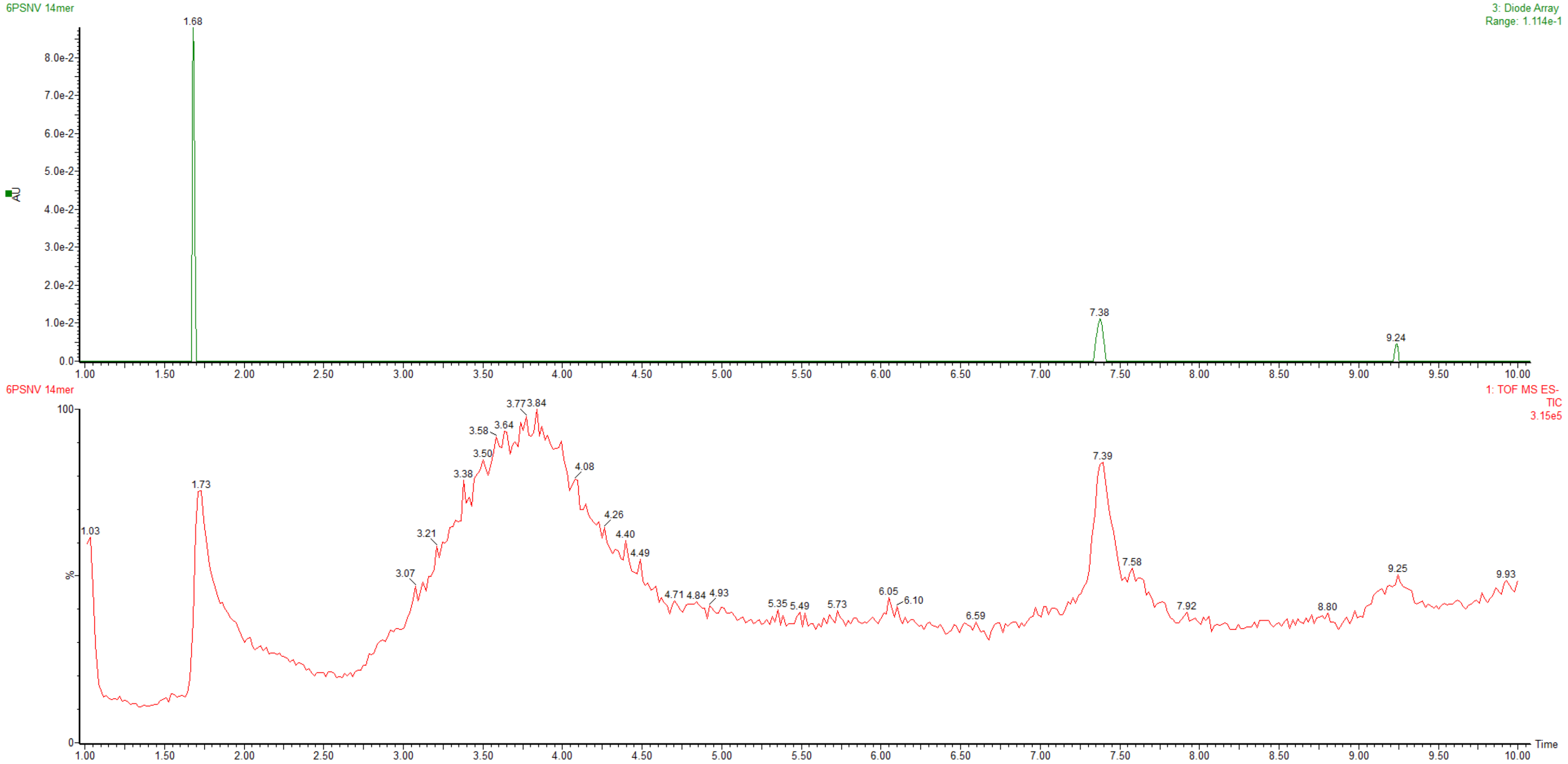

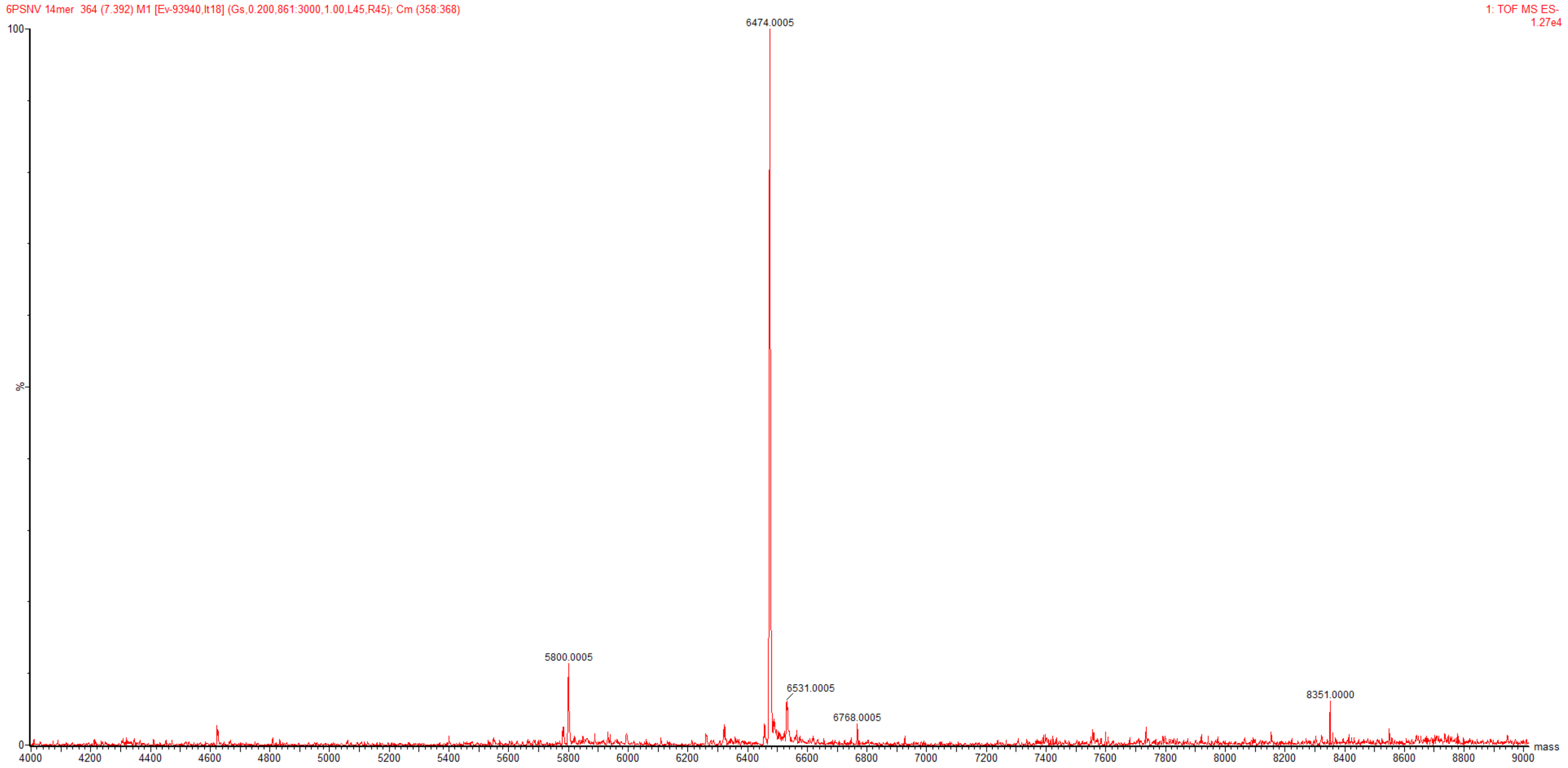

6PSNV 14mer 364 (7.392) M1 [Ev-93940,It18] (Gs,0.200,861:3000,1.00,L45,R45); Cm (358:368)
1: TOF MS ES-
1.27e4
6474.0005
5800.0005
6531.0005
6768.0005
8351.0000
100
%
0
mass
4000
4200
4400
4600
4800
5000
5200
5400
5600
5800
6000
6200
6400
6600
6800
7000
7200
7400
7600
7800
8000
8200
8400
8600
8800
9000

Blank

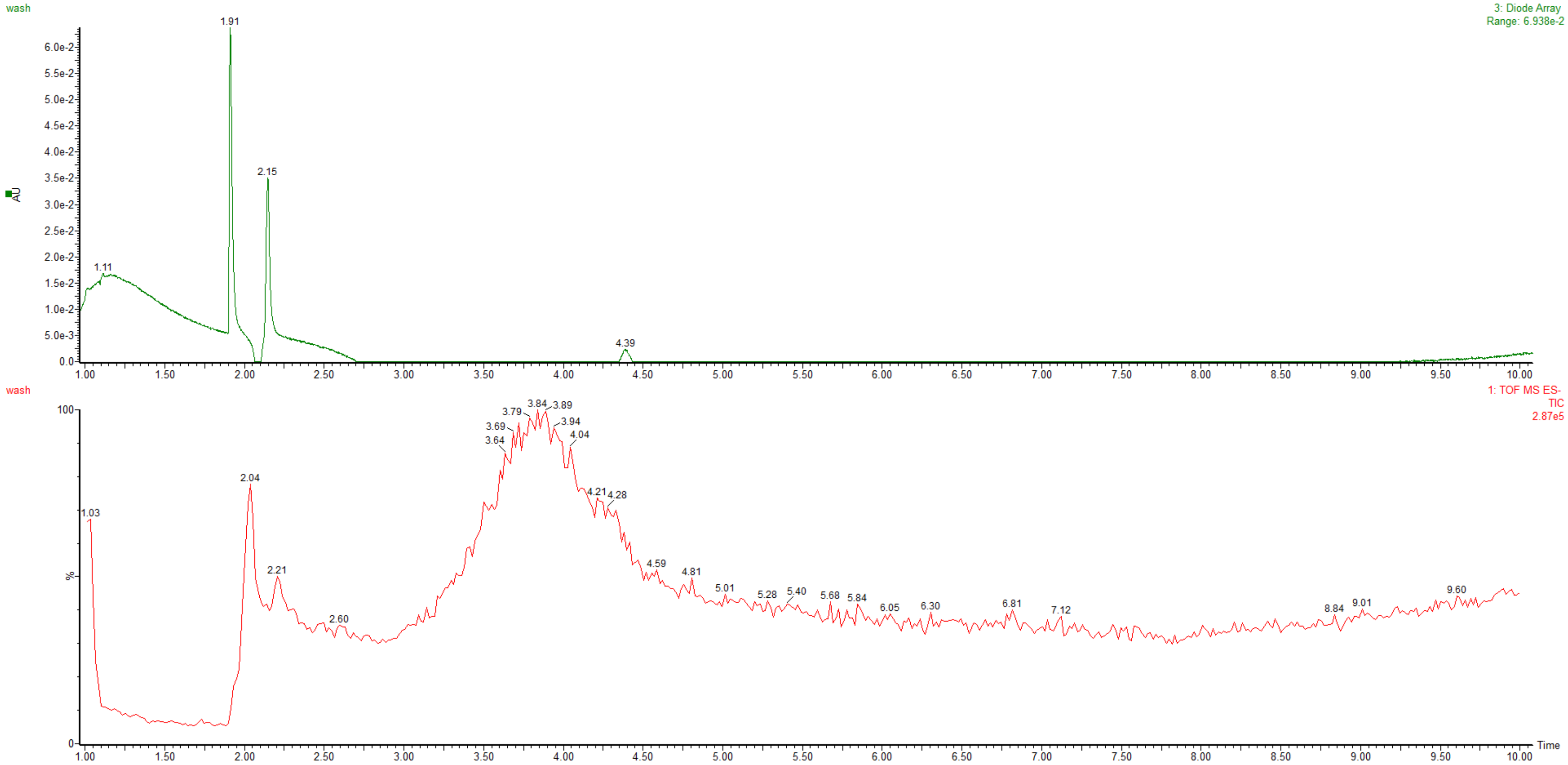
wash
3: Diode Array
Range: 6.938e-2
AU
1.91
2.15
1.11
4.39
wash
1: TOF MS ES-
TIC
2.87e5
%
1.03
2.04
2.21
2.60
3.64
3.69
3.79
3.84
3.89
3.94
4.04
4.21
4.28
4.59
4.81
5.01
5.28
5.40
5.68
5.84
6.05
6.30
6.81
7.12
8.84
9.01
9.60
Time

NMR Spectra

S-(4,5-Dimethoxy-2-nitrobenzyl) *O,O*-diethyl phosphorothioate

^{1}H-NMR (600 Hz, $CDCl_3$)

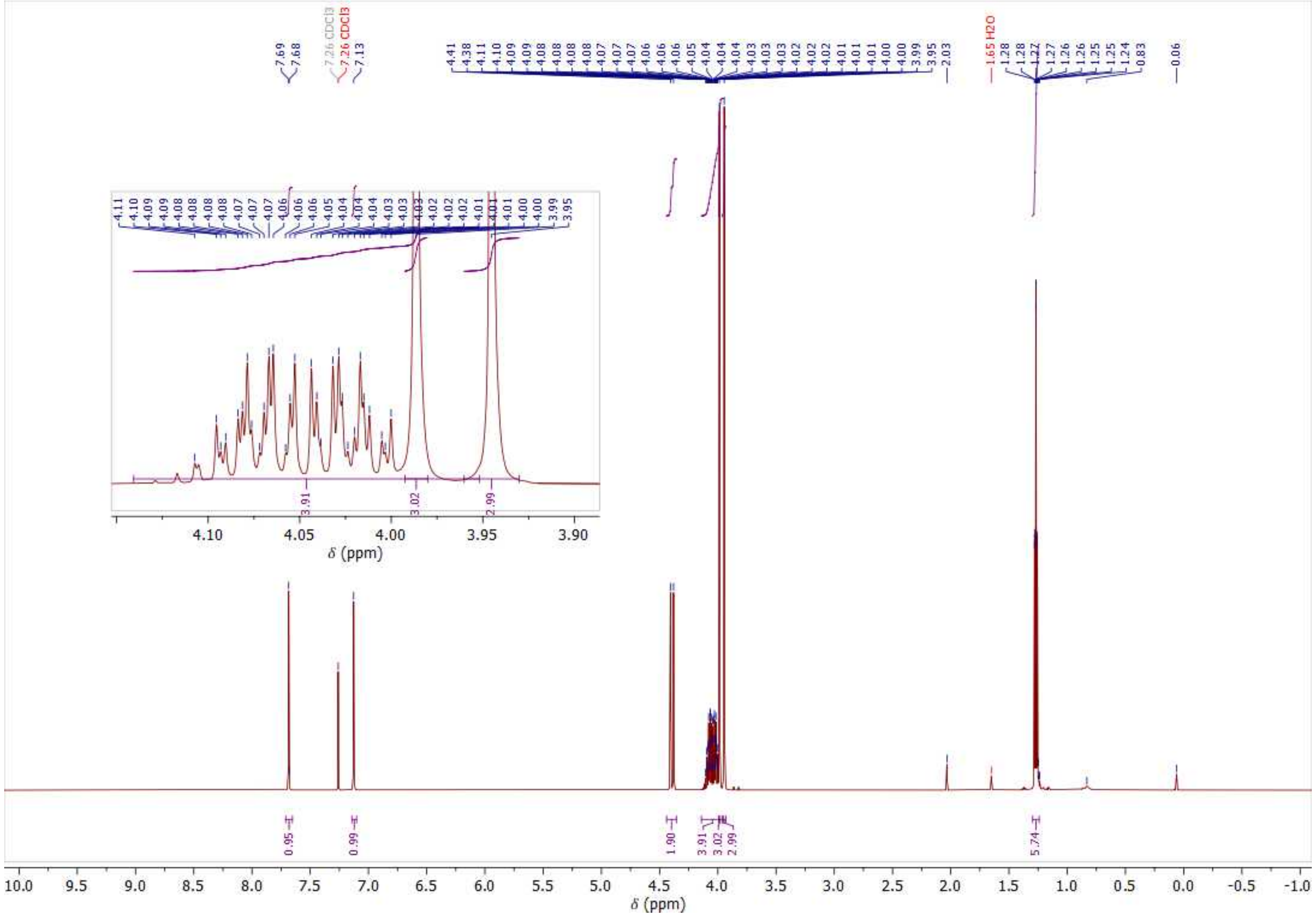

$^{13}C\{^1H\}$-NMR (151 Hz, $CDCl_3$)

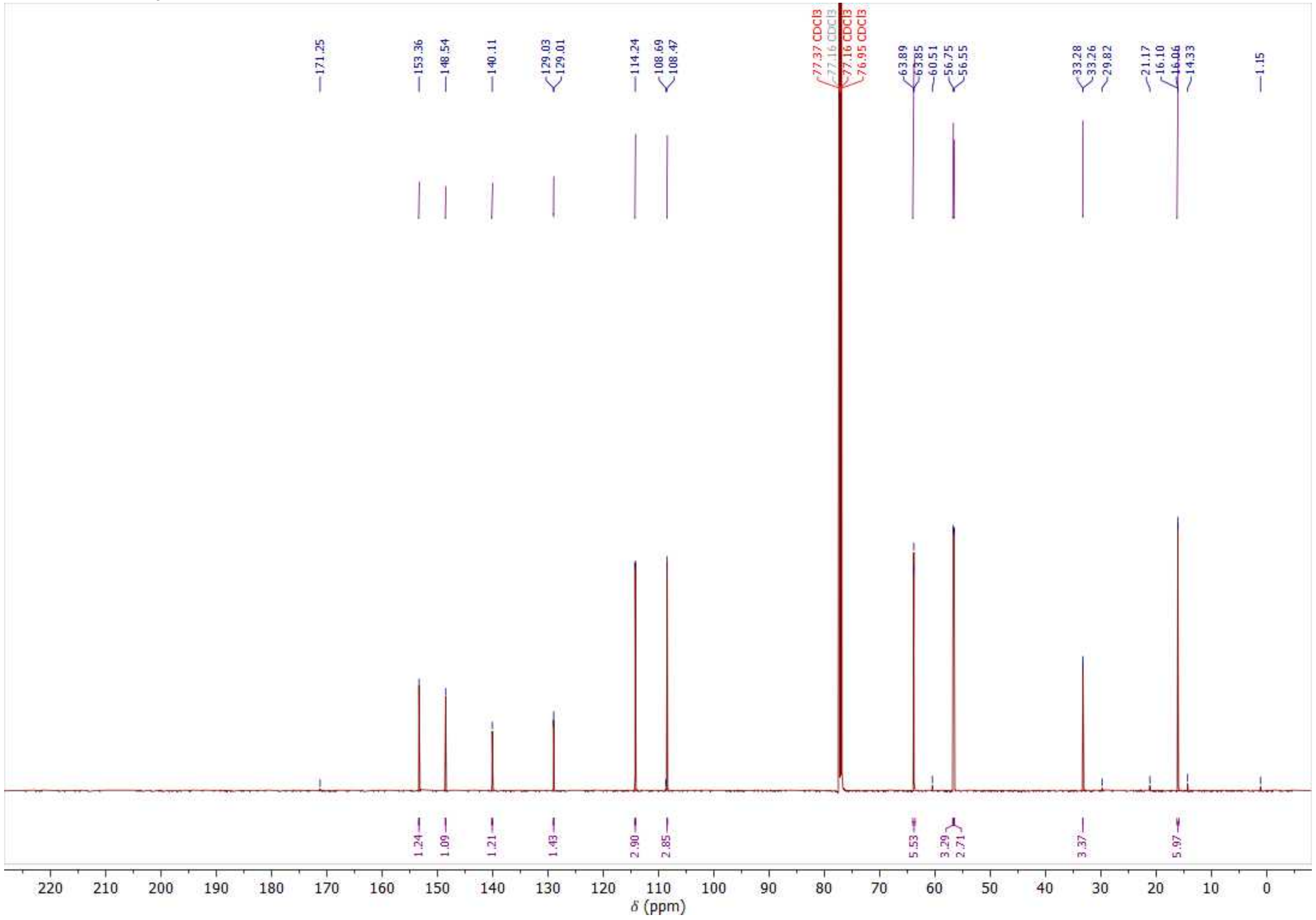

^{31}P-NMR (243 MHz, $CDCl_3$)

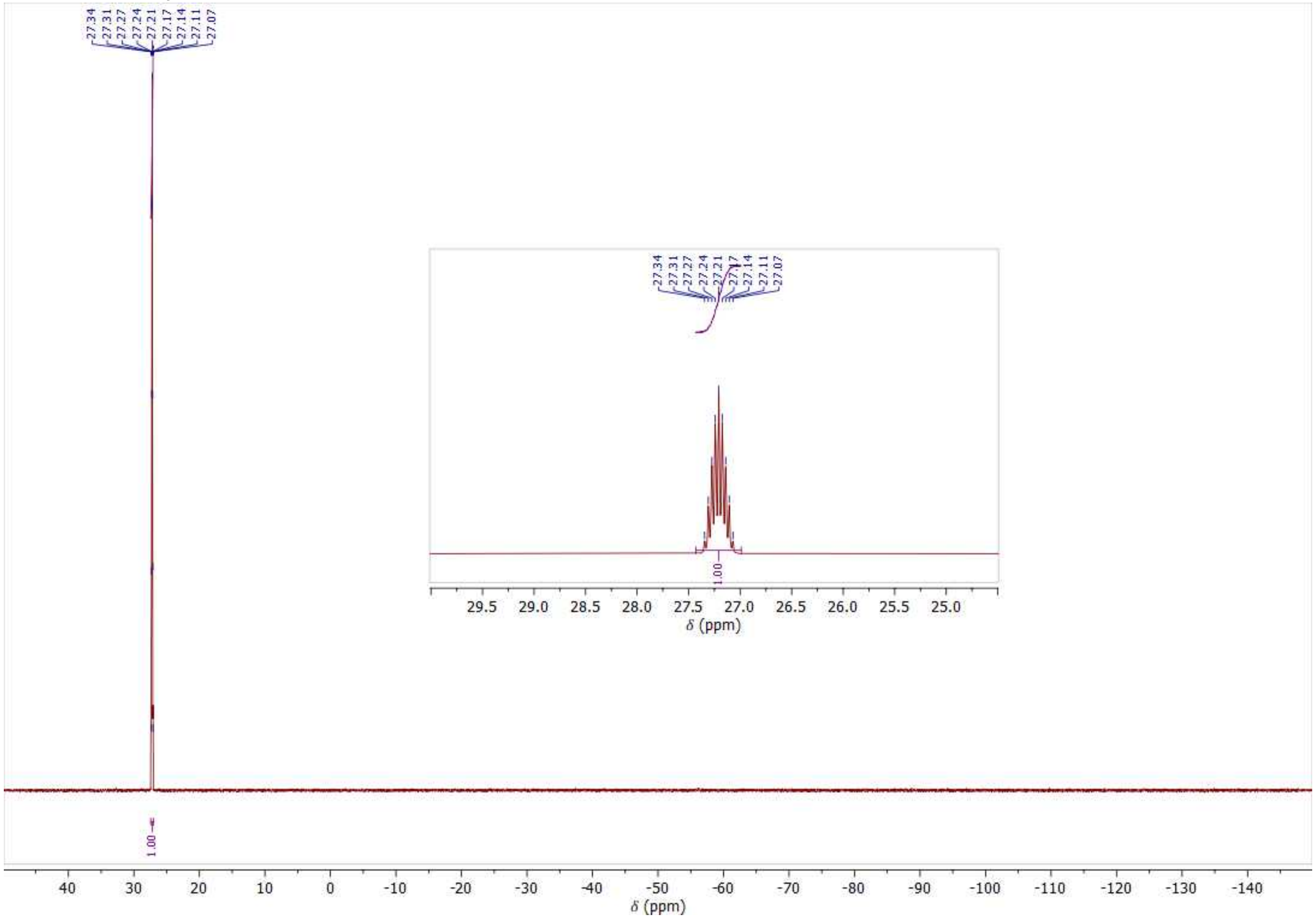

References

(1) Booth, M. J.; Restrepo Schild, V.; Graham, A. D.; Olof, S. N.; Bayley, H. Light-Activated Communication in Synthetic Tissues. *Science Advances* **2016**, *2* (4), e1600056. https://doi.org/10.1126/sciadv.1600056.

(2) Ding, Y.; Chan, C. Y.; Lawrence, C. E. Sfold Web Server for Statistical Folding and Rational Design of Nucleic Acids. *Nucleic Acids Research* **2004**, *32* (suppl_2), W135–W141. https://doi.org/10.1093/nar/gkh449.

(3) Epple, S.; Thorpe, C.; Baker, Y. R.; El-Sagheer, A. H.; Brown, T. Consecutive 5′- And 3′-Amide Linkages Stabilise Antisense Oligonucleotides and Elicit an Efficient RNase H Response. *Chemical Communications* **2020**, *56* (41), 5496–5499. https://doi.org/10.1039/d0cc00444h.

Uncropped and Unedited Gels

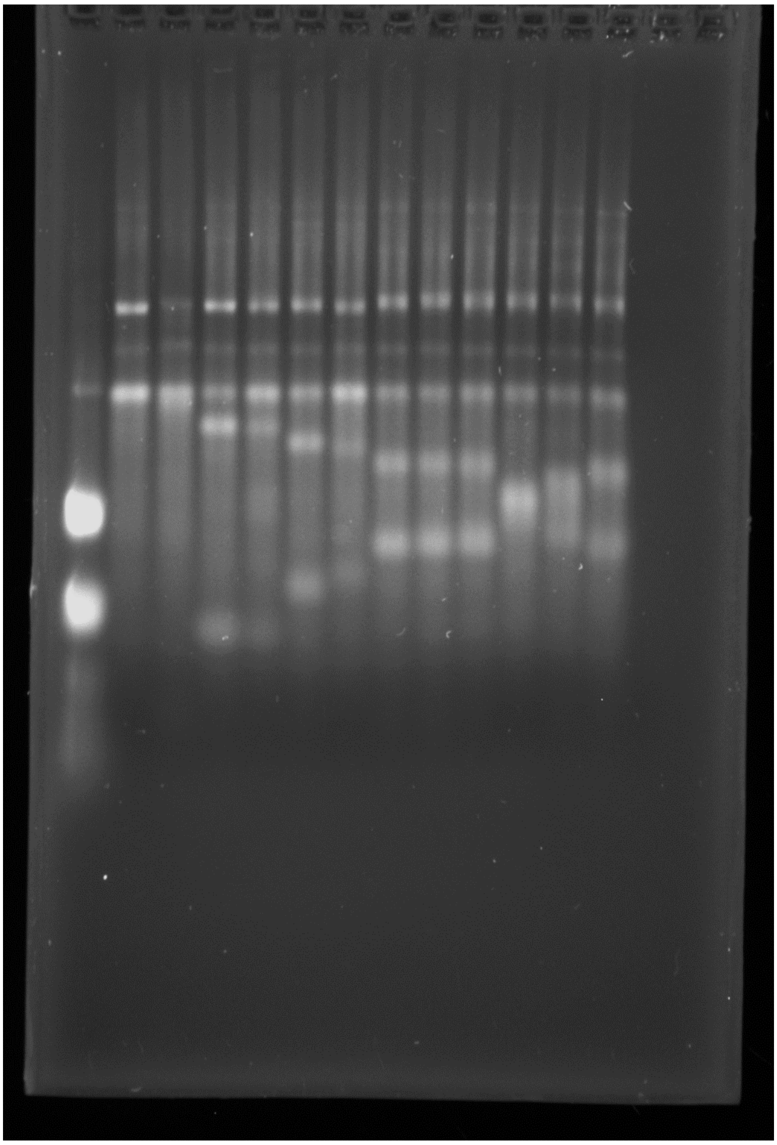

Supplementary Figure 32: Uncropped and unedited gel from **Supplementary Figure 1.**

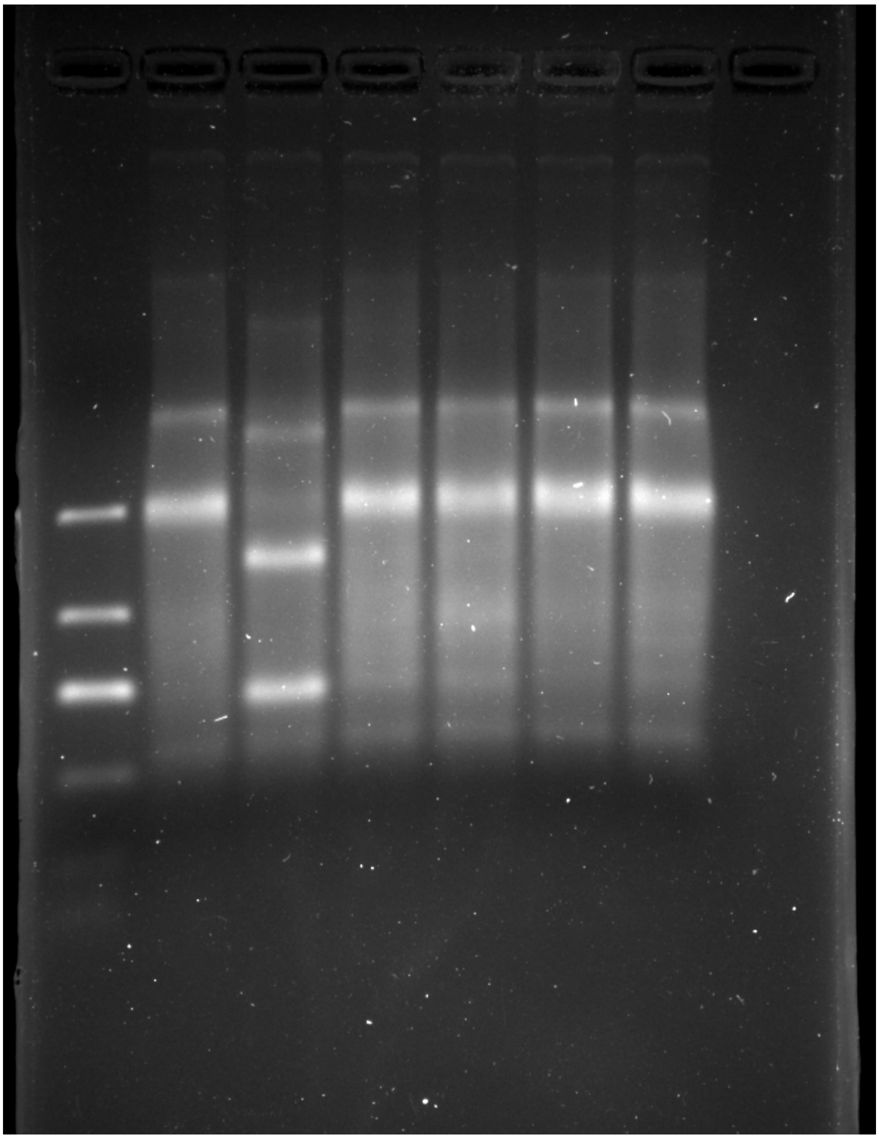

Supplementary Figure 33: Uncropped and unedited gel from **Supplementary Figure 2.**

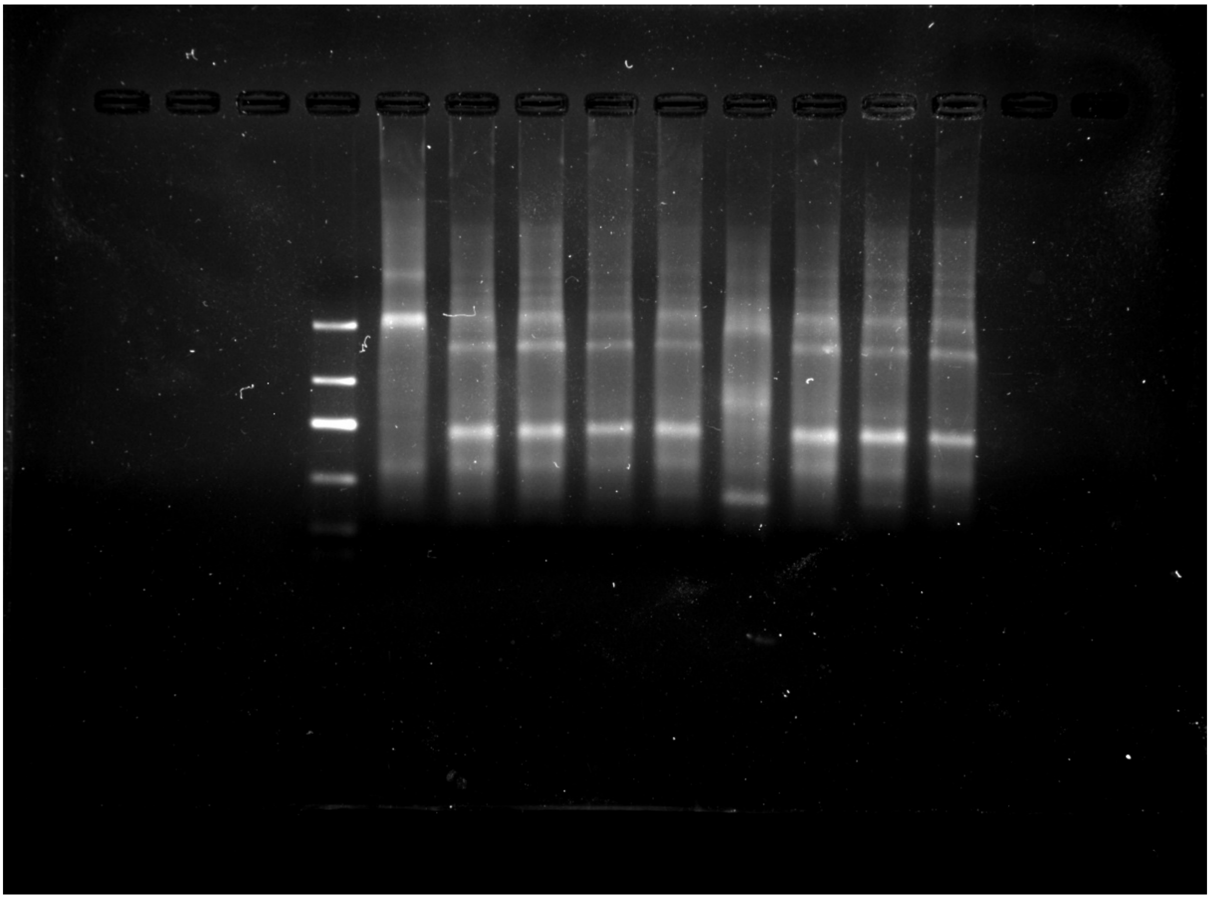

Supplementary Figure 34: Uncropped and unedited gel from **Supplementary Figure 7.**

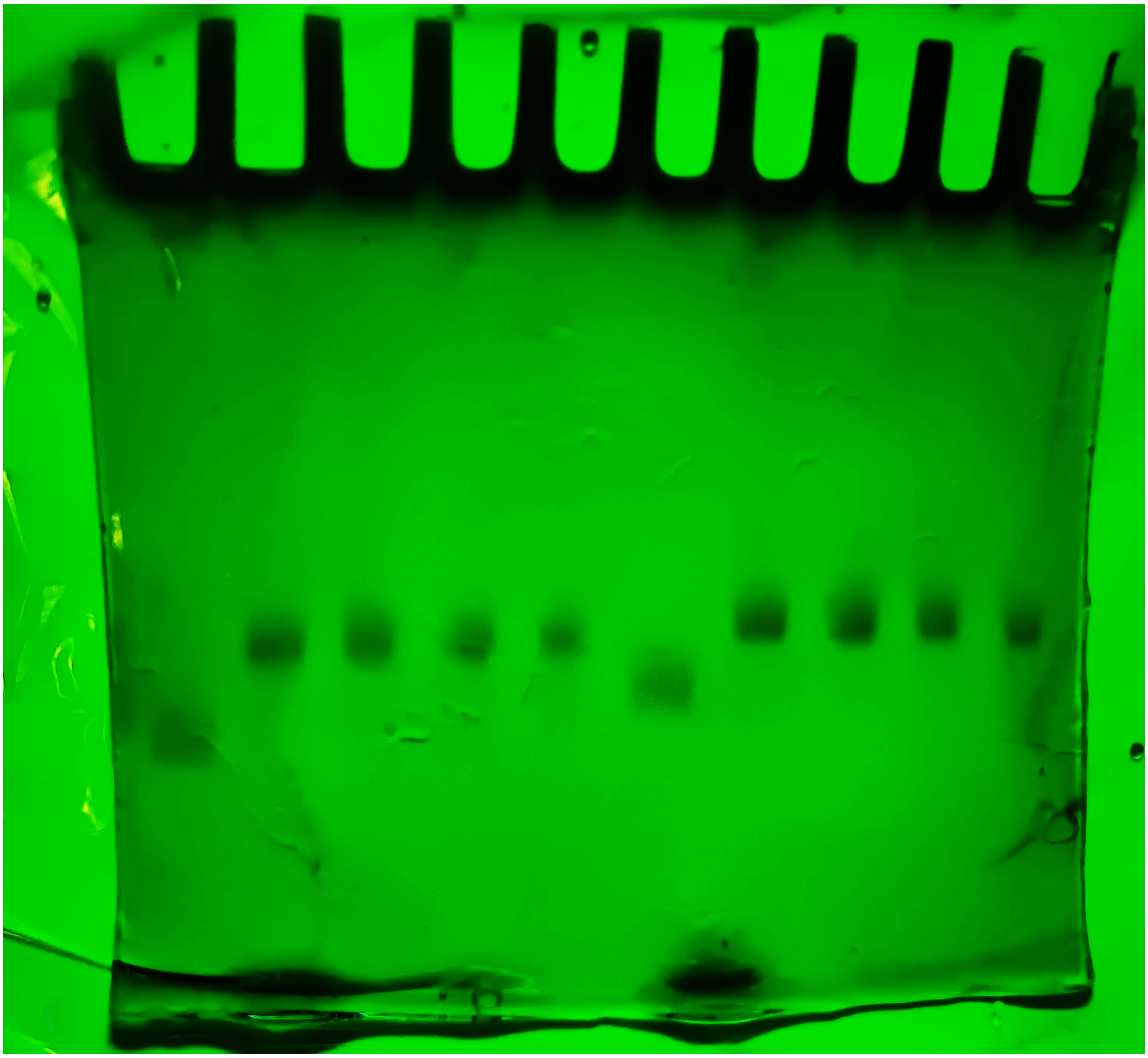

Supplementary Figure 35: Uncropped and unedited gel from **Supplementary Figure 9.**

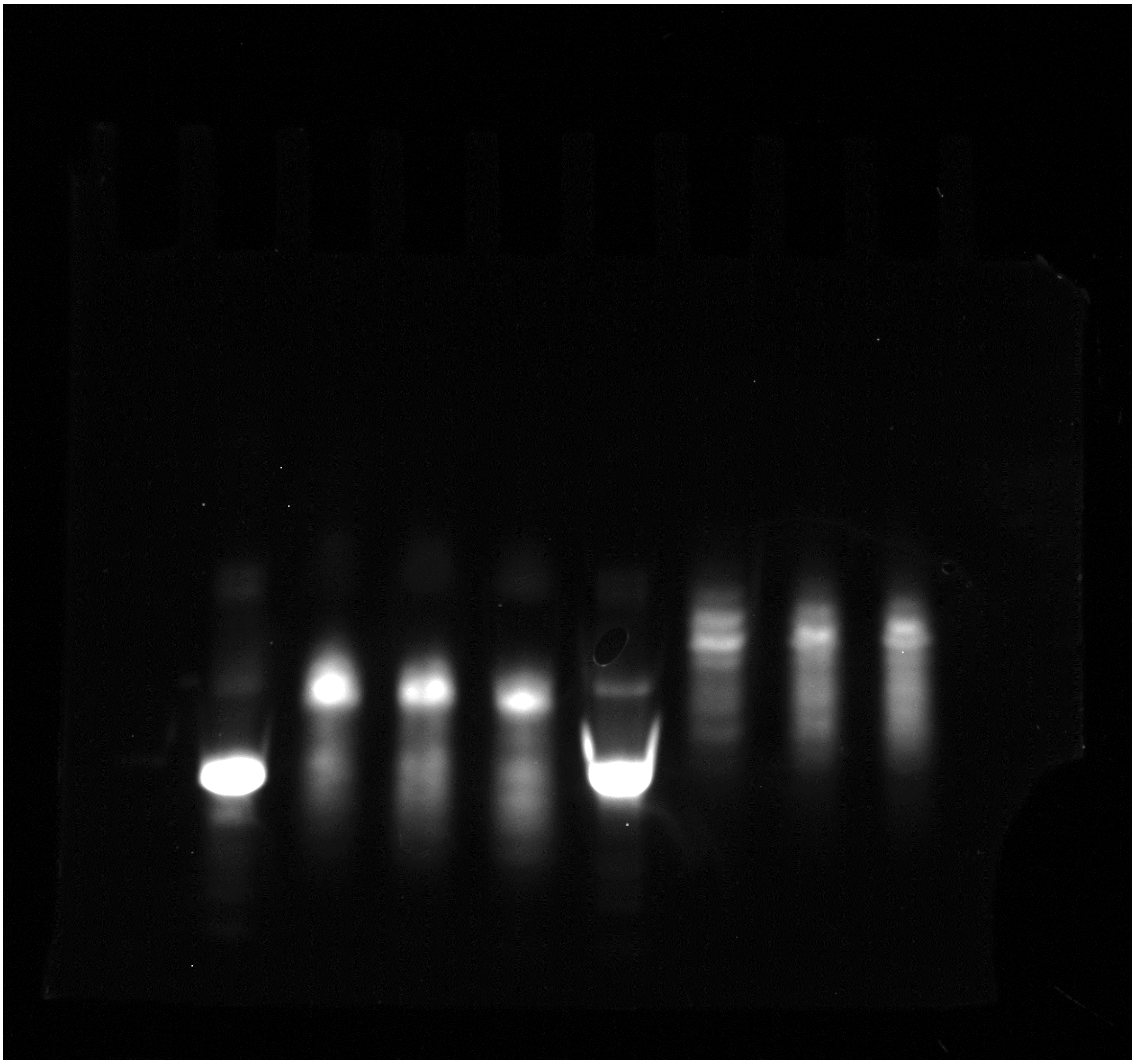

Supplementary Figure 36: Uncropped and unedited gel from **Supplementary Figure 10.**

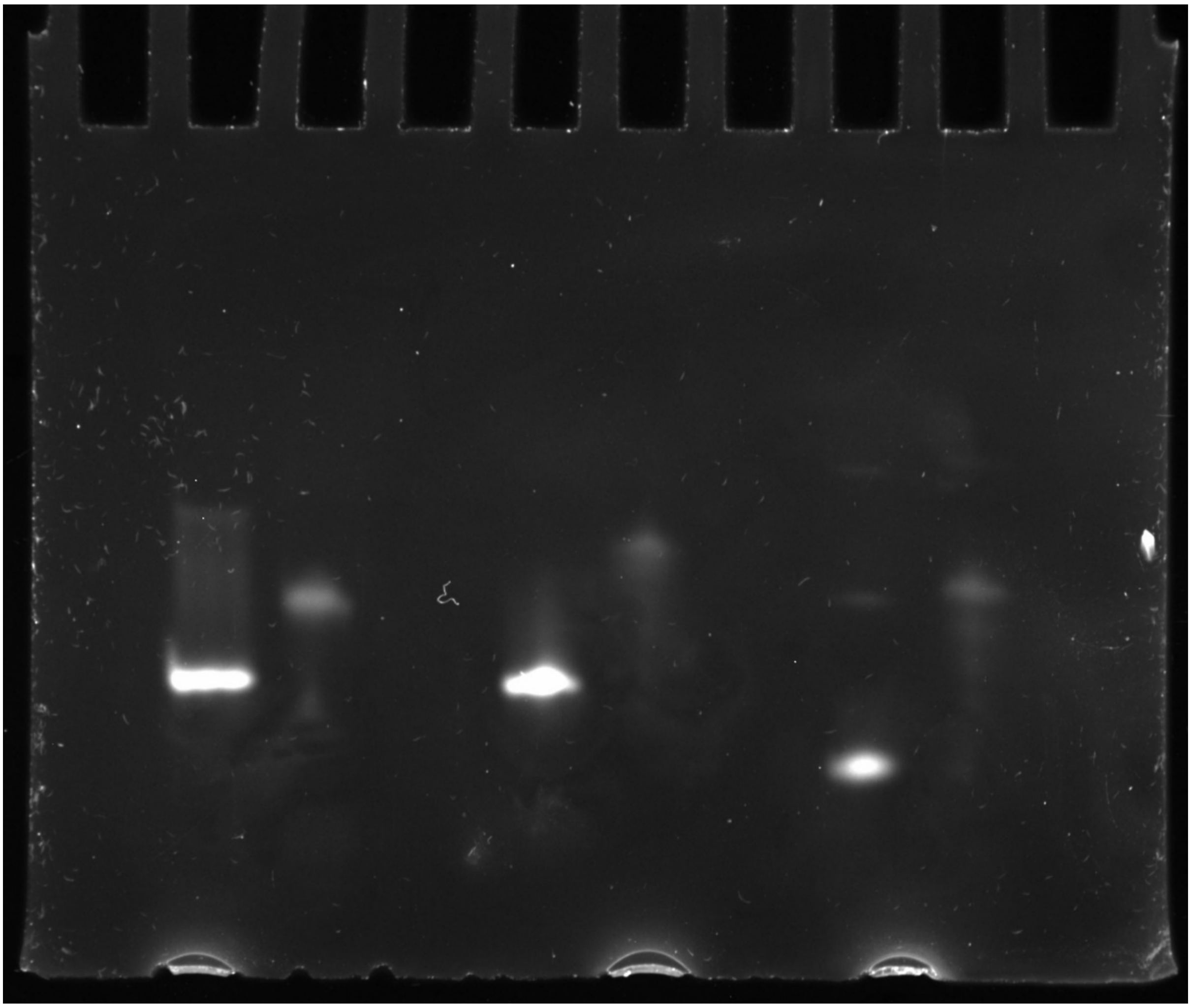

Supplementary Figure 37: Uncropped and unedited gel from **Supplementary Figure 12.**

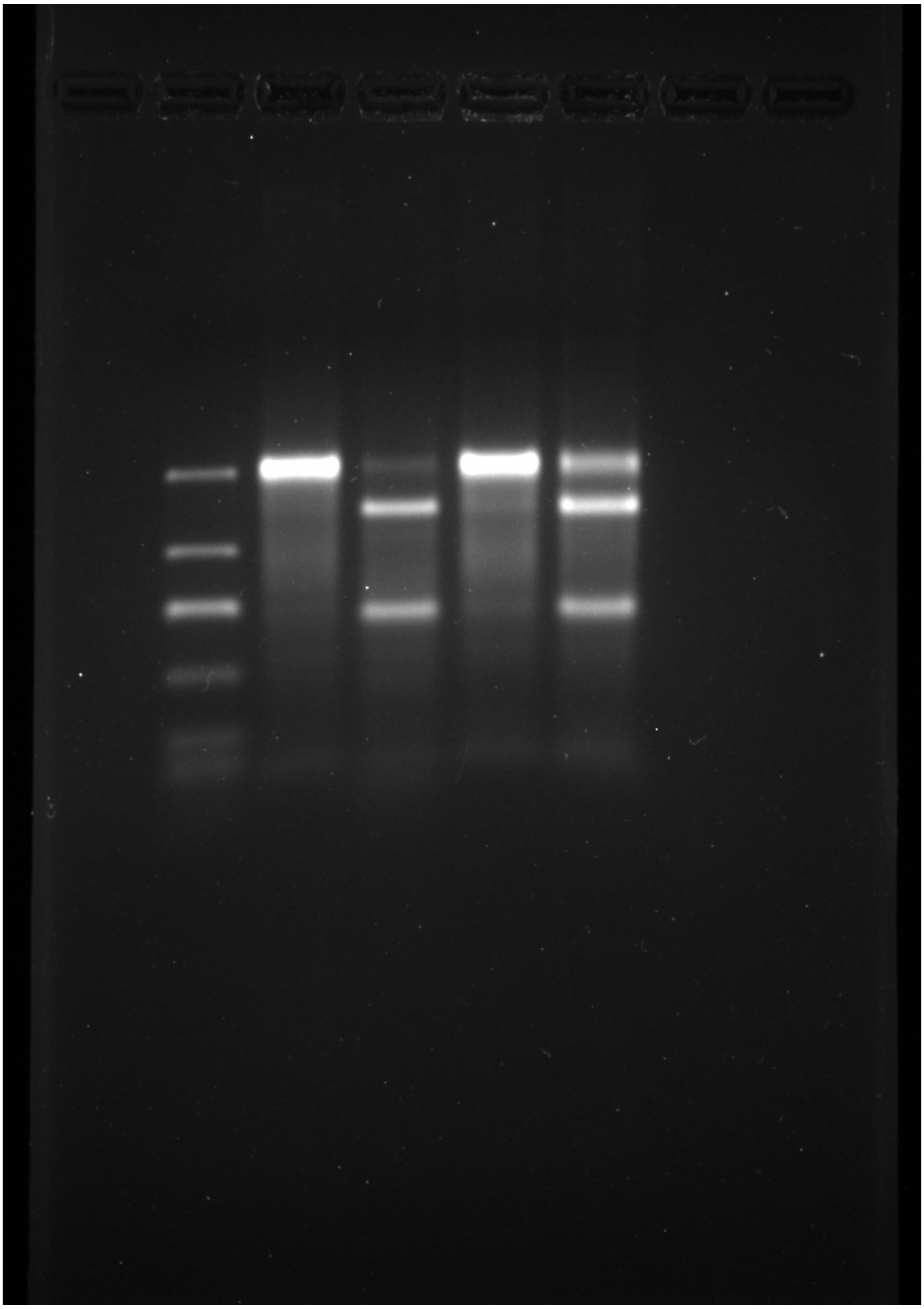

Supplementary Figure 38: Uncropped and unedited gel from **Figure 4,** a.

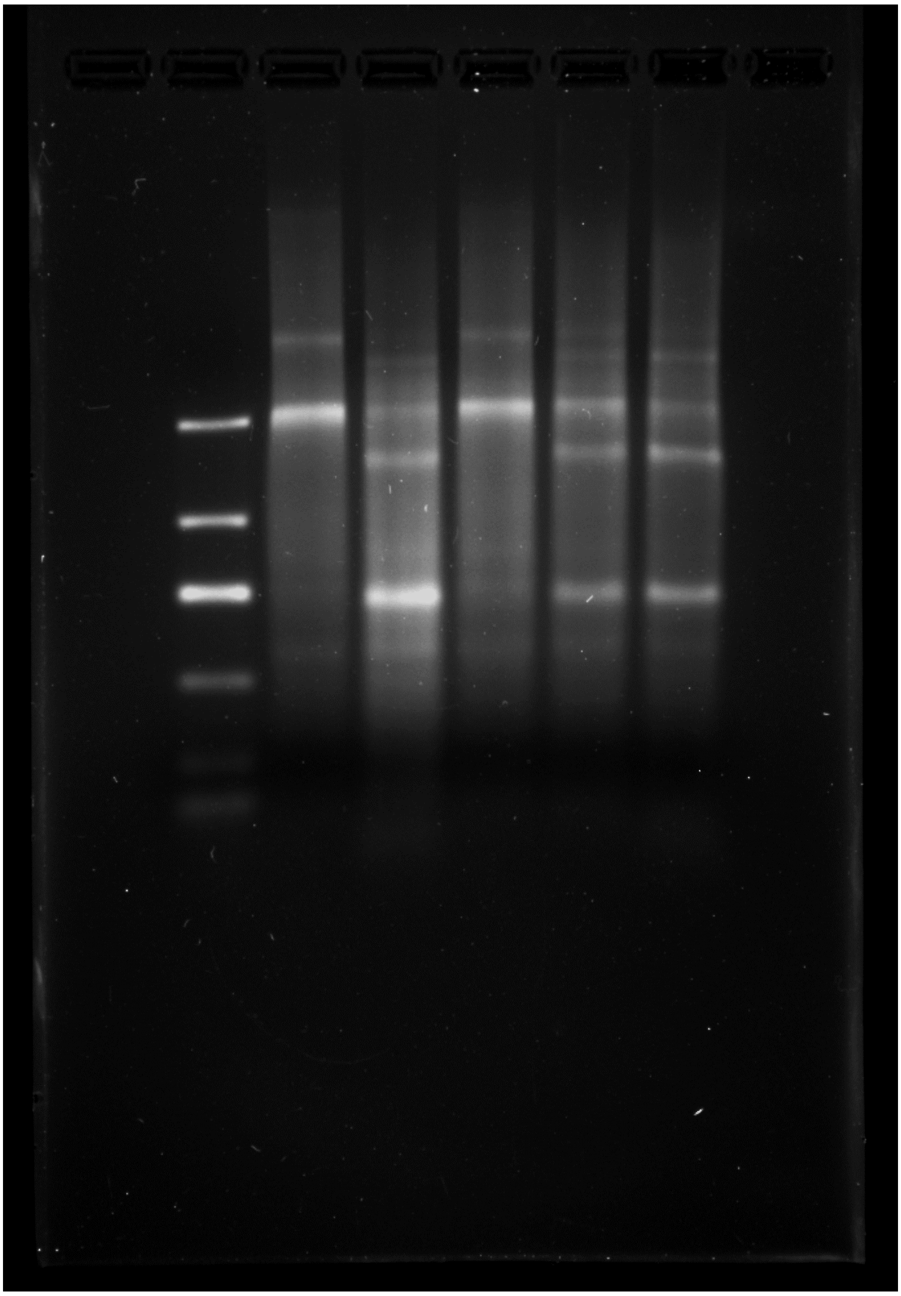

Supplementary Figure 39: Uncropped and unedited gel from **Supplementary Figure 14.**

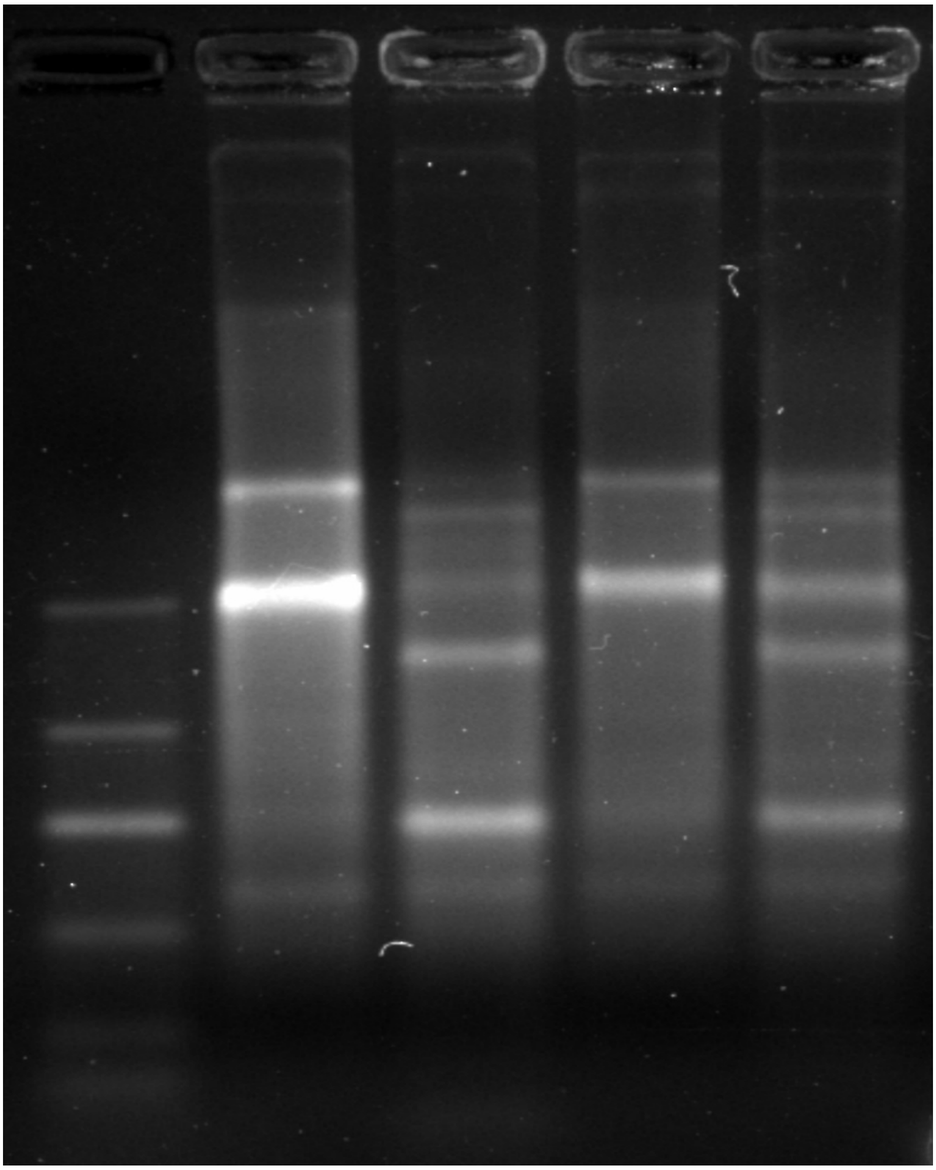

Supplementary Figure 40: Uncropped and unedited gel from **Supplementary Figure 15.**

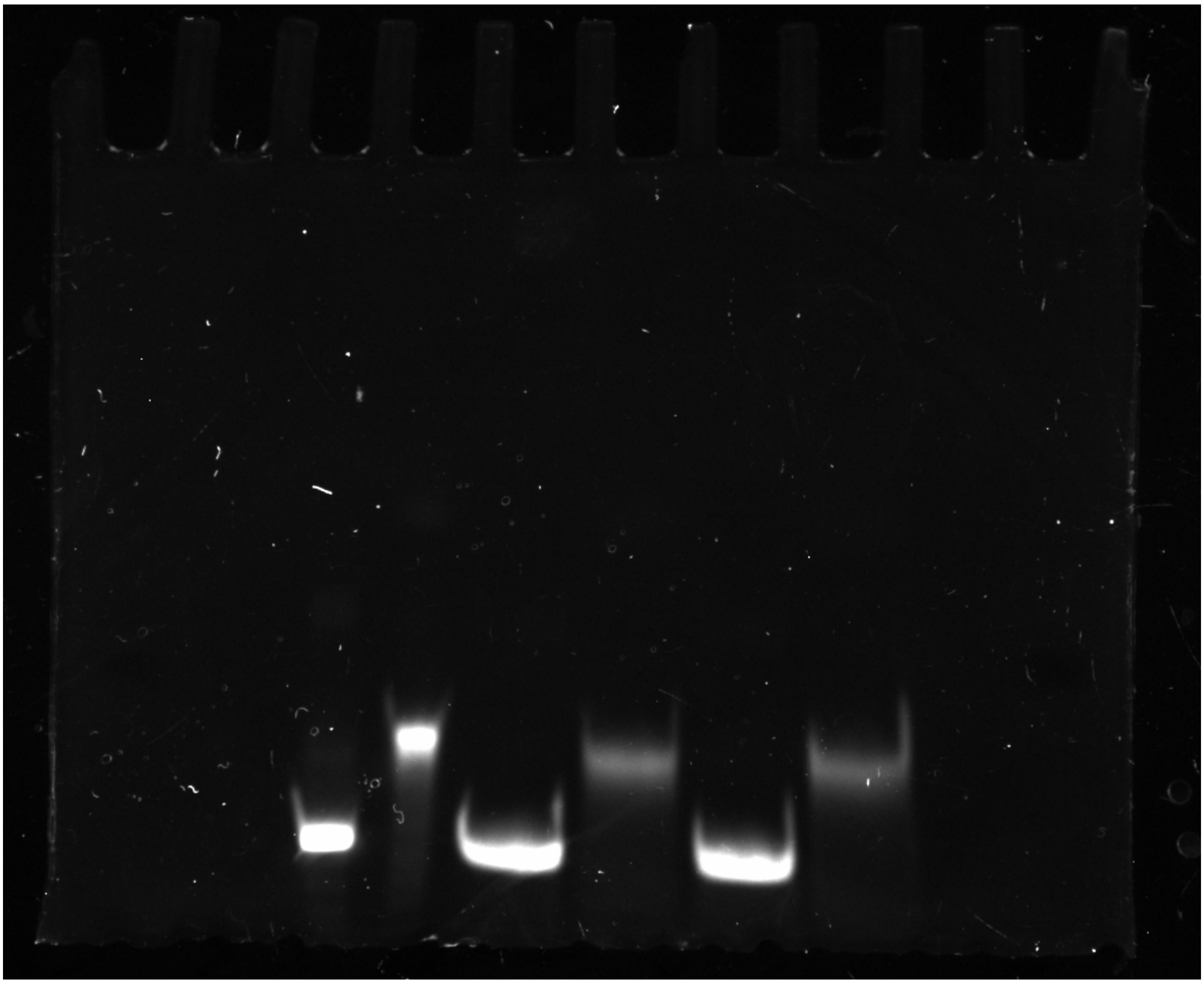

Supplementary Figure 41: Uncropped and unedited gel from **Supplementary Figure 22.**

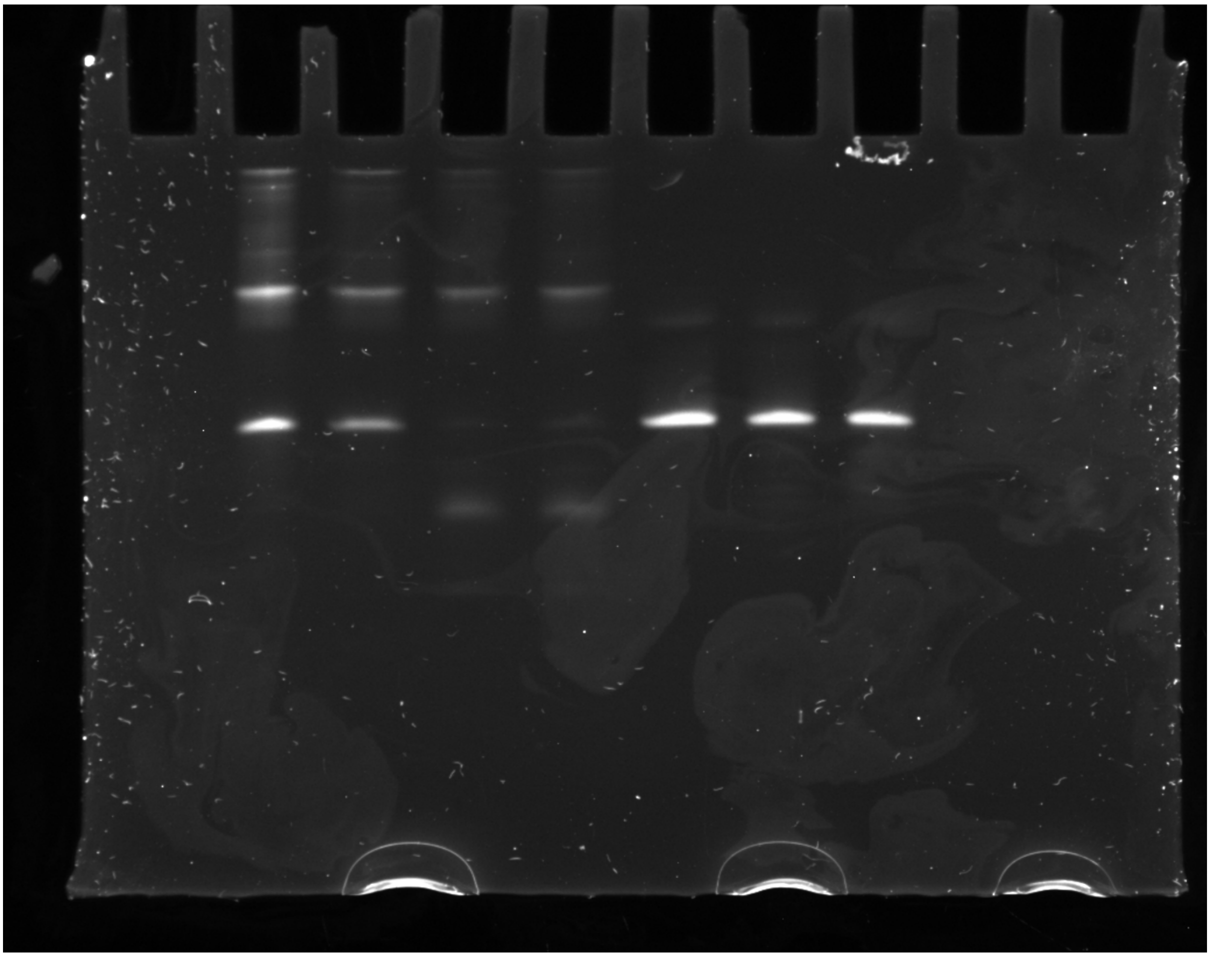

Supplementary Figure 42: Uncropped and unedited gel from **Supplementary Figure 23**, a.

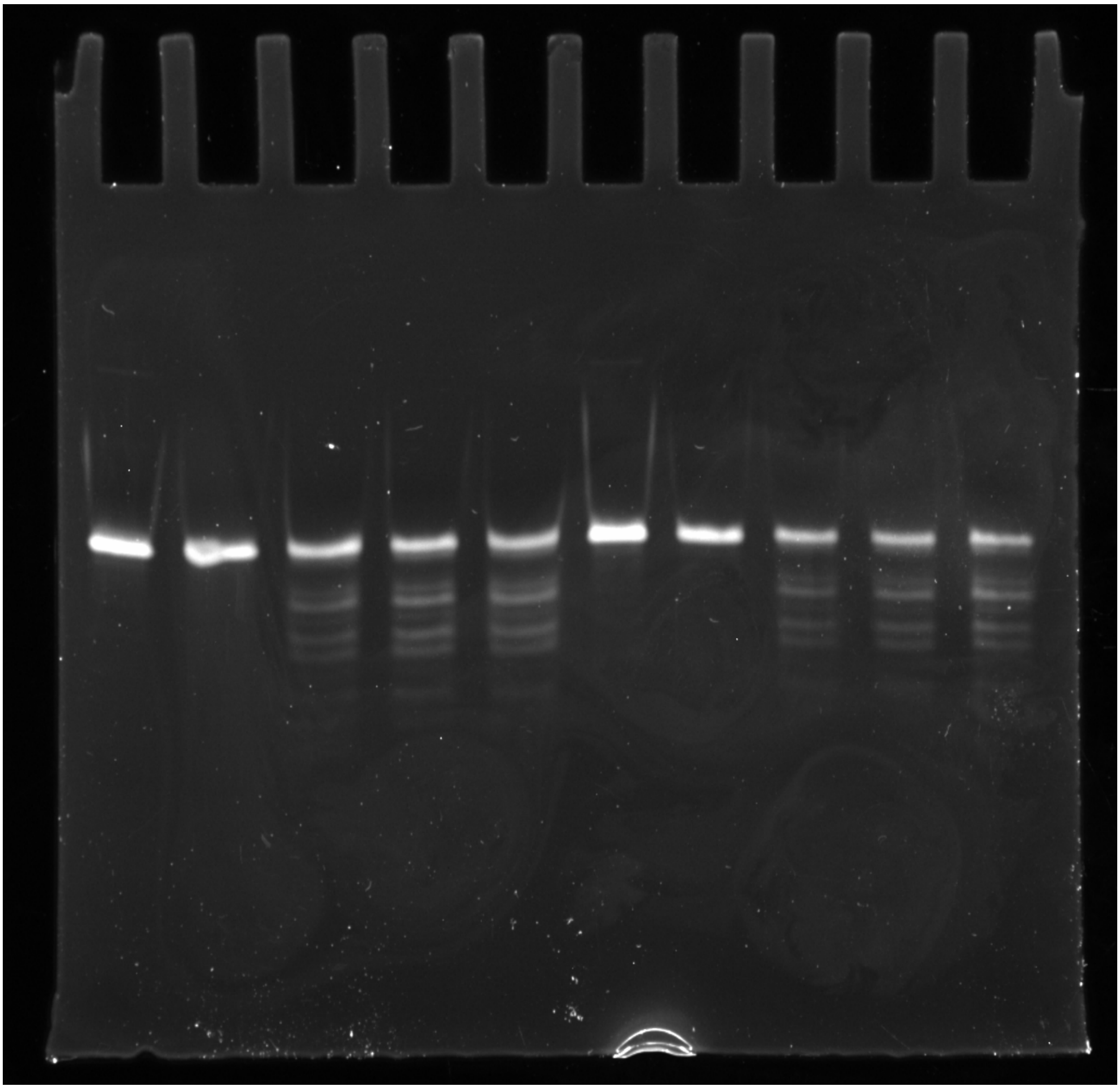

Supplementary Figure 43: Uncropped and unedited gel from **Supplementary Figure 23**, b.

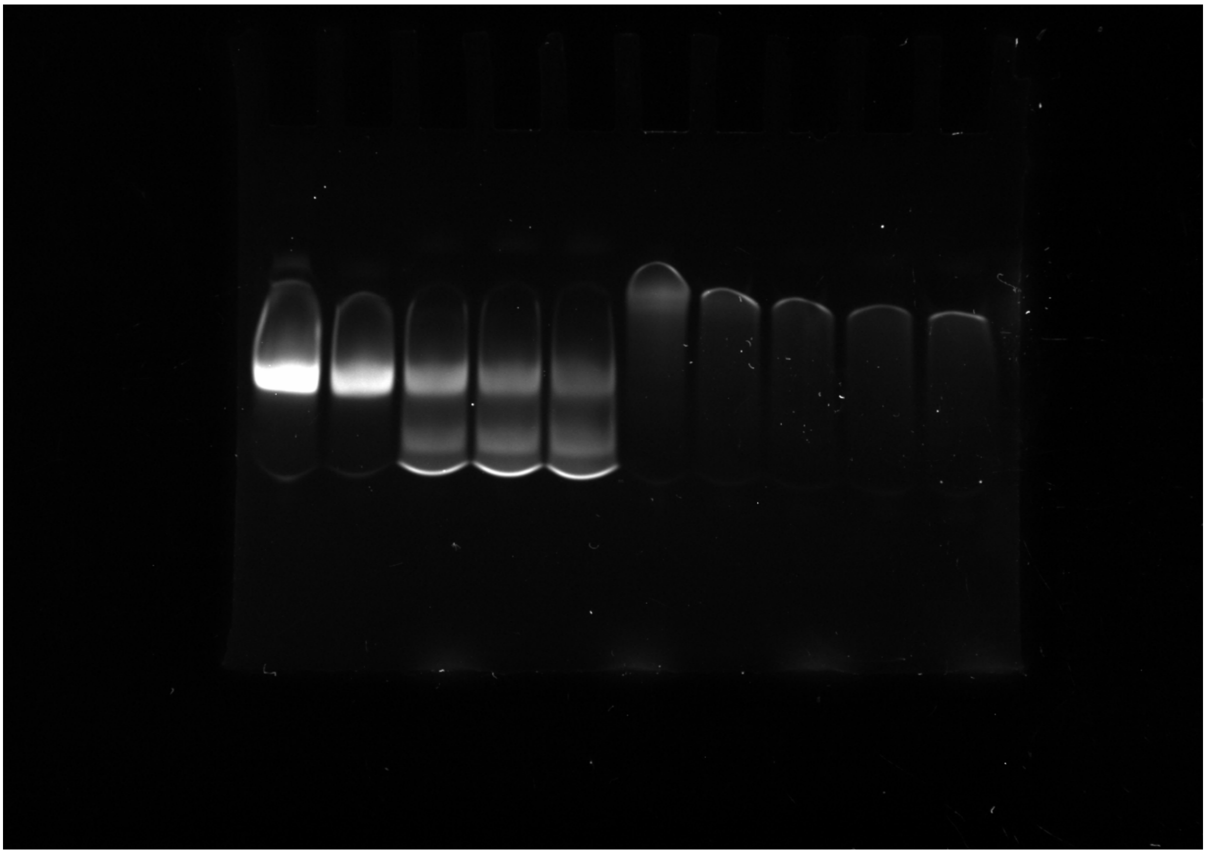

Supplementary Figure 44: Uncropped and unedited gel from **Supplementary Figure 23**, c.
